# *Pas de deux*: An Intricate Dance of Anther Smut and Its Host

**DOI:** 10.1534/g3.117.300318

**Published:** 2017-12-01

**Authors:** Su San Toh, Zehua Chen, Eric C. Rouchka, David J. Schultz, Christina A. Cuomo, Michael H. Perlin

**Affiliations:** * Department of Biology, Program on Disease Evolution, University of Louisville, Kentucky 40292; † Fungal Genomics Group, Broad Institute of MIT and Harvard, Cambridge, Massachusetts 02142; ‡ Department of Computer Engineering and Computer Science, University of Louisville, Kentucky 40292

**Keywords:** host/pathogen interaction, anther-smut fungi, fungal effectors, *Microbotryum*, *Silene*

## Abstract

The successful interaction between pathogen/parasite and host requires a delicate balance between fitness of the former and survival of the latter. To optimize fitness a parasite/pathogen must effectively create an environment conducive to reproductive success, while simultaneously avoiding or minimizing detrimental host defense response. The association between *Microbotryum lychnidis-dioicae* and its host *Silene latifolia* serves as an excellent model to examine such interactions. This fungus is part of a species complex that infects species of the Caryophyllaceae, replacing pollen with the fungal spores. In the current study, transcriptome analyses of the fungus and its host were conducted during discrete stages of bud development so as to identify changes in fungal gene expression that lead to spore development and to identify changes associated with infection in the host plant. In contrast to early biotrophic phase stages of infection for the fungus, the latter stages involve tissue necrosis and in the case of infected female flowers, further changes in the developmental program in which the ovary aborts and a pseudoanther is produced. Transcriptome analysis via Illumina RNA sequencing revealed enrichment of fungal genes encoding small secreted proteins, with hallmarks of effectors and genes found to be relatively unique to the *Microbotryum* species complex. Host gene expression analyses also identified interesting sets of genes up-regulated, including those involving stress response, host defense response, and several agamous-like MADS-box genes (AGL61 and AGL80), predicted to interact and be involved in male gametophyte development.

The interaction between a pathogen/parasite and its host may be characterized as an intricate and intimate dance between two organisms whose interests are in conflict but whose fates are tethered. The “lead” in this dance must balance the need to optimize reproductive success with the dependence on host survival, at least until the music stops and the dance has ended.

The *Microbotryum-Silene* system is a natural plant-pathogen association serving as a model in ecology, epidemiology, and evolution (Bernasconi *et al.* 2009). Members of the *Microbotryum* fungal complex cause anther-smut disease on >100 plant species in the Caryophyllaceae family (Kemler *et al.* 2006; Hood *et al.* 2010). *Microbotryum* species are considered biotrophic, since they colonize living plant tissue and obtain nutrients from living host cells. However, the anther-smut fungi sterilize plants by producing spores in anthers, replacing pollen, and aborting ovaries (Refrégier *et al.* 2008). The best-studied association is *M. lychnidis-dioicae* parasitizing *Silene latifolia* (Refrégier *et al.* 2008). This fungus has served as an important system of investigation for nearly a century (Kniep 1919) and has emerged as a model of fungal speciation and host specialization following host shifts (Kemler *et al.* 2006; Le Gac *et al.* 2007a,b; Refrégier *et al.* 2008; De Vienne *et al.* 2009a,b; Büker *et al.* 2013; Gladieux *et al.* 2015). This system has extended into the area of genomics, with a nearly completed genome assembly in full chromosomes or chromosome arms, as well as expression data (Badouin *et al.* 2015; Fontanillas *et al.* 2015; Perlin *et al.* 2015; Toh *et al.* 2017).

*S. latifolia* is a dioecious flowering plant with dimorphic sex chromosomes where the presence or absence of the Y chromosome controls the appearance and suppression of the appropriate reproductive organs. *S. latifolia* has become an important model for the study of sex determination in plants and the evolution of sex chromosomes (Charlesworth 2013). During *M. lychnidis-dioicae* infection of *S. latifolia* male flowers, dikaryotic fungal hyphae penetrate the host tissue and proceed through the plant causing little or no tissue damage. Upon reaching the flower primordia, the dikarya develop into the teliospores and replace the pollen in the anthers. In contrast, infection of the female host suppresses gynoecium formation and facilitates continued androecium development thus allowing mature anthers to house teliospores in a manner similar to what is seen in a male host (Ruddat *et al.* 1991; Uchida *et al.* 2003, 2005). The codevelopment of the fungus in the floral bud has been studied and characterized, such that the stage of the floral bud (Grant *et al.* 1994) is indicative of the stage at which the fungus is engaged in teliosporogenesis (Farbos *et al.* 1997; Uchida *et al.* 2003; Toh and Perlin 2015). Most of these actions take place in the early floral bud development, between Stages 8 and 11. Hence, we embarked on an effort to tease apart the interaction of the pathogen and its hosts at the transcriptome level by performing RNA sequencing on the discrete stages of the floral tissue based on careful developmental staging (Toh and Perlin 2015).

Simultaneous RNA-Seq (Westermann *et al.* 2012) of host and pathogen allows examination of the transcriptomes in parallel and has been used in compatible and incompatible plant-pathogen systems to uncover pathogenesis and defense-related genes, *e.g.*, in the interactions of rice (*Oryza sativa*) with blast fungus (*Magnaporthe oryzae*) (Kawahara *et al.* 2012), poplar with rust fungus (*Melampsora larici-populina*) (Petre *et al.* 2012), cotton (*Gossypium barbadense*) with wilt fungus (*Verticillium dahliae*) (Xu *et al.* 2011), and canola (*Brassica napus*) interaction with *Leptosphaeria* species (Lowe *et al.* 2014).

Zemp *et al.* (2015) recently produced exciting results showing the influence of *M. lychnidis-dioicae* infection on host gene expression. This work provides data on pathogen-driven changes in host gene expression that could begin to explain the basis for altered host sexual dimorphism. They found sex-specific host responses to pathogen infection and reduced sexual dimorphism in infected *S*. *latifolia*.

In our current study, we compared the gene expression of *M. lychnidis-dioicae* associated with male or female hosts during discrete early floral development stages to the transcriptome of the mated fungal cells. This study provides a broad view of this fascinating phytopathogen system’s gene expression in the developing floral buds during the infection process.

## Materials and Methods

### Host plants and fungal strains

*S. latifolia* seeds were harvested in Summer 2009 from greenhouse grown plants; these were originally from seeds of a field population in Clover Hollow (37.328–80.488) near Mt Lake Biological Station, Virginia, and were kindly provided by M. Hood. Germination and infection of the host plant were performed as previously published (Perlin *et al.* 2015; Toh and Perlin 2015).

Floral buds were staged as described in Toh and Perlin (2015). In addition, the pedicels of the floral cluster were collected as the Floral Stem Tissue. Three floral stem, two Stage 8, two Stage 9, two Stage 10, and five late-stage samples were obtained from two male infected plants and were submitted for RNA-Seq at the Broad Institute for sequencing via Illumina. Two of these late-stage samples were of low quality and not used in the final analysis. Two floral stem, one Stage 7, one Stage 9, one Stage 10, and two late-stage samples were obtained from a single female infected plant and submitted for RNA-Seq.

In analyzing the differential expression data from infected plants, independent changes were considered first before considering changes found in both male and female, since they are biologically very different. However, since there was only one sample for most of the female stages, it is difficult to determine if a change was not present or simply not strong enough statistically to be reported. Hence, we analyzed these as combined samples for floral stem, Stage 8 (in this case, infected male Stage 8 and infected female Stage 7 as the closest comparable pair), Stage 9, Stage 10, and late stage of infection.

### RNA extraction and sequencing

RNA was extracted and processed as previously published (Perlin *et al.* 2015). A minimum of 5 μg of DNA-free RNA was prepared for each desired stage and sent to the Broad Institute for RNA-Sequencing via Illumina. For RNA-Seq, we purified polyA RNA, constructed a strand-specific library for each sample as previously described (Perlin *et al.* 2015), and sequenced each with Illumina technology generating 76 base paired reads. Across the libraries, 96% of reads met the Illumina Passing Filter quality threshold. For the *in planta* samples, only 23% of reads aligned to the *M. lychnidis-dioicae* genome. This was expected as these samples also contain the host *Silene* RNAs. To assemble transcripts for use in annotation, RNA-Seq reads were aligned to the assembly with Blat, and then assembled as described previously (Perlin *et al.* 2015).

RNA-Seq data from different conditions were processed using the Trinity pipeline scripts for differential expression analysis (Grabherr *et al.* 2011; Haas *et al.* 2013). For analysis of fungal gene expression, RNA-Seq reads from each sample were aligned to the predicted M. *lychnidis-dioicae* coding sequences from the annotated genome (Perlin *et al.* 2015) using bowtie (Langmead *et al.* 2009). Transcript abundances were estimated using RSEM (Li and Dewey 2011). Differentially expressed genes between each pair of conditions were identified using edgeR with TMM normalization (Robinson *et al.* 2010; Kadota *et al.* 2012) with a corrected p-value cutoff of 1e−5 or 1e−2 (Supplemental Material, Table S1), and the list of fungal differentially expressed genes (DEGs) at 1e−5 cutoff is found in Table S2. For analysis of host gene expression, a reference transcript set was created; RNA-Seq reads from all infected and uninfected host samples were pooled together and assembled using Trinity (Grabherr *et al.* 2011). Assembled transcripts were filtered based on the alignment with the *M. lychnidis-dioicae* fungal genome (Fontanillas *et al.* 2015; Perlin *et al.* 2015) to eliminate fungal transcripts, and transcripts predicted to encode <200 amino acids and that lacked PFAM domains were also removed. Read alignment and differential expression analysis of host genes were then evaluated as described for fungal gene expression, *i.e.*, RSEM and edgeR were used for gene expression quantification and differential analysis with filtered Trinity transcripts, with a more detailed description provided below.

The available fungal and host genomes [fungal, *M. lychnidis-dioicae*, 29.9 Mb (GenBank Assembly Accession GCA_900015465.1); host, *Silene latifolia*, Assembly ASM141213v1, 665.28 Mb (Muyle *et al.* 2012)] were used to map the reads from RNA-Seq analysis. The conditions are listed in Table S3. A summary of short read alignments for each sample is found in Table S7. From the expression analysis we obtained 3.6 billion Illumina paired-end reads across the 27 sequenced libraries, corresponding to 363.6 Gigabases (Gb) of RNA-seq data that are publicly available in NCBI (BioProject ID: PRJNA246470). On average, for the uninfected male and female samples, 75–76% of the reads were mapped to the *S. latifolia* reference genome (Assembly ASM141213v1) and transcriptome (Muyle *et al.* 2012) (Table S4). On the other hand, for some of the other samples, the low mapping rates of 20–50% were in part due to incomplete transcript information. Further analyzing the data, the unique reads were collapsed using fastx_collapser (https://pods.iplantcollaborative.org/wiki/display/DEapps/Fastx+Collapser) to see what those that have the highest counts represent. The majority were rRNA sequences based on blast similarity. However, the rRNA sequences are not available for *S. latifolia*, so an exact measure was not possible. Thus, the number of usable reads was smaller than it might otherwise be. It could also be that these rRNAs are polyadenylated as well (Zhuang *et al.* 2013; Roundtree *et al.* 2017).

In order to assign function to the *de novo* transcripts, a database of nonredundant plant proteins was constructed based on the NCBI nr database. For this process, each of the files from the nr database was downloaded from the NCBI ftp site (ftp://ftp.ncbi.nlm.nih.gov/blast/dbon9/29/2015). The GenIdentifier (GI) accessions for plant proteins were downloaded from the NCBI protein database (http://www.ncbi.nlm.nih.gov/protein) in the following manner. First the query ((all [filter])) AND “green plants”[porgn:__txid33090] was used to return all green plant proteins (5,821,183 such sequences). The GIs for each of these sequences were then downloaded using the “Send To” link with the options Destination “File” and Format “GI List.” The resulting file was stored as sequence.gi.txt. The NCBI blast executable was downloaded from ftp://ftp.ncbi.nlm.nih.gov/blast/executables/blast+/2.2.31/ncbi- blast-2.2.31+-win64.exe. Then the downloaded nr database was filtered for only the GIs in the sequence.gi.txt using the custom script to run blastdbcmd.

A total of 583 plant_<num>.faa files result. These were then concatenated together into a single file ALLPLANTSEQ.faa. Many of the headers contain redundant headers for the exact same sequence. Therefore, redundant headers were removed using a custom script. Using the nonredundant ALLPLANTSEQ.faa, a blastable database was constructed using the command Makeblastdb.

The Trinity contigs were then compared against the database of known plant sequences into an XML output for use by blast2go (Gotz *et al.* 2008) using the command: blastx -db PLANT_NR_UPDATED -query Mvio_trinity_filtered.fas \–evalue 0.001 -outfmt 5 - max_target_sequations 20 -out blastALL.xml.

The raw fasta file Mvio_trinity_filtered.fas (which contains 77,520 different contigs) was uploaded into Blast2go basic v4.0.7 (downloaded from https://www.blast2go.com) to add annotations. Annotations from the file blastALL.xml were added to the blast2go project using the File… Load… Load Blast Results.

**Figure 1:**
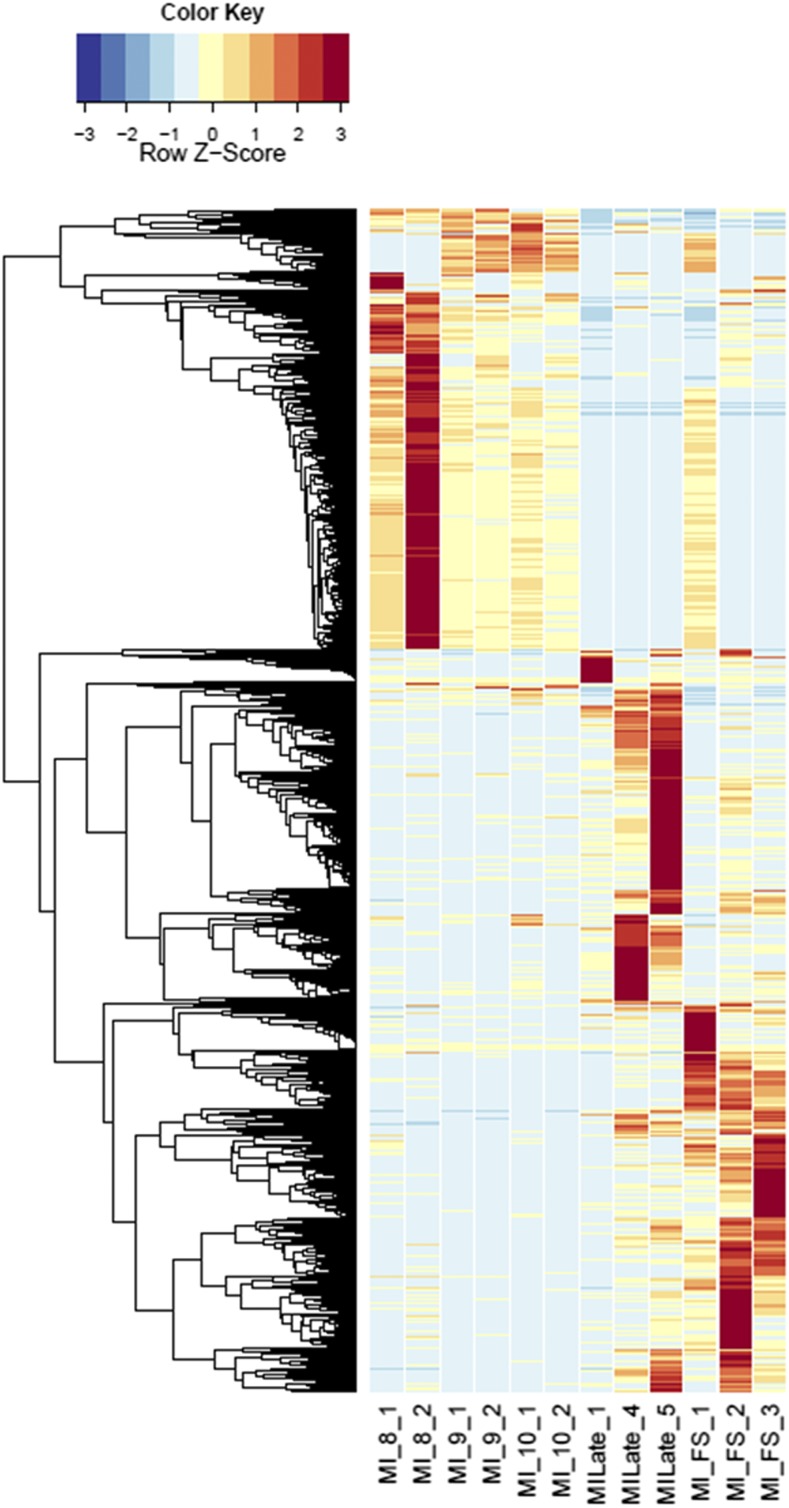
Heatmaps of host gene expression: infected male plant comparisons. fdr <1e−2, 2254 genes. MI, infected male plants at stages indicated.

**Figure 2:**
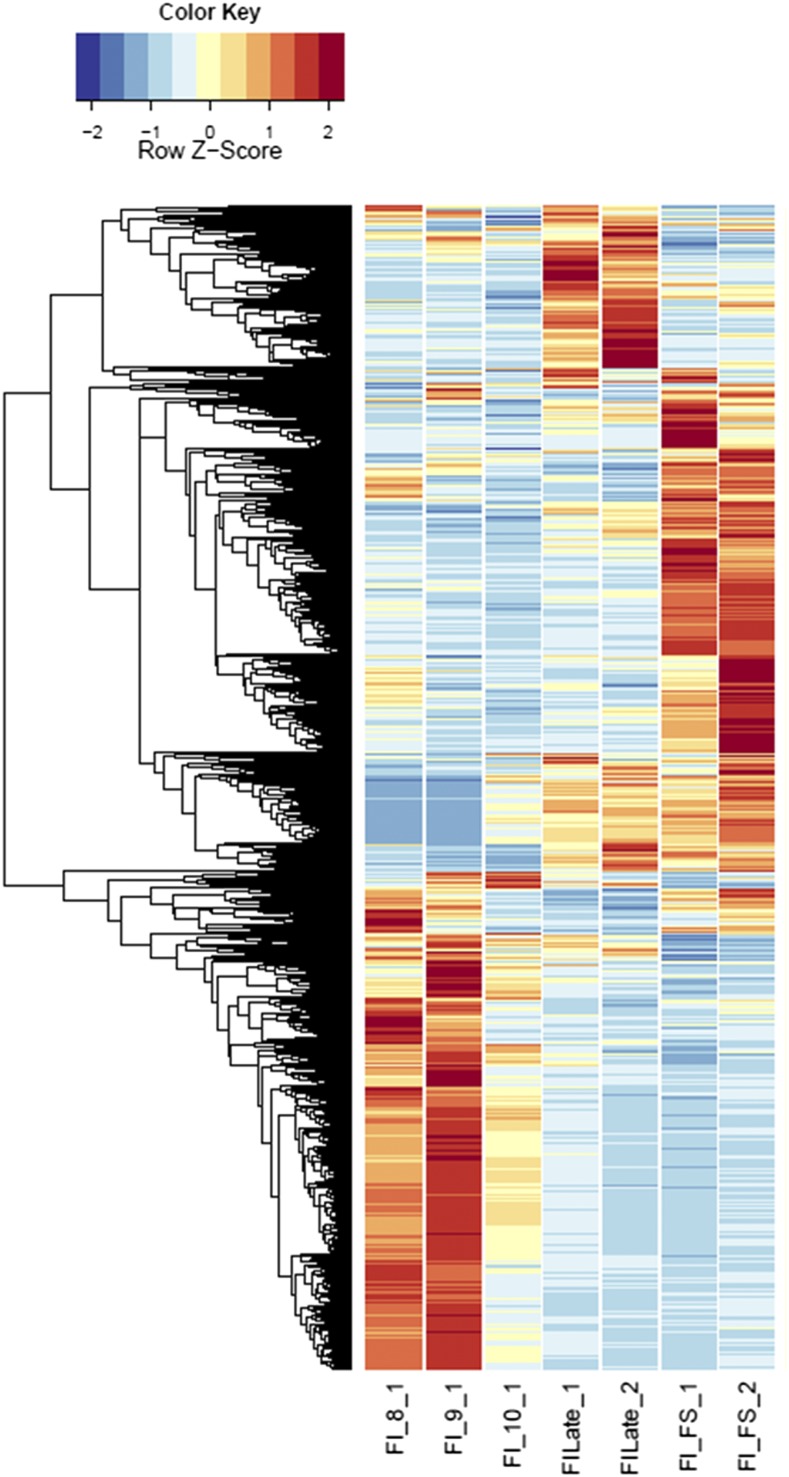
Heatmaps of host gene expression: infected female plant comparisons. fdr <1e−2, 9629 genes. FI, infected female plant at stages indicated.

Annotations were then exported as an .annot file within blast2go. For each of 48 comparisons, significantly DEGs were determined using custom scripts. A *q* value cutoff of 0.05 was used as significant measures. The number of significant host DEGs is shown in Table S3. The top five up-regulated and top five down-regulated DEGs in each comparison were extracted. A counts matrix for each of these genes was constructed, and a heatmap was drawn to show the log_2_ of the edgeR normalized raw expression values across all samples (Figure S1). Heatmaps for all infected host gene comparisons are found in Figure 1 and Figure 2, and in Figure S1, Figure S2, Figure S3, Figure S4, and Figure S5.

### Venn diagrams

Venn diagrams were generated using Venny (Oliveros 2007). The edgeR data were sorted to extract the list of the predicted fungal genes (Perlin *et al.* 2015) that were significantly up-regulated or down-regulated (false discovery rate, FDR <1E−5) against a reference condition in the pairwise analysis.

### Gene set enrichment analysis (GSEA) preranked

GSEA (Mootha *et al.* 2003; Subramanian *et al.* 2005) was performed to discover the possible enrichment of PFAM domains, gene ontology terms (Conesa *et al.* 2005), and KEGG pathway maps (Kanehisa *et al.* 2016) in the conditions studied using the GenePattern software (Reich *et al.* 2006). In addition, the predicted small secreted proteins (SSPs, <250 amino acids long), secreted proteins (SPs, >250 amino acids long), and proteins unique to *Microbotryum* based on orthomcl analysis (Li *et al.* 2003; Perlin *et al.* 2015) were included as gene sets. The resulting gene set list (.gmt) consisted of 3651 gene sets, with a range of 1–1116 members. Ranked lists (.rnk) of every pairwise comparison made were processed by the GSEAPreranked module software. When running the software, the algorithm was set to allow minimum gene set of one and maximum of 1200. Gene sets that were significantly enriched at FDR <25% or nominal p-value <1% were listed and examined for further hypothesis-generation.

Given the annotated gene ontology (GO) categories for each of the Trinity assembled transcripts, the DEGs were searched for significant GO enrichments using a hyper-geometric test modified from categoryCompare (Flight *et al.* 2014). Significance was determined using a p-value cutoff of 0.001 and a minimum gene inclusion number of 5. The number of significant GO categories for each comparison is shown in Table S3.

### Data availability

Fungal strains and host samples are available upon request. All sequence data for this study are available in NCBI under bioproject PRJNA246470. Supporting information is found in Table S1, Table S2, Table S3, Table S4, Table S5, Table S6, Table S7, and Table S8, which contain tables of Fungal DEGs, summary of Fungal GSEA, host DEGS, and host GSEA analysis/GOs, as well as in Figure S1, Figure S2, Figure S3, Figure S4, Figure S5, Figure S6, Figure S7, Figure S8, Figure S9, Figure S10, Figure S11, Figure S12, Figure S13, and Figure S14, which contain additional heatmaps for comparisons in this study.

## Results

**Figure 3:**
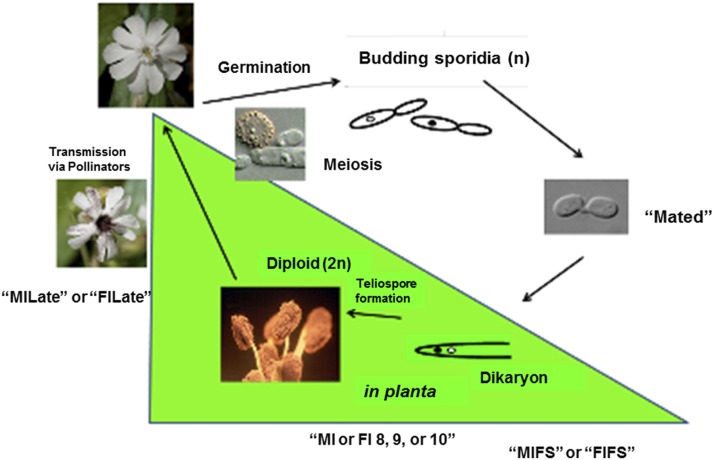
Conditions/stages of *M. lychnidis-dioicae–S. latifolia* system examined by RNA-Seq. *In planta* stages of the interaction were examined by RNA-Seq to analyze differentially expressed fungal and host genes at each stage. For fungal analyses, differential expression was compared, in most cases with the *in vitro* condition of mated haploid sporidial cells (“Mated”), MIFS or to FIFS. MIFS, infected male *S. latifolia* floral stem; FIFS, infected female *S. latifolia* floral stem; MI or FI 8, 9, or 10, infected male (MI) or female (FI) *S. latifolia* buds at the respective developmental stage indicated (Toh and Perlin 2015); MILate or FILate, infected male or female flowers, respectively, in the late stages of infection, where opened flowers show visible teliospores. Additional conditions not shown: MU, uninfected male plants; FU, uninfected female plants; MIFI designations in the text refer to RNA-Seq data pooled for male and female infected plants for a given condition for Gene Enrichment Analysis (*e.g.*, MIFIFS). This figure was adapted, with permission, from our previously published work (Figure 1; Perlin *et al.* 2015).

Floral development stages of *S. latifolia* examined in this study (Figure 3 and Table S3) included those where the fungal transition from biotrophic dikaryotic “migrant” shifted to entering the floral meristem, and into the androecium where continued dikarya development replaced pollen with teliospores, or into the female where the fungal cells have the additional task of reprogramming development of the gynoecium into an androecium. Thus, to capture changes in gene expression during fungal morphological transformation we targeted analyses on the early stages of floral development. Analysis of the fungal transcriptomes in the different sexes of host plants may provide additional insights as to adaptations of the fungus to accommodate the different milieus where its development must occur. To best capture this process, RNA-Seq of male or female infected floral stems; male infected bud stages 8, 9, and 10; female infected bud stages 7, 9, and 10; and male and female infected late-stage opened bud; as well as male and female uninfected buds were analyzed and compared. For fungal genes, a heat map of correlation coefficient of fungal gene expression levels (FPKM) among all *in planta* samples (see Figure S6) shows similarity of expression among closely related stages.

### Differential fungal gene expression in infected floral buds

**Figure 4:**
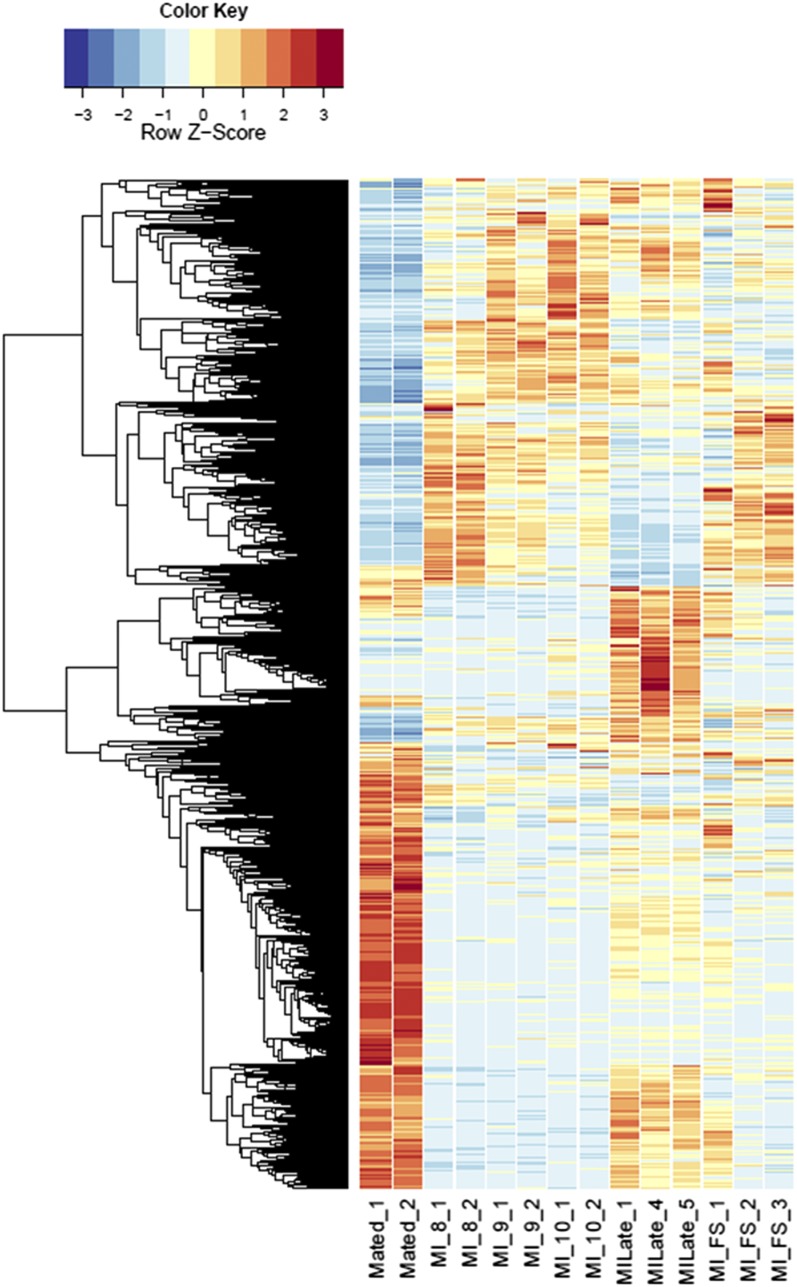
Heatmaps of fungal gene expression in infected male plant comparisons From all comparisons, 3496 genes. MI, infected male plants at stages indicated.

All fungal RNA-Seq data were compared, after FPKM calculations, against *in vitro* mated haploid cells of *M. lychnidis-dioicae* strains p1A1 and p1A2 as reference (Toh *et al.* 2017) or male or female infected floral stems. Mating of haploid sporidial cells occurs outside the plant and is a prerequisite for infection and the remaining downstream events; thus, mating serves as one of the last stages before plant penetration. This provides the opportunity to make all other comparisons for expression among the postmating stages (Figure 3) after infection. Comparison of infected male stages with mated cells indicated a total of 1645 (22.3%) and 1756 (23.8%) fungal genes were significantly up- and down-regulated (FDR <1E−5), respectively (Figure S7). About 30% of both groups applied to all the floral stages (Figure 4). When infected male stages were compared to male infected floral stems, the largest group of genes either up- (Figure S8A) or down-regulated (Figure S8B) were those shared among all three discrete bud stages (8, 9, and 10) and late-stage infected buds (Table S1 and Table S2). Similarly, the next largest group of genes found to be in common was specifically for genes up-regulated or down-regulated among the discrete bud stages for infected males, with Stage 10 providing the largest overlap with the late infected samples.

**Figure 5:**
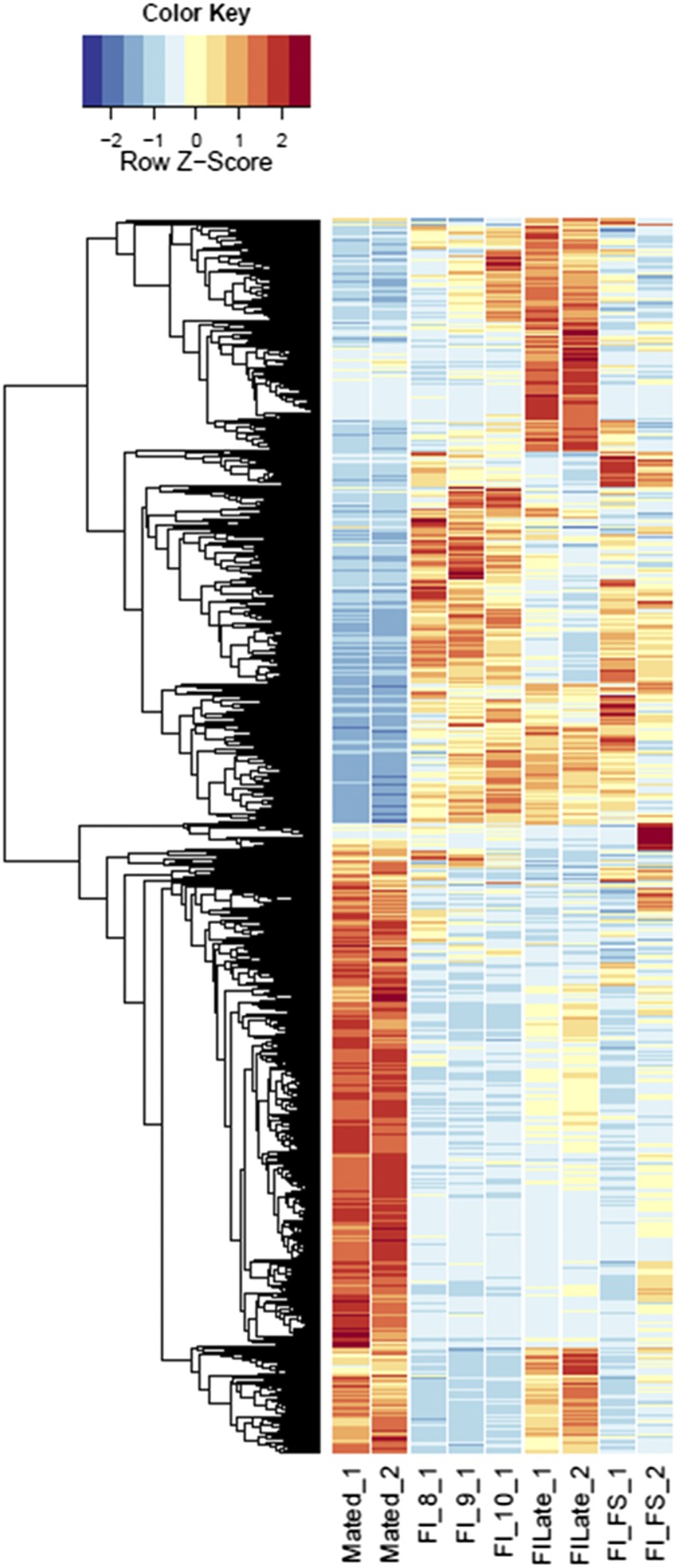
Heatmaps of fungal gene expression in infected female plant comparisons From all comparisons, 2724 genes. FI, infected female plant at stages indicated.

Comparing female infected floral buds to mated, *in vitro* samples (Toh *et al.* 2017) indicated a total of 1322 (17.9%) fungal genes were significantly up-regulated (Figure 5 and Figure S9A) and 1382 (18.8%) genes were significantly down-regulated (Figure 5 and Figure S9B). About 30% of the up-regulated and 23.5% of the down-regulated group of genes applied to all the floral stages. When comparing these stages with female infected floral stems, only 83 and 155 genes were up- and down-regulated, respectively (Figure S10). Most of the changes (71 and 80% up- and down-regulated, respectively) were exclusively in the late infected samples. However, this result needs to be interpreted with caution as there was only one sample for each of Stages 7, 9, and 10 female infected floral buds. This may have reduced the sensitivity and reproducibility in picking up slightly differentially regulated genes.

Due to limited female infected sample size (both in terms of biological replicates and stages), we performed a combined analysis in an attempt to detect additional differentially regulated fungal genes. Based on edgeR smear plots of Log2 fold change against average Log2 counts per million for male against female at each stage, floral stem, 7, 9, 10, and Late, respectively (not shown), only a limited number of genes were differentially regulated, compared to each corresponding infected male stage. Hence, samples were combined stage-wise for further comparison, as male and female effects were not substantial. For convenience of analysis, infected female Stage 7 was grouped with infected female Stage 8 as the same condition, since they were of the same size buds. The remaining infected female samples were grouped with the corresponding infected male samples. An additional 58 (18.4%) and 61 (12.8%) genes were found to be up- and down-regulated, respectively, when the male and female samples were combined for each stage (*i.e.*, 8, 9, 10, and late infected) and compared to floral stem (Figure S11), thereby providing support for this approach to the analyses.

### Global gene set enrichment

We used preranked gene set enrichment analysis (GSEA Preranked analysis) with GenePattern software (Reich *et al.* 2006) to independently look at functional categories of fungal gene expression in male and female and for these data combined as biological replicates (Table S5, complete list). Table 1 (combined sexes) and Table 2 (individual sexes) list enriched fungal gene sets that were up- and down-regulated, respectively, *in planta* compared to mated sporidial isolates (designated as “Mated”; Figure 4 and Figure 5; Toh *et al.* 2017). Enriched gene sets only found in one sex and not in the combined analyses may indicate the other sex has regulation of those genes in the opposite direction, thus negating the significance of differential regulation. Gene sets that were found in the combined analysis and not in the individual sex would suggest that the change may be subtle and only detectable with an increased number of biological replicates.

**Table 1:** List of enriched GO terms and Pfam domains in fungal gene sets that were up-regulated *in planta* compared to Mated, arranged in order of decreasing magnitude of normalized enrichment score (NES)

Common Enrichment in Genes Upregulated in Both Male and Female over >1 Stage When Compared to Mated, FDR *q* Value < 0.05
SMALL SECRETED PROTEIN*^a^*
UNIQUE TO MVIO
SECRETED PROTEIN
PF00295.10|GLYCO_HYDRO_28[GLYCOSYL HYDROLASES FAMILY 28]
GO:0006412[TRANSLATION > BP]
GO:0006281[DNA REPAIR > BP]
PF00125.17|HISTONE[CORE HISTONE H2A/H2B/H3/H4]
GO:0003735[STRUCTURAL CONSTITUENT OF RIBOSOME > MF]
GO:0005840[RIBOSOME > CC]
PF00012.13|HSP70[HSP70 PROTEIN]
PF06723.6|MREB_MBL[MREB/MBL PROTEIN]
GO:0000786[NUCLEOSOME > CC]
PF01095.12|PECTINESTERASE[PECTINESTERASE]
**Enrichment in genes up-regulated in different stages*^b^* compared to Mated, FDR *q* value <0.05**
GO:0006334[NUCLEOSME ASSEMBLY > BP] (**infected male plants Stages 8–10, combined infected male and female Stage 10**)
PF07731.7|CU-OXIDASE_2[MULTICOPPER OXIDASE] (**infected male plants Stages 8, combined infected male and female Stage 8**)
PF07732.8|CU-OXIDASE_3[MULTICOPPER OXIDASE] (**combined infected male and female Stages 8 and 9**_)_
PF00394.15|CU-OXIDASE[MULTICOPPER OXIDASE] (**combined infected male and female Stages 8 and 10**)
PF03169.8|OPT[OPT OLIGOPEPTIDE TRANSPORTER PROTEIN ](**infected female plant Stage 7**)
PF07250.4|GLYOXAL_OXID_N[GLYOXAL OXIDASE N-TERMINUS]] (**combined infected male and female Stage 8, infected female Stages 9 and 10**)
PF09118.4|DUF1929[DOMAIN OF UNKNOWN FUNCTION (DUF1929) ] (**combined infected male and female Stage 9**)
GO:0005634[NUCLEUS > CC] (**infected male plant Stage 10, combined infected male and female Stage 10 and late stage 9**)
GO:0003677[DNA BINDING > MF] (**infected male plant Stage 10, combined infected male and female Stage 10**)
GO:0006950[RESPONSE TO STRESS > BP] (**infected male plant Stages 9 and 10**)
PF09118.4|DUF1929[DOMAIN OF UNKNOWN FUNCTION (DUF1929)] (**infected female plant Stage 10**)
GO:0006414[TRANSLATIONAL ELONGATION > BP] (**combined infected male and female Stage 10**)
PF11754.1|VELVET[VELVET FACTOR] (**combined infected male and female Stage 10**)
PF00082.15|PEPTIDASE_S8[SUBTILASE FAMILY] (**infected female plant late stage**)
PF06280.5|DUF1034[FN3-LIKE DOMAIN (DUF1034)](**infected female plant late stage**)
PF01490.11|AA_TRANS[TRANSMEMBRANE AMINO ACID TRANSPORTER PROTEIN] (**infected female plant late stage**)
None (**infected male floral stem, infected female floral stem, combined infected male and female floral stem**)
None with GO terms or PFAM domains (**infected male plant late stage**)

a Complete listings of Conditions, Gene Set Enrichments, number of members in set, magnitude of NES, and FDR *q*-val are found in Table S5.

b Stages where enriched indicated in parentheses in bold.

**Table 2:** List of enriched GO terms and Pfam domains in fungal gene sets that were among genes down-regulated in plants when compared with Mated with no order of NES

Common Enrichment in >2 of Mated *vs.* MI, FI, and MIFI Stages, FDR *q* Value < 0.05
PF10340.2|DUF2424[PROTEIN OF UNKNOWN FUNCTION (DUF2424)]*^a^*
PF05572.6|PEPTIDASE_M43[PREGNANCY-ASSOCIATED PLASMA PROTEIN-A]
PF00501.21|AMP-BINDING[AMP-BINDING ENZYME]
PF05686.5|DUF821[*ARABIDOPSIS THALIANA* PROTEIN OF UNKNOWN FUNCTION (DUF821)]
PF03583.7|LIP[SECRETORY LIPASE]
PF02129.11|PEPTIDASE_S15[X-PRO DIPEPTIDYL-PEPTIDASE (S15 FAMILY)]
TIGR00976[/NOND: HYDROLASE COCE/NOND FAMILY PROTEIN]
PF02129.11|PEPTIDASE_S15[X-PRO DIPEPTIDYL-PEPTIDASE (S15 FAMILY)] PF08613.4|CYCLIN[CYCLIN]
**Enrichment in genes down-regulated in different stages*^b^* compared to Mated, FDR *q* value <0.05**
PF12464.1|MAC[MALTOSE ACETYLTRANSFERASE] (**combined infected male and female floral stem**)
PF00383.15|DCMP_CYT_DEAM_1[CYTIDINE AND DEOXYCYTIDYLATE DEAMINASE ZINC-BINDING REGION (**combined infected male and female floral stem**)
PF00621.13|RHOGEF[RHOGEF DOMAIN] (**combined infected male and female floral stem**)
PF11899.1|DUF3419[PROTEIN OF UNKNOWN FUNCTION (DUF3419)] (**combined infected male and female floral stem**)
PF00664.16|ABC_MEMBRANE[ABC TRANSPORTER TRANSMEMBRANE REGION] (**combined infected male and female Stage 9**)
PF02815.12|MIR[MIR DOMAIN] (**combined infected male and female Stage 9**)
PF06609.6|TRI12[FUNGAL TRICHOTHECENE EFFLUX PUMP (TRI12)] (**infected male plant Stage 10**)
PF08530.3|PEPX_C[X-PRO DIPEPTIDYL-PEPTIDASE C-TERMINAL NON-CATALYTIC DOMAIN] (**infected male plant late stage**)
PF07859.6|ABHYDROLASE_3[ALPHA/BETA HYDROLASE FOLD] (**infected female plant late stage**)

a Complete listings of Conditions, Gene Set Enrichments, number of members in set, magnitude of NES, and FDR *q*-value are found in Table S5.

b Stages where enriched indicated in parentheses in bold.

Fungal gene set enrichments that were detected only in the infected male were Nucleosome Assembly (GO:0006334) and Response to Stress (GO:0006950), while the fungus in female plants had enrichment for Oligopeptide transporters (PF3169) and glyoxal oxidase N-terminus (PF07250) in the early stages and Peptidase S8 (PF00082) and domains of unknown function (PF09118 and PF06280) in the later stages (Table S5). Multicopper oxidases were detected in the combined analysis, identifying three genes (MVLG_01868, 02184, and 03092). Upon examination of the normalized counts as listed in Table S6, we discovered that for these three genes, they were also high in female, but since there was only one biological replicate for most stages in female, differential expression likely did not have the same sensitivity. This observation highlighted the importance of analyzing the combined data, and also emphasized the need for reliable biological replicates for optimal analysis of the gene expression data.

In contrast, among the genes down-regulated during infection, the gene sets related to peptidases and transporters were significantly enriched (Table 2). One of the identified domains of interest was the secretory lipase domain (PF03583), a gene family expanded in *M. lychnidis-dioicae* compared to related species (Perlin *et al.* 2015). Genes with this domain were found to be significantly up-regulated in mating cells exposed to phytol (Perlin *et al.* 2015; Toh *et al.* 2017). By contrast, these genes appear down-regulated in fungus infecting plants, suggesting that these proteins have specific functions right after mating, supporting our initial hypothesis that they may be directly involved in hyphae formation, as in the response elicited by exposure to phytol.

The most prominent up-regulated fungal gene set enrichments in plants were the SSPs, SPs, and proteins unique to *M. lychnidis-dioicae* or *Microbotryum* species (Perlin *et al.* 2015). We extracted the list of these proteins that contributed to common enrichment in both infected male (MI) and infected female (FI) at each specific stage in the analysis and checked whether they were up-regulated in all stages or only in discrete stages. Table 3 lists the number of common genes found across all plant stages and the number of unique genes specific to discrete stages. For secreted proteins and unique proteins, there appeared to be a large group used exclusively in the infected male and female late stages (numbers are found in parentheses). This may suggest that the formation of teliospores involves more secreted proteins and unique proteins than other stages. Consistent with this, there are not as many gene sets pertaining to known domains found in the late stage (Table 1). This is significant since there were many genes that were up-regulated in the late stage and yet few gene sets were identified (Figure S7A and Figure S9A).

**Table 3:** Number of common proteins in each core-enriched gene set up-regulated in all plant stages when compared to Mated

Types of Protein	Male Plants	Female Plants	Combined
Small secreted protein (SSP)*^a^*	15/32	17/30	17/33
Secreted protein (SP)	18/41 (5)*^b^*	15/47 (10)*^b^*	23/44 (7)*^b^*
Unique proteins	74/184 (57)*^b^*	63/145 (35)*^b^*	77/167 (51)*^b^*

a Size threshold for SSP is <250 aa.

b Numbers in parentheses indicate genes used exclusively in the Late stage.

SSPs, SPs, and unique proteins that contribute to the enrichment in each sex at each stage were compared (Figure S12). Almost all the SSPs (85.3% in common) and SPs (79.5% in common) were the same in each stage, though the proportion of SPs found up-regulated in female only (20.4% of all SPs) was higher than in male plants (4%). Similarly, the Unique (to *Microbotryum*) proteins in male plants tended to be a more limited group.

To elucidate potential roles in mating and in teliosporogenesis for some of the same genes, we compared (Figure S13) the list of SSPs, SPs, and Unique proteins found up-regulated in plants (all *in planta* stages) to those up-regulated in *in vitro* stages (mated cells exposed to phytol for 12–48 hr; those up-regulated in Mated and Nutrient-limited, *i.e.*, haploid under conditions conducive for mating) when normalized against p1A1 Rich (p1A1 strain on YPD medium) (Toh *et al.* 2017).

It appeared that the SSPs involved in Mated and Nutrient-limited samples were also expressed either in phytol-treated mated cell samples or *in planta* (Table S5). While the level of expression may be very different, it is noteworthy that all the SSPs available for subsequent development, whether during hyphae production or for teliosporogenesis, were activated once the environment was conducive for mating. It is tempting to suggest that deficiency in these genes may have a profound impact on the ability of the fungal cells to complete their sexual life cycle.

There was a larger group of SPs (32.3%) and unique proteins (47.1%) expressed exclusively in Mated and Nutrient-limited samples than in phytol-treated mated cells or in the host plants. This supports the hypothesis that the mating system of *M. lychnidis-dioicae* is associated with many proteins of unknown function. Expression in host plants was more similar to phytol-treated mated cells than with Mated and Nutrient-limited samples in all three categories (Figure S13), suggesting that treatment with phytol was probably a close approximation of dikaryon extension induced in the host plants, rather than a different response mechanism.

### Gene set enrichment between stages

To capture codevelopmental stages of fungal infection during floral development, we applied the GSEA Preranked analysis and found significant enrichment of gene sets among the down-regulated genes in the infected male late stage when compared to earlier floral stages (Table 4). Down-regulation of functions related to active cellular growth as an enriched group during development was noted (*e.g.*, KO04141; GO:0003677; GO:0005737; PF07724.7; PF00004.22; PF00158.19; GO:0006334; GO:0000786; PF00125.17). This supports the idea that late in floral developmental, pathogen teliosporogenesis is nearly complete. Consistent with this notion, enrichment was more notable in Stage 10 and compared to late stage (Table 4) suggesting that cellular activities were the strongest at Stage 10 and then decreased at the late stage after teliospore development was completed. The most significant gene set enrichments in all infected developmental stages compared with Mated *in vitro* sample, *i.e.*, those ranked by GSEA nonparametrically, were SSPs, SPs, glycosyl hydrolase family 28, oligopeptide transporter family, and heat shock protein 20. Previously, the *M. lychnidis-dioicae* genome was reported to be enriched in the number of glycosyl hydrolase genes compared to related species (Perlin *et al.* 2015). It is therefore noteworthy that through the analysis presented here, glycosyl hydrolase family 28 is the only glycosyl hydrolase family identified in these enrichments; it thus may be the single most important hydrolase family for teliosporogenesis and requires strict regulation. This family was down-regulated in Mated cells in comparison with infected female Stages 9 and 10, infected male Stage 8, and the combined infected male and female Stage 8, while being down-regulated in the combined infected male and female late stage relative to the corresponding combined infected floral stem.

**Table 4:** List of enriched GO terms and Pfam domains in fungal gene sets that were down-regulated in late stage of floral development compared to earlier plant stages

Common Enrichment in >1 Stage Across >1 Set of Comparisons, *q* Value < 0.05
SMALL SECRETED PROTEIN*^a^*
SECRETED PROTEIN
PF00295.10|GLYCO_HYDRO_28[GLYCOSYL HYDROLASES FAMILY 28]
PF03169.8|OPT[OPT OLIGOPEPTIDE TRANSPORTER PROTEIN]
PF00011.14|HSP20[HSP20/ALPHA CRYSTALLIN FAMILY]
**Enrichment compared with corresponding Late stage*^b^***
KO04141[PROTEIN PROCESSING IN ENDOPLASMIC RETICULUM] (**infected male Stage 8, combined infected male and female Stage 8**)
PF01095.12|PECTINESTERASE[PECTINESTERASE] (**infected male Stage 8, combined infected male and female Stages 8 and 9**)
GO:0003677[DNA BINDING > MF] (**infected male Stage 10, combined infected male and female Stage 10**)
GO:0005737[CYTOPLASM > CC] (**infected male Stage 10, combined infected male and female Stages 9 and 10**)
PF07724.7|AAA_2[AAA DOMAIN (CDC48 SUBFAMILY)] (**infected male Stage 10, combined infected male and female Stages 9 and 10**)
PF10431.2|CLPB_D2-SMALL[C-TERMINAL, D2-SMALL DOMAIN, OF CLPB PROTEIN] (**infected male Stage 10, combined infected male and female Stages 9 and 10**)
PF00004.22|AAA[ATPASE FAMILY ASSOCIATED WITH VARIOUS CELLULAR ACTIVITIES (AAA)] (**infected male Stage 10, combined infected male and female Stages 9 and 10**)
PF00158.19|SIGMA54_ACTIVAT[SIGMA-54 INTERACTION DOMAIN] (**infected male Stage 10, combined infected male and female Stages 9 and 10**)
GO:0006334[NUCLEOSME ASSEMBLY > BP] (**combined infected male and female Stage 10**)
GO:0000786[NUCLEOSOME > CC] (**combined infected male and female Stage 10**)
PF00125.17|HISTONE[CORE HISTONE H2A/H2B/H3/H4] (**combined infected male and female Stage 10**)

a Complete listings of Conditions, Gene Set Enrichments, number of members in set, magnitude of NES, and FDR *q*-value are found in Table S5.

b Stages where enriched indicated in parentheses in bold.

### Gene set enrichments for host

#### Global differences of male uninfected *vs.* female uninfected hosts:

To identify DEGs at distinct stages, we mapped reads to a set of host transcripts (*Materials and Methods*) (Table S7). A *q* value cutoff of 0.05 was used as a significant measure for the 48 comparisons (Table S1). For a baseline comparison of uninfected male *vs.* female host plants, two different comparisons were made. The first included samples MU_8_1, MU_9_1, and MU_9_2 in the uninfected male group while the second comparison also included MU_FS_1 and MU_FS_2 in the group. A correlation heatmap made from correlation coefficients between uninfected samples using host genes assembled by trinity (Figure S14) clearly showed distinct groupings of the uninfected male stages compared to the groupings of uninfected female stages. There were 1355 DEGs (1315 Up and 40 Down) in the comparison of uninfected male *vs.* uninfected female and 784 DEGs (741 Up, 43 Down) in the comparison that also included floral stems (Table S1). No GO terms were enriched in these comparisons, but there were some interesting examples of predicted genes that were up-regulated in male plants, *e.g.*, several ABC transporters, phytochromes, and two MADS-box proteins (MADS-box CMB1-like; MADS-box 3/MADS-box transcription factor 6), associated with biological processes such as floral meristem determinancy, floral organ development, and floral whorl morphogenesis. Furthermore, F-box genes likely involved in flower development as well as a variety of genes associated with transposable element and retroelement activity were up-regulated.

#### Global differences of infected *vs.* uninfected hosts:

GO terms of enrichment (Table S8) for all the comparisons of host gene DEGs involving infected plants revealed broad categories of enriched genes. For example, infected *vs.* uninfected male plants (see Figure S2 and Table 5) identified Catalytic Activity (GO:0003824; Ribonuclease P, GTPase activity); Cellular Metabolic Processes (GO:0044237; ergosterol biosynthesis); Single-Organism Process (GO:0044699; lipid metabolic process, apoptosis); Transposase (GO:0004803); and Binding (GO:0005488; includes DNA, RNA, and Protein Binding). The category Binding was a conserved difference between male infected *vs.* male uninfected plants in the fungal stem (FS), and Stages 8 and 9, while Dioxygenase Activity category (GO:0051213) differed between MI9 and MU9.

**Table 5:** List of enriched GO terms in host gene sets that were differentially expressed comparing infected *vs.* uninfected male plants, in order of increasing magnitude of p-value

Infected Male *vs.* Uninfected Male Plants
Catalytic Activity (GO:0003824 Ribonuclease P, GTPase activity)
Cellular Metabolic Processes (GO:0044237; ergosterol biosynthesis)
Single-Organism Process (GO:0044699; lipid metabolic process, apoptosis)
Transposase (GO:0004803; p-element encoded)
Transposition, DNA-mediated (GO:0006313; Class II transposition, p-element excision, Tc1 or Tc3 mariner)
Cytoplasmic Part (GO:0044444)
Single-organism Transport (GO:0044765; protein import into nucleus)
Heterocyclic Compound Binding (GO:1901363; DNA or RNA binding)
Organic Cyclic Compound Binding (GO:0097159)
Ion Binding (GO:0043167)
Cellular Amino Acid Biosynthetic Process (GO:0008652)
Oxidoreductase Activity (GO:0016491)
Inclusion of 1 atom of Oxygen (GO:0016709)
Protein Ser/Thr Kinase Activity (GO:0004674)
Protein Phosphorylation (GO:0006468)
DNA Integration (GO:0015074)
Binding (GO:0005488; includes DNA, RNA, and Protein Binding)

Comparing female infected to uninfected plants (heatmaps shown in Figure S3) across all stages did not reveal any significantly enriched categories. However, comparisons of infected female Stages 9 and 10 with their respective uninfected sample DEGs showed enrichment for the category Binding (GO:0005488), similar to male infected *vs.* uninfected plants. Additional enriched categories for infected female Stage 9 with uninfected female Stage 9 included Oxidoreductase Activity (GO:0016491), Cellular Process (GO:0009987; protein phosphatases, protein kinases), UDP-glycosyltransferase activity (GO:0035251), and Membrane (GO:0016020; calcium-dependent voltage channel, G-protein-coupled receptor). Interestingly, the female infected *vs.* uninfected Stage 9 comparison revealed enrichment for Defense Response (GO:0006952).

### Gene categories enrichment during distinct stages of male and female plant infection

Comparing across individual infected stages (*e.g.*, infected male floral stem *vs.* infected male Stages 8, 9, or 10; see Figure 1) rather than infected *vs.* uninfected stages revealed conserved categories of gene enrichment for floral stem across most categories that were also enriched in comparisons with the uninfected male floral stem samples. However, no significantly enriched categories were found in infected male floral stem, Stage 8, or Stage 9 compared with respective uninfected counterparts. In contrast, both infected male Stages 9 and 10 comparisons with infected male Stage 8 indicated enrichment for Cellular Process (GO:0009987), Catalytic Activity (GO:0003824), and Binding (GO:0005488). These categories were also observed for infected female floral stems *vs.* the corresponding Stage 8 and 9 samples (see Figure 2 and Table S8). Later stages of infection in females (*e.g.*, infected female Stages 9 or 10 *vs.* Stage 8) were not only associated with the categories of Catalytic Activity and Cellular Metabolic Process (GO:0044237) but also with examples of stress responses [Peroxidase Activity (GO:0004601); Hydrogen Peroxide Catalytic Process (GO:0042744); Response to Oxidative Stress (GO:0006979); Oxidation-Reduction Process (GO:0055114); Response to Biotic Stimulus (GO:0009607); Defense Response (GO:0006952)] as well as evidence of transposable element activities [Transposase (GO:0004803) and Transposition, DNA-mediated (GO:0006313)]. Interestingly, as seen above, the increased host expression of stress-related genes coincided with greater expression of fungal glyoxal oxidase (H_2_O_2_-producing enzymes), fungal stress response genes, and potential virulence factors, *e.g.*, pectinesterases, glycosyl hydrolase family 28, and multicopper oxidases. Additional categories of DEGs were also interesting. For example, comparing MI9 and FI9 with the corresponding uninfected stages identified several agamous-like MADS-box genes (AGL61 and AGL80, predicted to interact and be involved in male gametophyte development) (Masiero *et al.* 2011) that were not found in other comparisons.

**Figure 6:**
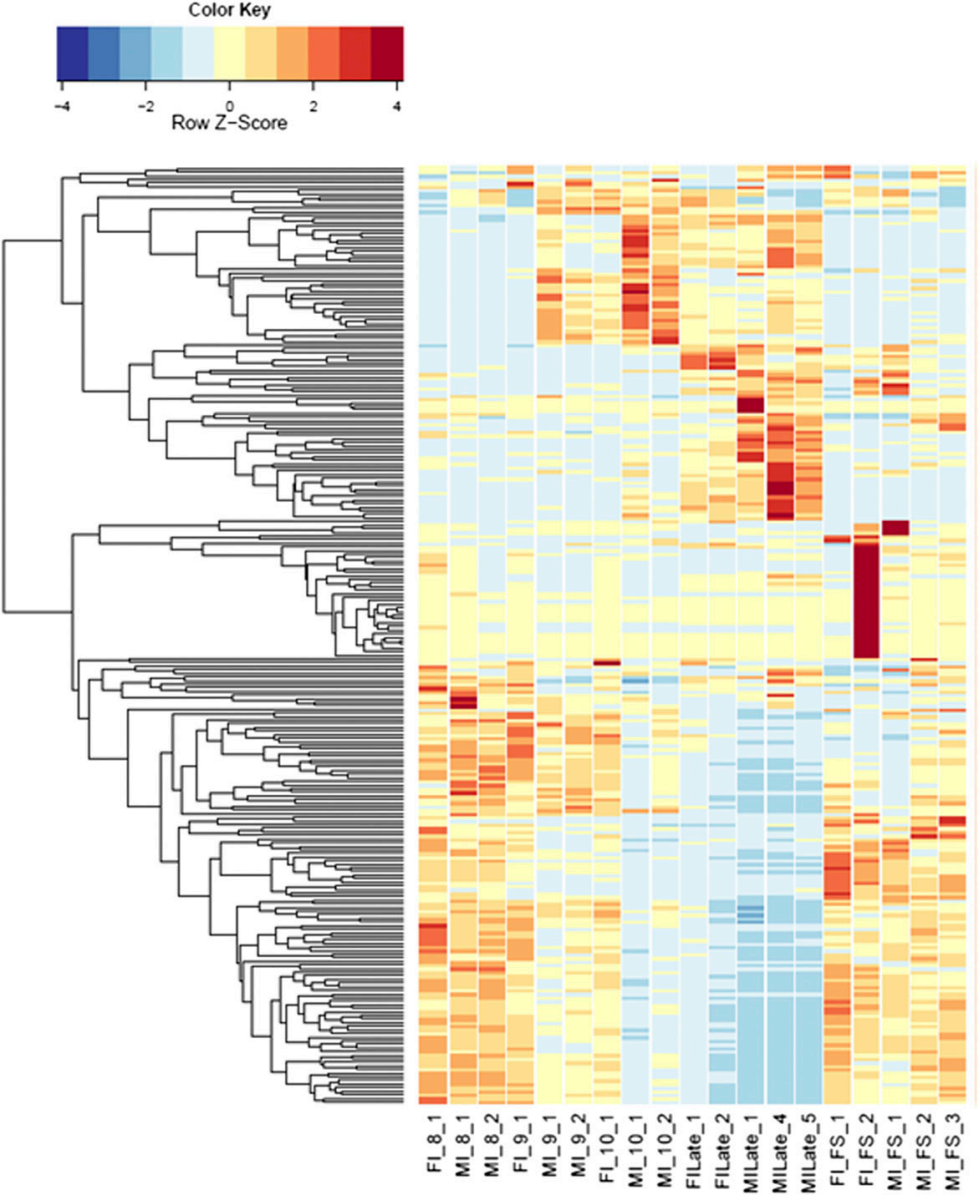
Heatmaps of host infected male *vs.* infected female comparisons at each Stage. fdr <1e−2, 6557 genes. MI or FI, infected male or female plants, respectively, compared at stages indicated.

The majority of comparisons between distinct stages of Male infected *vs.* Female infected samples (see Figure 6 for heatmap) revealed redundant categories of enrichments with previous comparisons (*e.g.*, Catalytic Activity, Single-organism Process, Transposition-related, Metabolic Process, Binding, Single-organism Metabolic Process, Ion Binding, and Transport) regardless of whether comparing earlier stages to later stages or later stages to earlier stages. Moreover, the results were reproduced for combined analyses of infected male and female stages (*e.g.*, floral stem *vs.* Stage 8). However, comparison of infected male Stage 10 *vs.* infected female Stage 10 primarily revealed enrichment of genes specifically involved in protein synthesis [*i.e.*, Structural Constituent of the Ribosome (GO:0003735), Cytosolic Small Ribosomal Subunit (GO:0022627), Cytosolic Large Ribosomal Subunit (GO:0022625), Translation (GO:0006412)].

## Discussion

This is the first transcriptome analysis of the early floral development stages of *S. latifolia* infected with *M. lychnidis-dioicae* and provides a global picture of changes in gene expression of the fungal pathogen and plant host during development of the pathogenic process. These analyses also revealed host genes that were differentially expressed in infected *vs.* uninfected males as well as for infected *vs.* uninfected females. We also examined how fungal gene expression differed between infected male and infected female host plants.

We found that many of the fungal genes differentially regulated in teliosporogenesis may be SSPs, SPs, and proteins unique to *M. lychnidis-dioicae* or to *Microbotryum* species in general. The majority of these do not have functional domains that can suggest a specific mechanism but nonetheless provide novel targets to explore this interesting process. Given that this phytopathogen system coevolves with its hosts, particularly to the extent of having a narrow host range (Le Gac *et al.* 2007a), it is not unexpected that the genes that are involved in the interaction with its host may be novel compared with other phytopathogen systems (Gowda *et al.* 2007). In fact, despite reports of local maladaptation of *Microbotryum* species (Kaltz and Shykoff 1998; Kaltz *et al.* 1999), possibly due to reduced migration of the fungus and a selfing breeding system (compared with outcrossing of the host), more recent studies using more comprehensive collections of host–pathogen samples indeed suggest a strong phylogeographic costructure for this pathosystem (Feurtey *et al.* 2016).

Enrichment of SSPs and SPs in the down-regulated genes of late-stage development suggests that these were more important in the earlier stages of codevelopment. There was almost no other enrichment found in the comparison of other stages. In the original literature examining the codevelopment of *M. lychnidis-dioicae* with *S. latifolia* (Uchida *et al.* 2003) by electron microscopy, teliospores start appearing at Stage 9, while hyphae were still present. Hence, the change may be subtle and asynchronous and therefore require more precise selection of samples and may be marked by only relatively small changes in gene expression. Such adjustment was used in a study of rice and blast fungus (Kawahara *et al.* 2012), where an FDR of 10^−3^ was used initially. For the pathogenesis-related protein PR1 to be detected as differentially expressed, an FDR of 10^−2^ was required. Since fungal cells represent only a very small portion of the total tissue in the early stages of development, compared to the amount of plant tissue, more biological replicates would facilitate detection of such small changes in gene expression.

In previous transcriptome analyses of the *M. lychnidis-dioicae–S. latifolia* interaction, Zemp *et al.* (2015) found that the majority of changes in the host transcriptome affect genes with male-biased expression in healthy plants. In females, these same genes are up-regulated. In contrast, infected males showed down-regulation of these genes, leading to a demasculinization of the transcriptome. Genes with female-biased expression in healthy plants were also affected in opposite directions in the two sexes, but to a lesser extent. These genes were overall down-regulated in females and up-regulated in males, causing a defeminization in infected females and a feminization of the transcriptome in infected males. Their results revealed strong sex-specific responses to pathogen infection in a dioecious plant (Zemp *et al.* 2015).

Of note, Zemp *et al.* (2015) (see their Table S5) identified a number of enriched and underrepresented gene categories that were up-regulated or down-regulated; these gene categories were located on both non-sex-linked and sex-linked contigs. Several of these were categories identified for the hosts in our study as well.

For example, the current study indicates that in infected females, the categories of enrichment for non-sex-linked GO terms included extracellular region (GO:0005576), transporter activity (GO:0005215), and transport (GO:0006810); however, the only sex-linked category overrepresented in the earlier study (Zemp *et al.* 2015) that was also found in our study was that of catalytic activity (GO:0003824). Under-represented categories in the current study found in common with the Zemp *et al.* study were DNA Metabolic Process (GO:0006259) and kinase activity (GO:0016301). Gene categories that were enriched in infected males in common to both studies were Response to Biotic Stimulus (GO:0009607), Extracellular Region (0005576), Kinase Activity (GO:0016301), and, interestingly, Defense Response (GO:0006952); in contrast, DNA Metabolic Process (GO:0006259) and Translation (GO:0006412) were underrepresented. The categories enriched in the group that was down-regulated were Extracellular Region (GO:0005576) and Transporter Activity (GO:0005215); as seen previously, Translation (GO:0006412) was underrepresented. However, in contrast with what was seen in the up-regulated group, Kinase activity (GO:0016301) was underrepresented here.

Surprisingly, Zemp *et al.* (2015) found that Defense-Related genes were enriched in infected males but not in the females. The authors cautioned against interpretation of the GO enrichment since defense-related genes tend to evolve quickly with the pathogen and, thus, may not be detectable using means of established databases and signature sequences. The fact that only 26.63% for non-sex-linked to 57.30% of sex-linked contigs were annotated by the GO terms attest to this caveat of the analysis. Our analyses of discrete stages of infected flowers also revealed that later stages of infection in females (*e.g.*, FI9 *vs.* FI8 and FI10 *vs.* FI8) were associated with up-regulation of gene categories associated with Stress Response, Transposable Element Activity, and also Defense Response.

Altered gene expression of pathogens and manipulation of their hosts are cornerstones of host/pathogen interactions. We have identified fungal changes in gene expression in the later stages of the pathogen lifecycle *in planta*. These include a variety of enriched gene categories that reflect the pathogen’s actions to manipulate the host via both potential effectors (SSPs) and virulence factors (*e.g.*, glycosyl hydrolase family 28, pectinesterases, glyoxal oxidase producing H_2_O_2_). The expression of these factors progressed differentially in parallel with bud development, with SSPs being down-regulated in the later bud stages (*i.e.*, infected male Stage10 *vs.* infected male Stage 8). Badouin *et al.* (2017) identified genes in *M. lychnidis-dioicae* and *M. silenes-dioicae* apparently under positive selection, as evidenced by selective sweeps. This group of genes included those predicted to encode proteins with CFEM domains (PF05730.4) known to play a role in pathogenesis in other fungal systems, as well as those with MFS (PF07690.9), sugar transporter (PF00083.17), OPT (PF03169.8), Cu-oxidase (PF07732), and aspartyl protease (PF00026) domains. In many cases in our study, these *M. lychnidis-dioicae* genes were preferentially expressed *in planta* and/or at specific bud Stages of infected male plants, although only three of the genes identified in selective sweeps were predicted to encode secreted proteins (CFEM: MVLG_00859, Cu-oxidase: MVLG_02184, and aspartyl protease: MVLG_04416). By way of comparison with another biotrophic pathogen, the rust pathogen *Melampsora larici-populina* and its interaction with poplar (Petre *et al.* 2012), a similar study identified 19 pathogen transcripts encoding early-expressed SSPs representing candidate effectors, as well as a poplar transcript encoding a sulfate transporter showing a dramatic increase upon colonization by either virulent or avirulent *M. larici-populina* strains. Similarly, a pathogen/host transcriptome analysis for the hemibiotroph blast fungus *M. oryzae* and its rice host (Kawahara *et al.* 2012) found up-regulation of 240 fungal putative secreted proteins, suggesting that these candidate fungal effector genes may play important roles in initial infection processes. As in our study, among the putative effectors were glycosyl hydrolases, but also found were cutinases and LysM domain-containing proteins; the rice host showed up-regulation of pathogenesis-related and phytoalexin biosynthetic genes. Consistent with these findings, comparisons of different fungal life strategies (Lowe *et al.* 2014) have found that host responses to two different *Leptosphaeria* species during the first 7 d of infection differed based on whether the pathogen was a necrotroph (*e.g.*, *L. biglobosa ’canadensis*’) or a hemibiotroph (*e.g.*, *L. maculans ’brassicae*’). While the necrotroph expressed more genes for cell wall degrading enzymes than the hemibiotroph, the latter expressed many genes in the Carbohydrate Binding Module class of CAZy, particularly CBM50 genes, with potential roles in the evasion of basal innate immunity in the host plant. The host response to necrotroph infection was activation of the jasmonic acid and salicylic acid defense pathways, while infection with the hemibiotroph triggered a high level of expression of isochorismate synthase 1, a reporter for salicylic acid signaling. In the context of this discussion, comparison in our study of host and pathogen gene expression in the *M. lychnidis-dioicae*/*S. latifolia* interaction during later bud Stages revealed a period of apparent mutual stress, where host stress and defense responses to fungal virulence factors were accompanied by evidence of fungal stress (*e.g.*, enrichment of PF00012.13|HSP70 and GO:0006950|Response to Stress). In parallel, the host transcriptome was altered by fungal infection and, in these critical stages of flower bud development, there is evidence that the female, the less-preferred host for this parasite, shows additional signs of stress and attempts at mounting a defense response. These results begin to provide a more complete picture of the interaction and suggests directions for future investigations of this fascinating model system.

## Supplementary Material

Supplemental material is available online at www.g3journal.org/lookup/suppl/doi:10.1534/g3.117.300318/-/DC1.
