# Supplementary figures and images for: Pas de deux: An Intricate Dance of Anther Smut and Its Host
Source: G3 (Bethesda). 2017 Dec 1;8(2):505–18. doi: 10.1534/g3.117.300318 (PMC5919739; doi:10.1534/g3.117.300318)

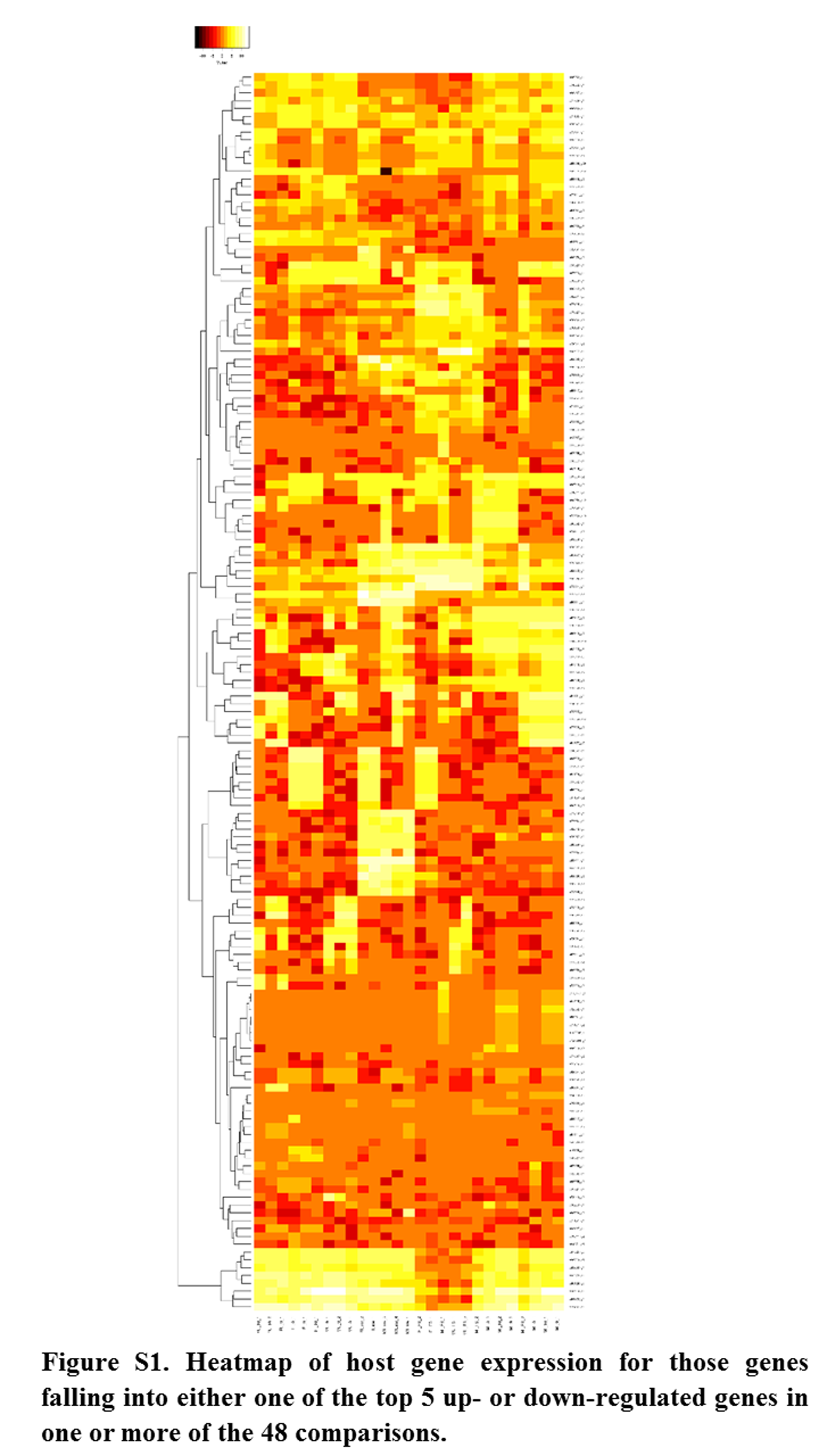

Supplement: Supplementary file 1 [file 505FigureS1.tif]

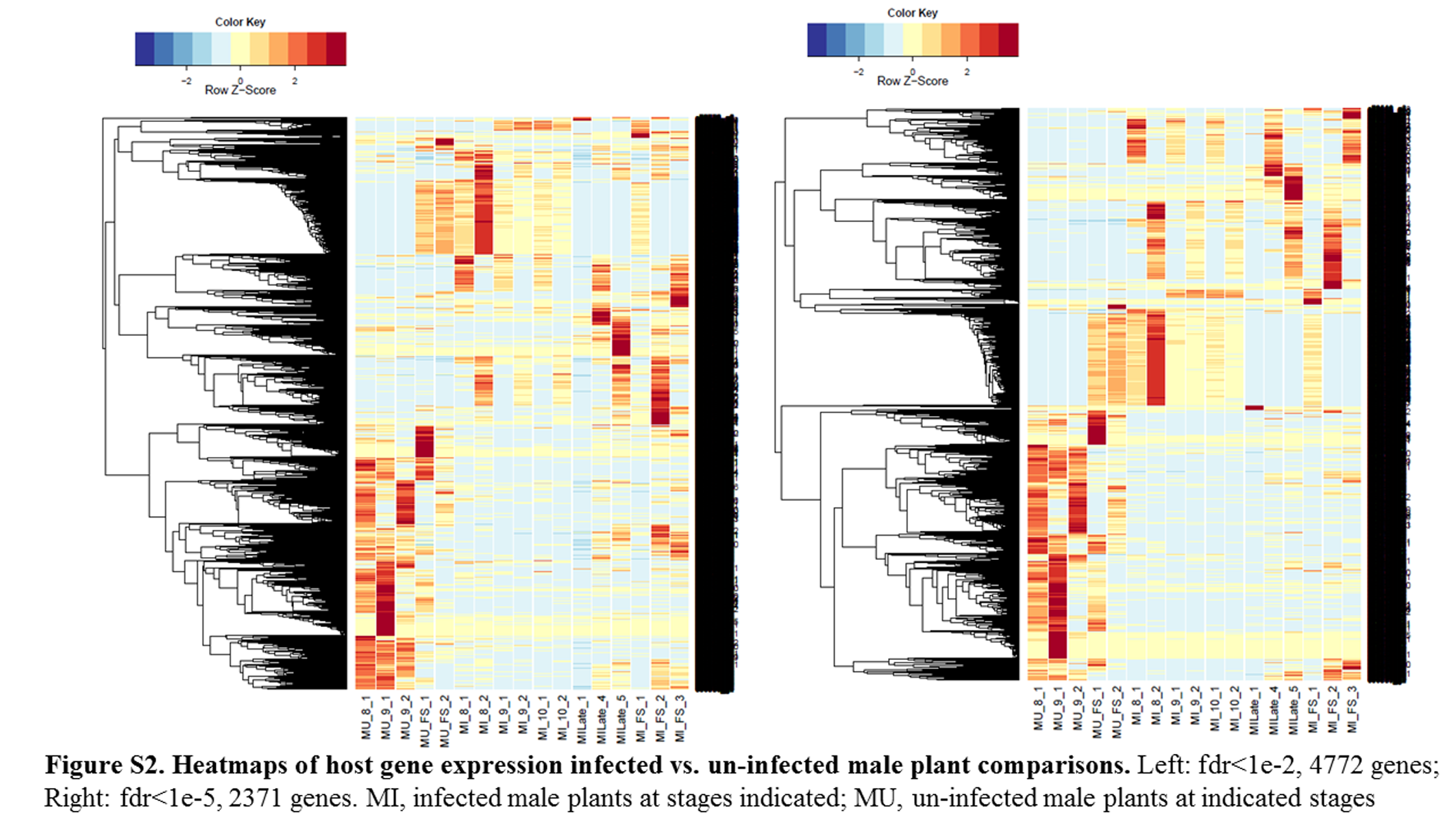

Supplement: Supplementary file 2 [file 505FigureS2.tif]

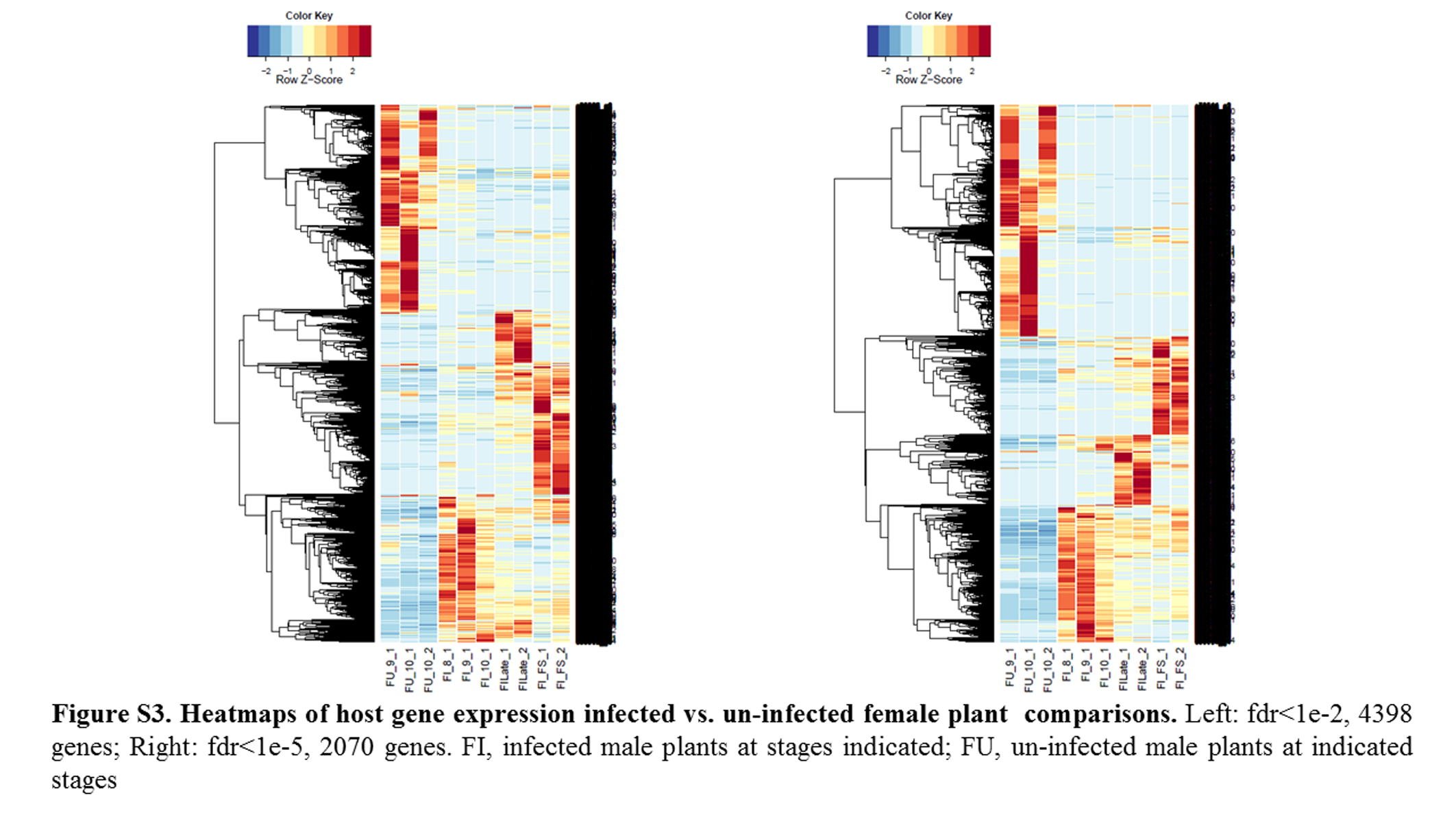

Supplement: Supplementary file 3 [file 505FigureS3.tif]

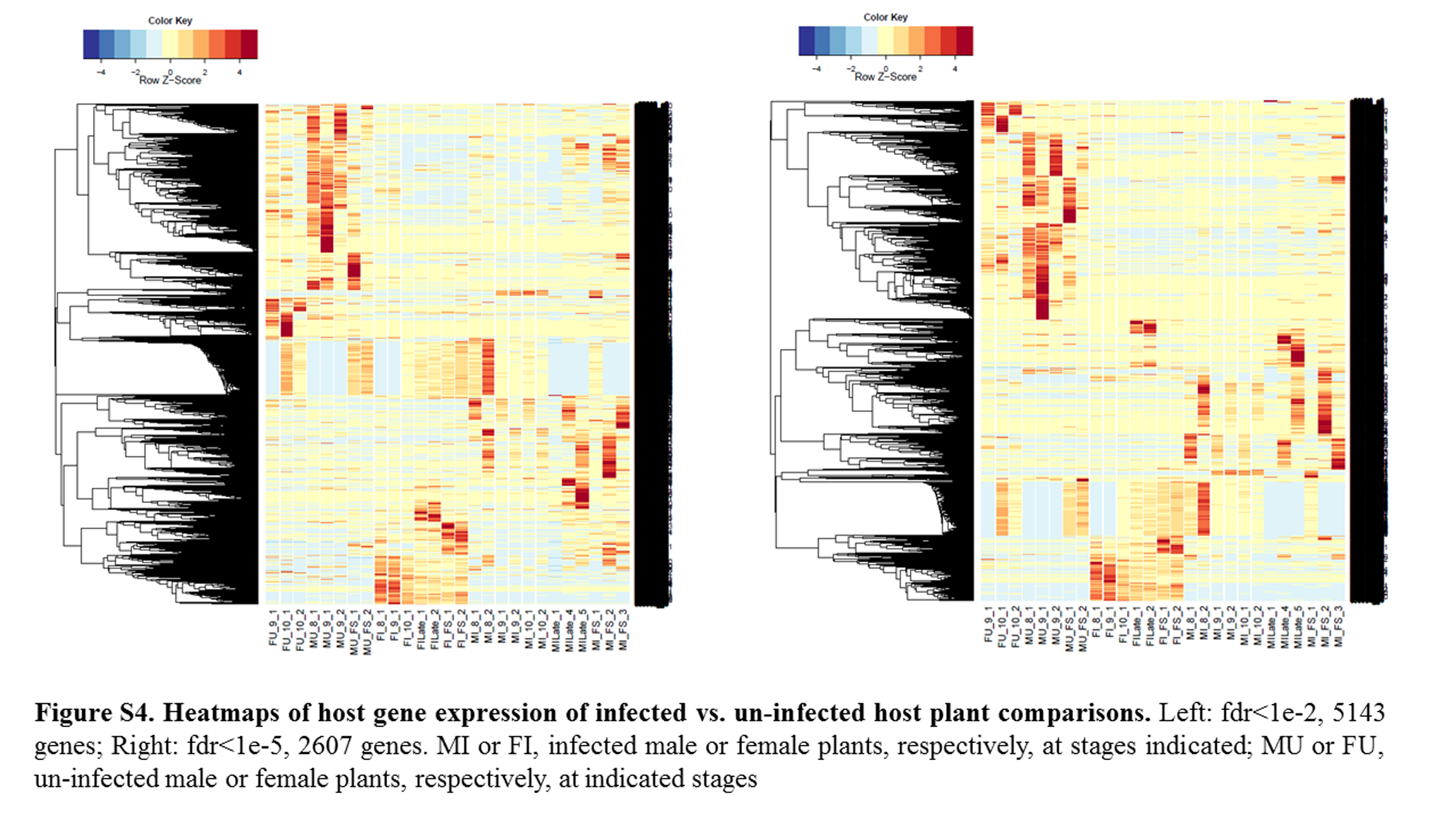

Supplement: Supplementary file 4 [file 505FigureS4.tif]

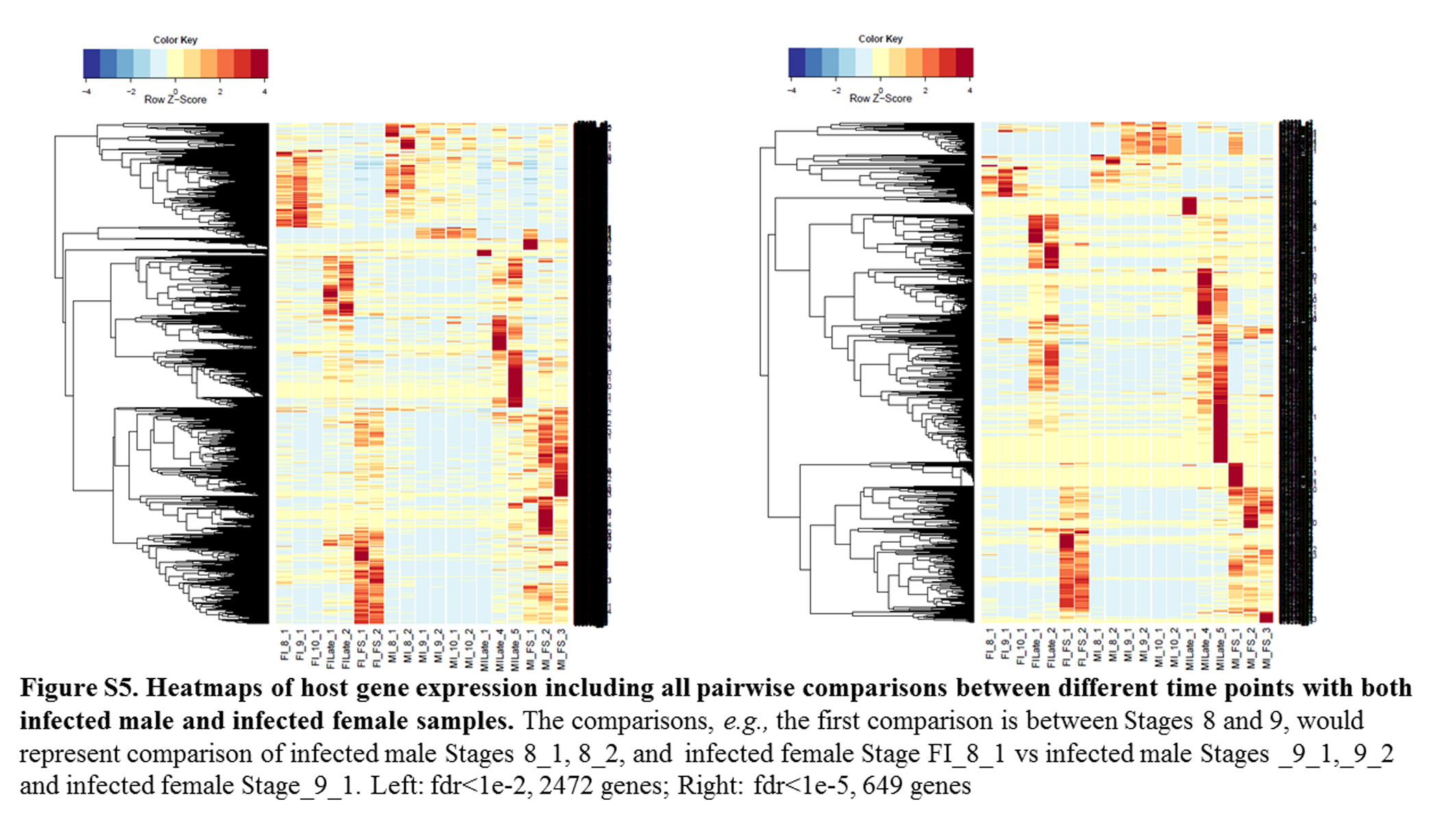

Supplement: Supplementary file 5 [file 505FigureS5.tif]

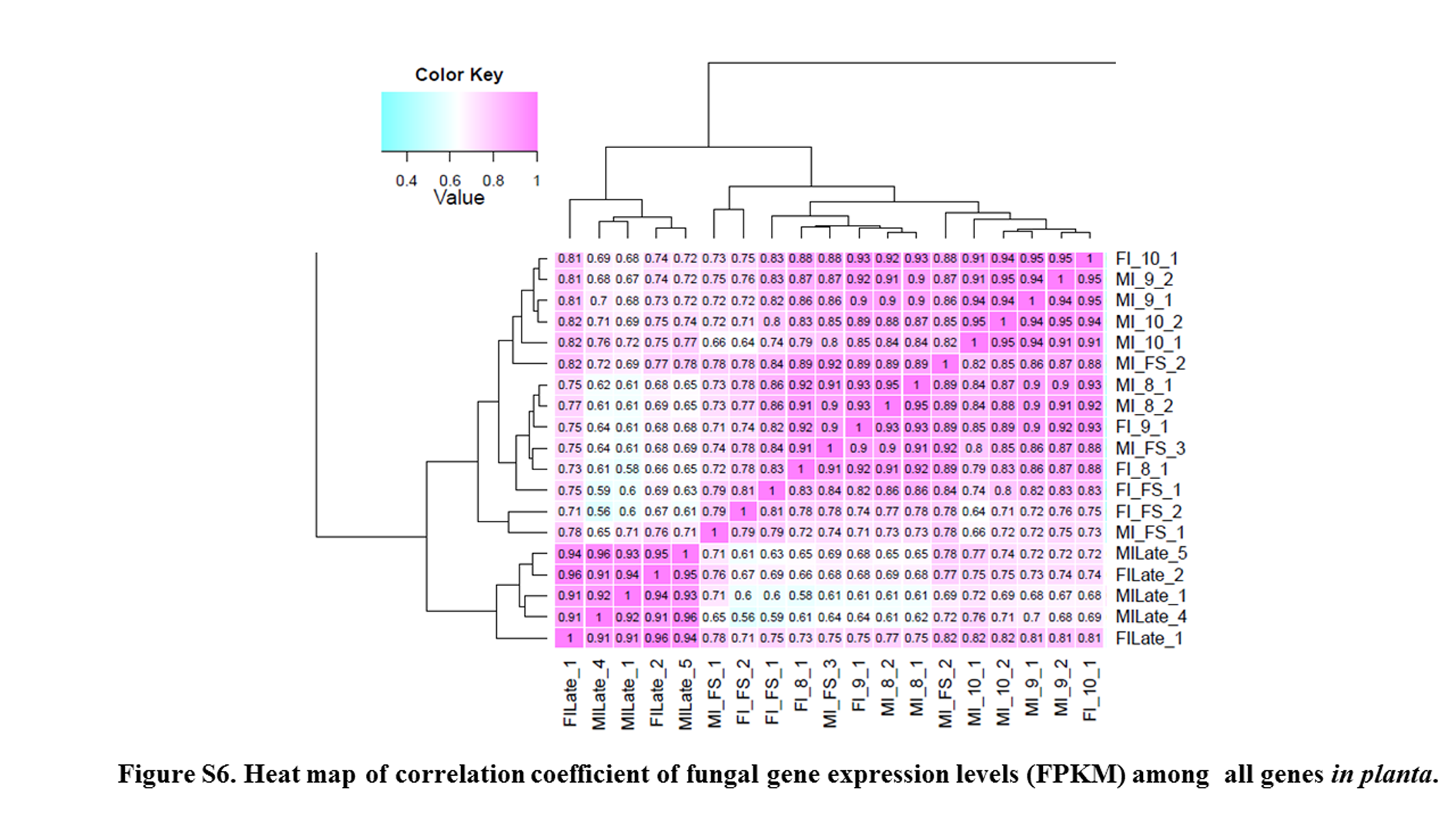

Supplement: Supplementary file 6 [file 505FigureS6.tif]

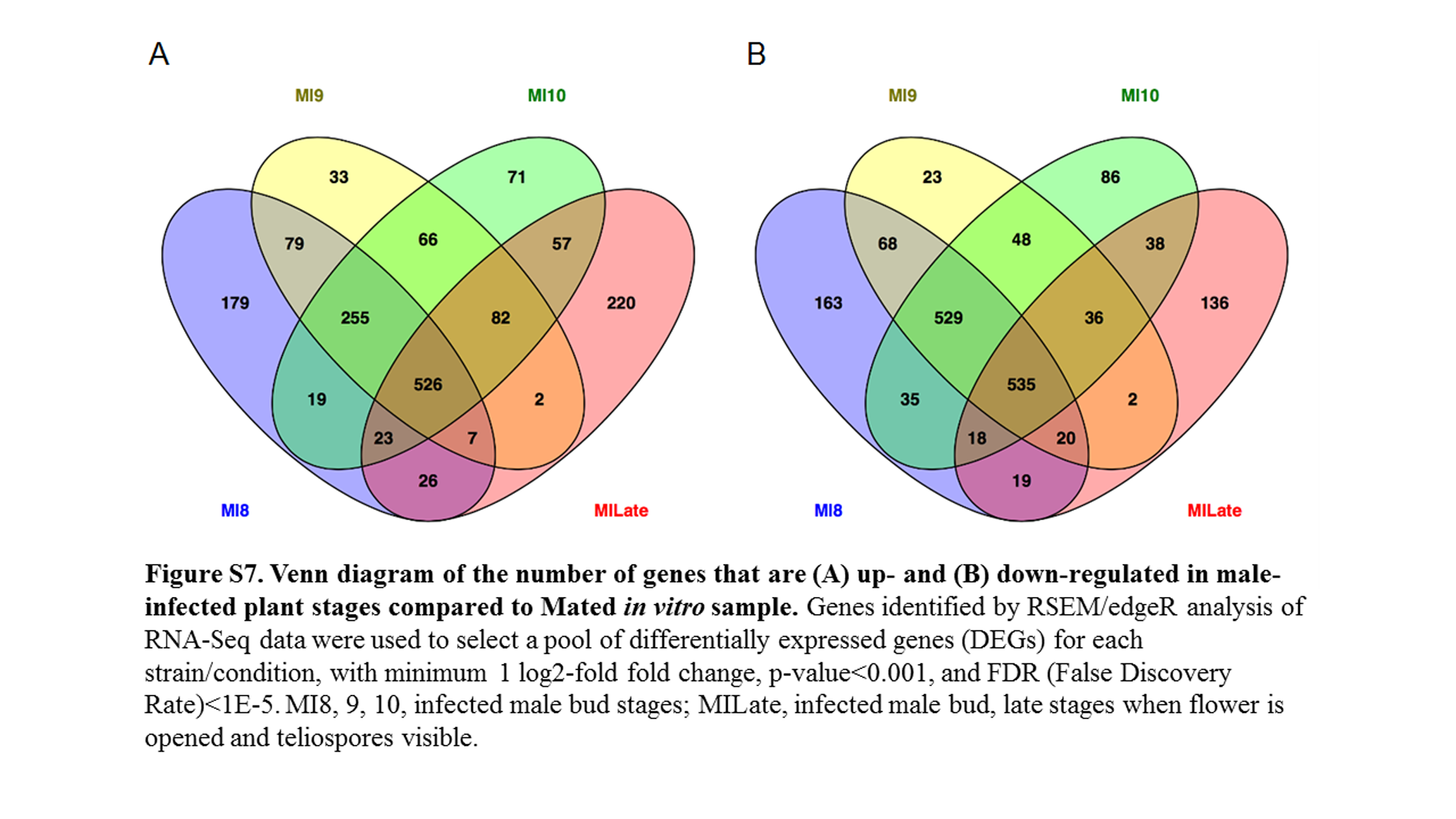

Supplement: Supplementary file 7 [file 505FigureS7.tif]

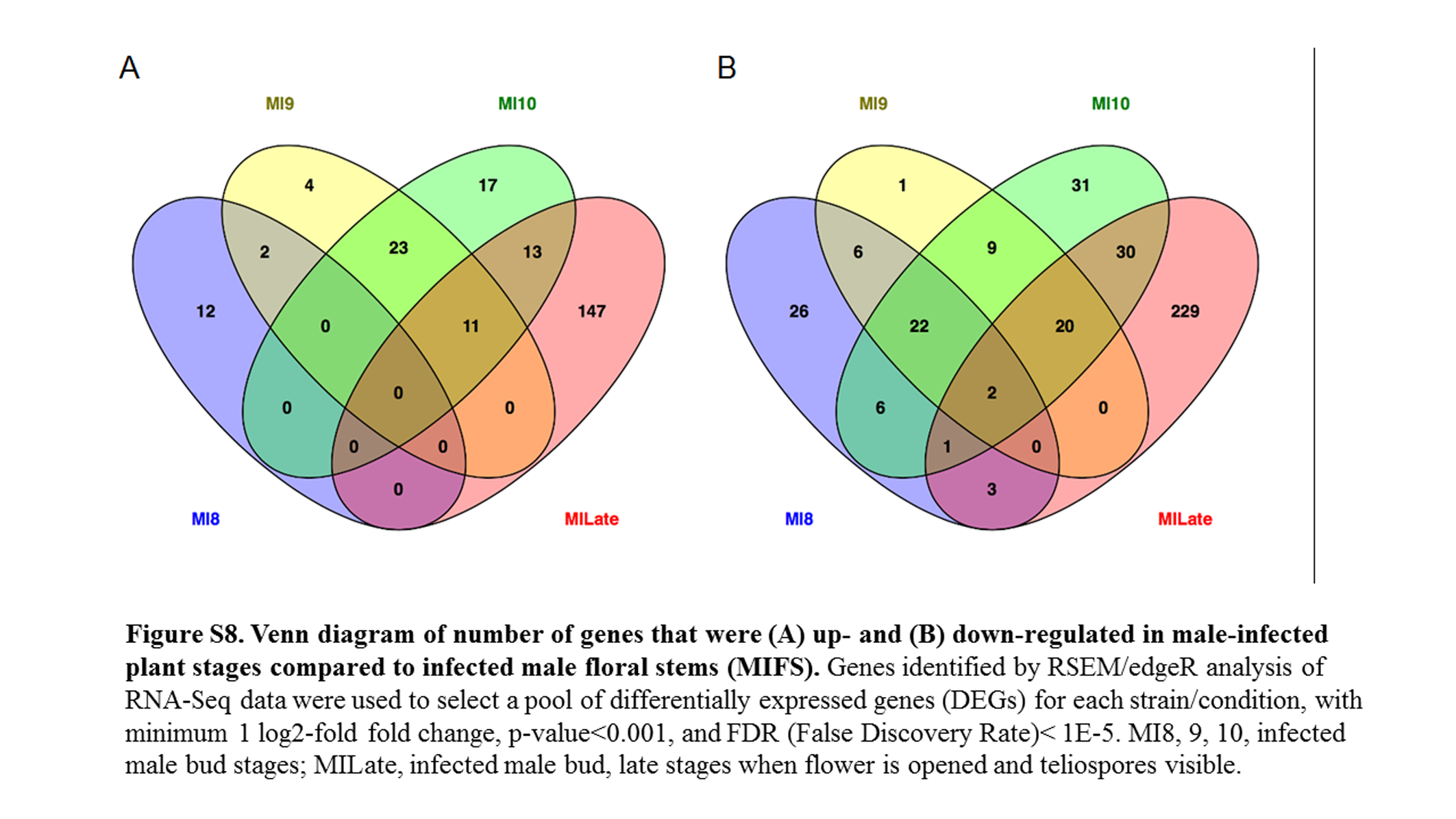

Supplement: Supplementary file 8 [file 505FigureS8.tif]

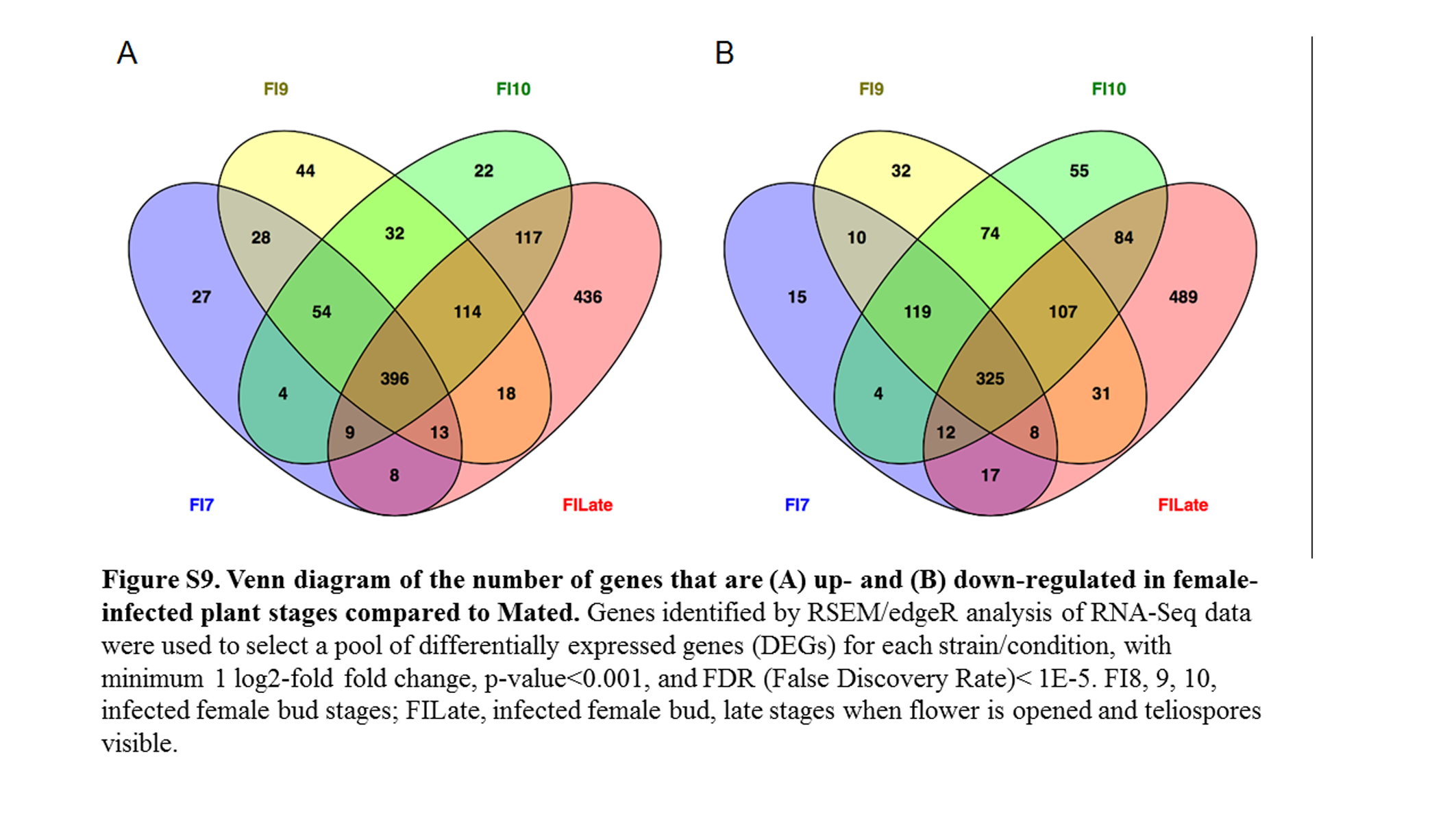

Supplement: Supplementary file 9 [file 505FigureS9.tif]

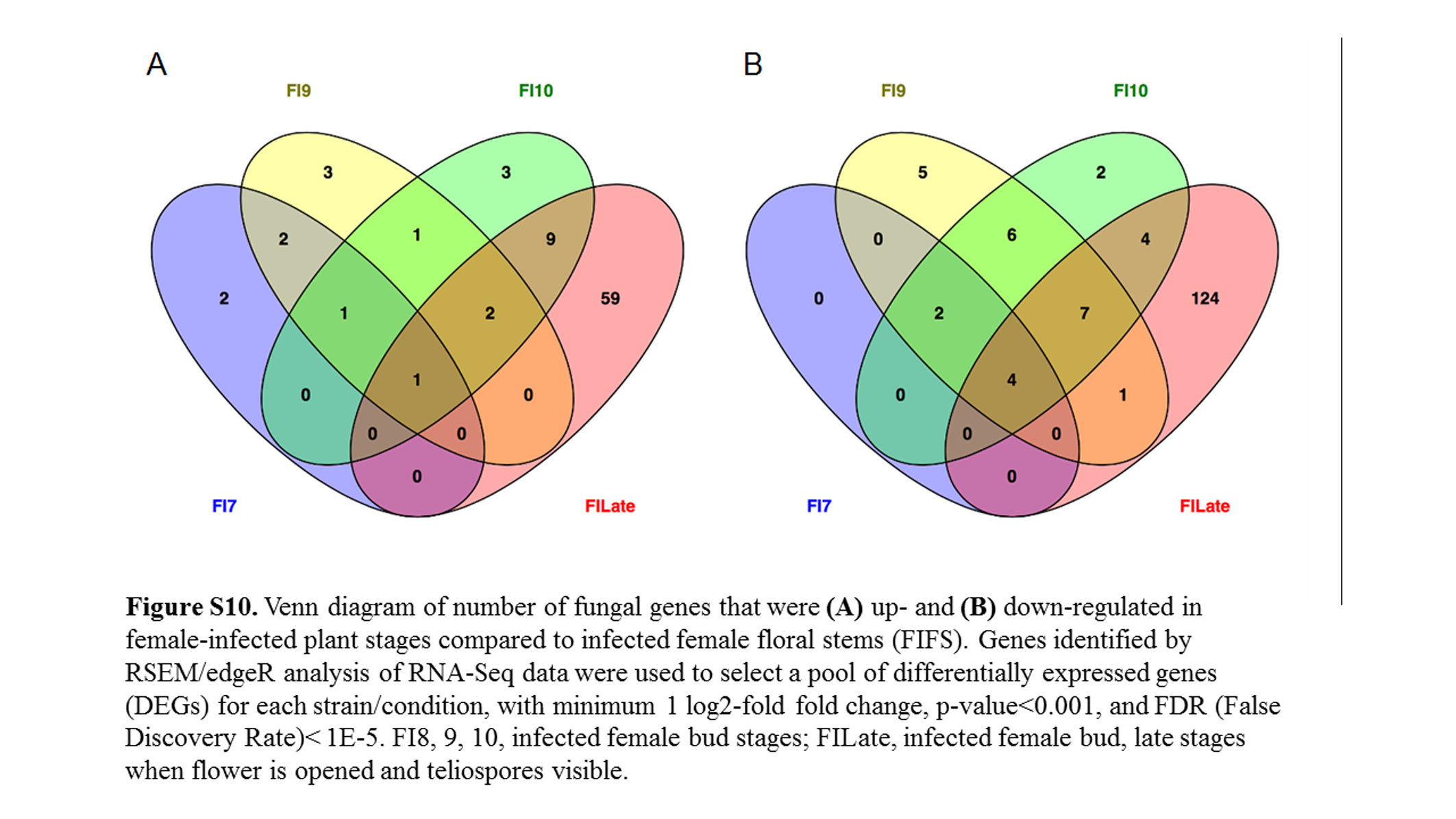

Supplement: Supplementary file 10 [file 505FigureS10.tif]

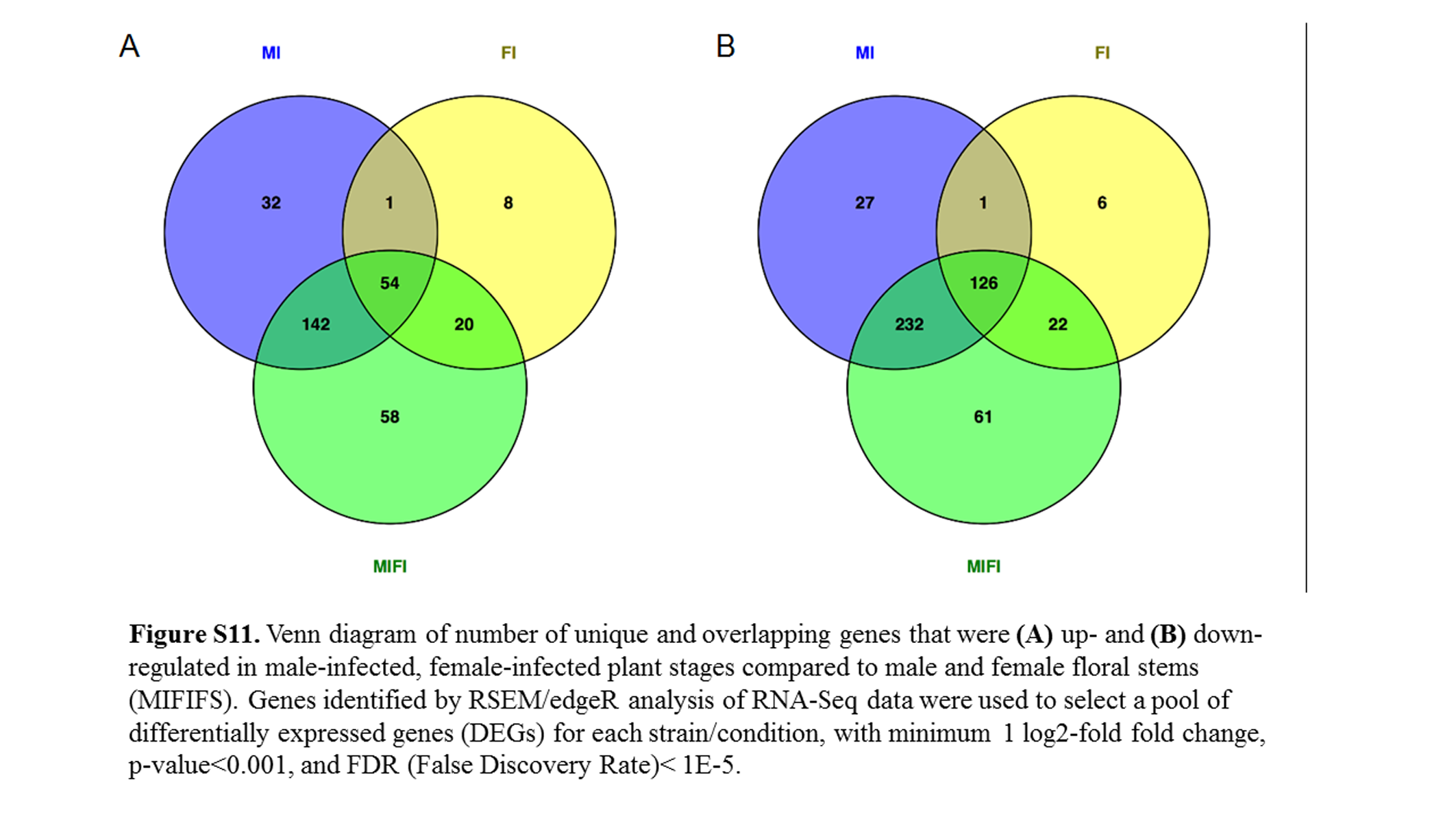

Supplement: Supplementary file 11 [file 505FigureS11.tif]

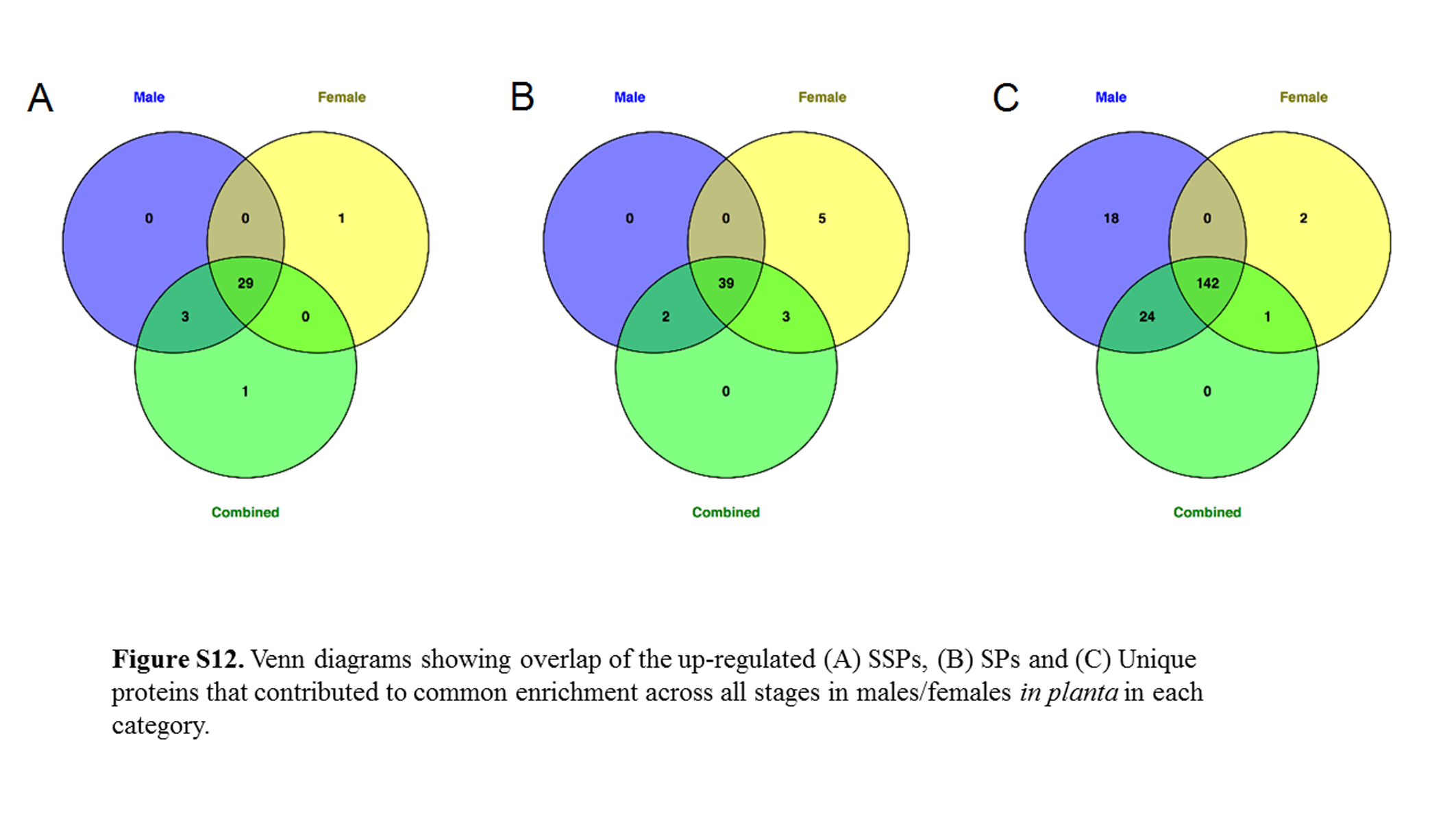

Supplement: Supplementary file 12 [file 505FigureS12.tif]

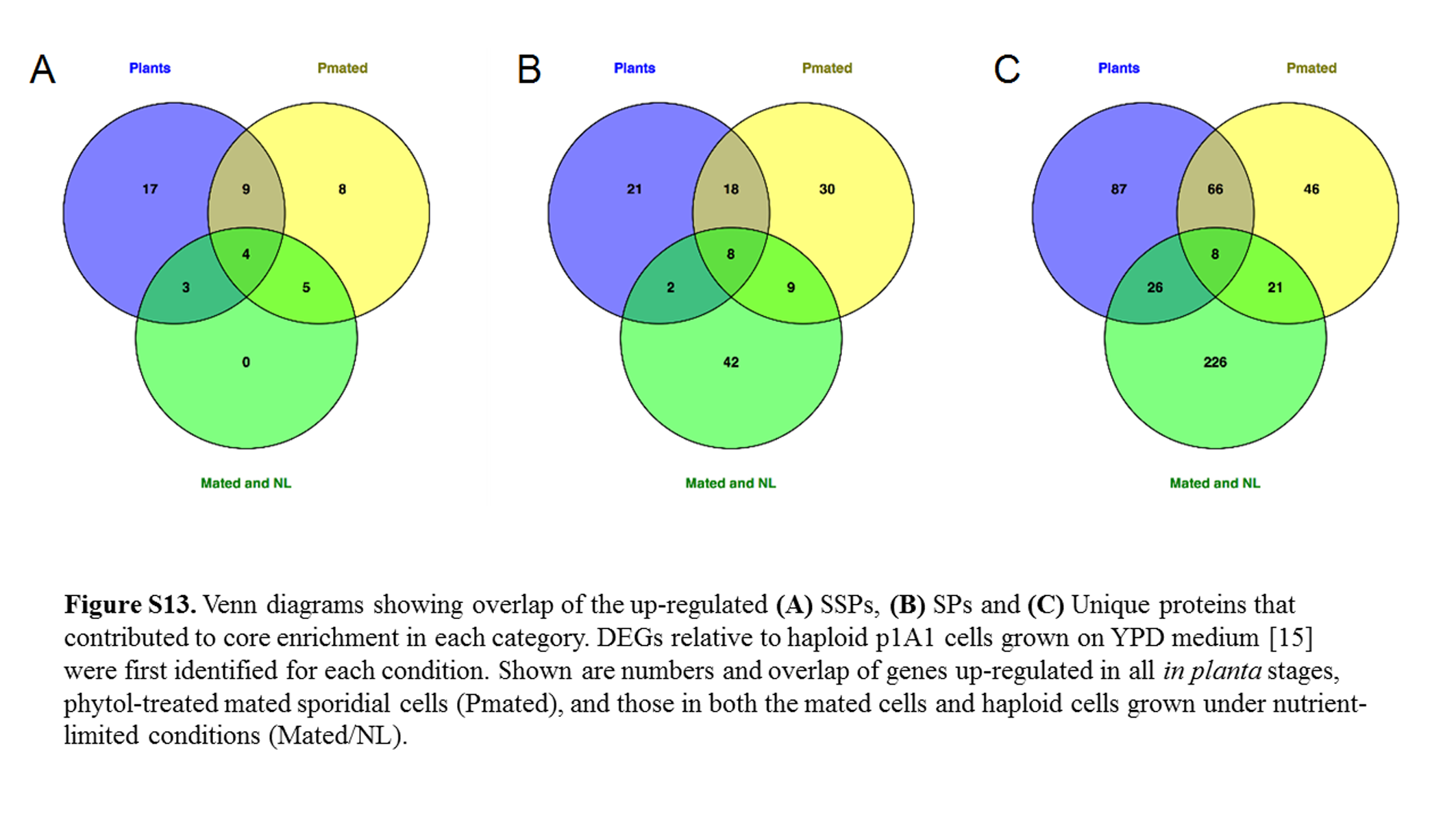

Supplement: Supplementary file 13 [file 505FigureS13.tif]

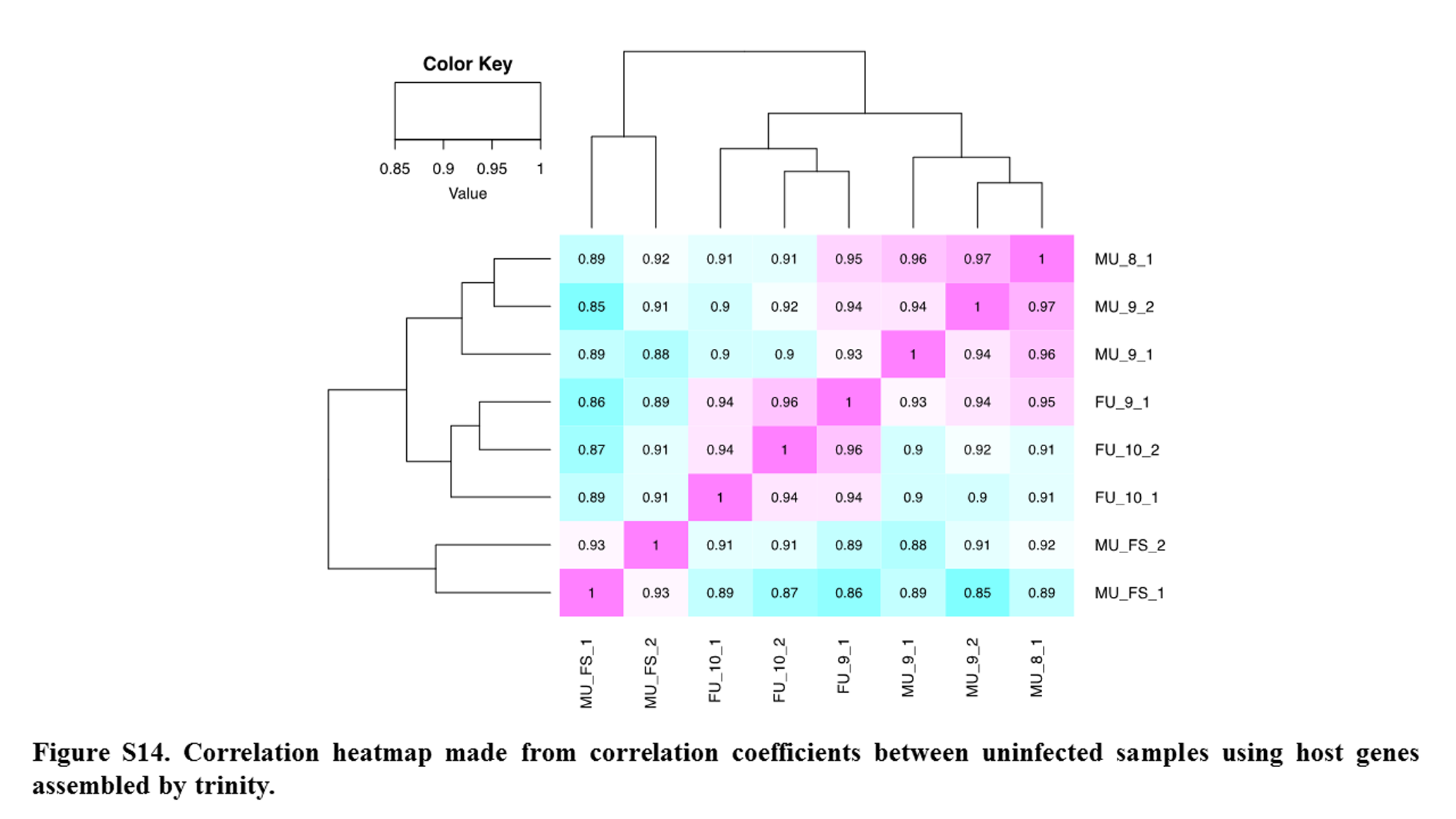

Supplement: Supplementary file 14 [file 505FigureS14.tif]
