# Supplementary material for: Pas de deux: An Intricate Dance of Anther Smut and Its Host
Source: G3 (Bethesda). 2017 Dec 1;8(2):505–18. doi: 10.1534/g3.117.300318 (PMC5919739; doi:10.1534/g3.117.300318)
Supplement: Supplementary file 15 [file 505TableS1.docx]

| **S1 Table: Differential analysis of fungal genes using edgeR with different cutoffs.** | | | | |  |
| --- | --- | --- | --- | --- | --- |
|  |  |  |  |  |  |
| **1a Table: cutoff: FDR < 1e-2** | |  |  |  |  |
|  |  |  |  |  |  |
| **# Dif_gene** | **#Up** | **#Down** | **ConditionA** | **ConditionB** | **Notes** |
| 3851 | 1725 | 2126 | Mated_1,Mated_2 | MI_8_1,MI_8_2 | Pairwise comparison of MI samples |
| 3687 | 1671 | 2016 | Mated_1,Mated_2 | MI_9_1,MI_9_2 |  |
| 3786 | 1728 | 2058 | Mated_1,Mated_2 | MI_10_1,MI_10_2 |  |
| 2606 | 1238 | 1368 | Mated_1,Mated_2 | MI_FS_1,MI_FS_2,MI_FS_3 |  |
| 3121 | 1592 | 1529 | Mated_1,Mated_2 | MILate_1,MILate_4,MILate_5 |  |
| 351 | 231 | 120 | MI_8_1,MI_8_2 | MI_9_1,MI_9_2 |  |
| 942 | 449 | 493 | MI_8_1,MI_8_2 | MI_10_1,MI_10_2 |  |
| 265 | 216 | 49 | MI_8_1,MI_8_2 | MI_FS_1,MI_FS_2,MI_FS_3 |  |
| 2221 | 1281 | 940 | MI_8_1,MI_8_2 | MILate_1,MILate_4,MILate_5 |  |
| 62 | 25 | 37 | MI_9_1,MI_9_2 | MI_10_1,MI_10_2 |  |
| 321 | 200 | 121 | MI_9_1,MI_9_2 | MI_FS_1,MI_FS_2,MI_FS_3 |  |
| 1776 | 1067 | 709 | MI_9_1,MI_9_2 | MILate_1,MILate_4,MILate_5 |  |
| 591 | 382 | 209 | MI_10_1,MI_10_2 | MI_FS_1,MI_FS_2,MI_FS_3 |  |
| 1682 | 1080 | 602 | MI_10_1,MI_10_2 | MILate_1,MILate_4,MILate_5 |  |
| 1144 | 581 | 563 | MI_FS_1,MI_FS_2,MI_FS_3 | MILate_1,MILate_4,MILate_5 |  |
| 2198 | 1008 | 1190 | Mated_1,Mated_2 | FI_8_1 | Pairwise comparison of FI samples |
| 2641 | 1248 | 1393 | Mated_1,Mated_2 | FI_9_1 |  |
| 2881 | 1349 | 1532 | Mated_1,Mated_2 | FI_10_1 |  |
| 1349 | 617 | 732 | Mated_1,Mated_2 | FI_FS_1,FI_FS_2 |  |
| 3585 | 1734 | 1851 | Mated_1,Mated_2 | FILate_1,FILate_2 |  |
| 16 | 14 | 2 | FI_8_1 | FI_9_1 |  |
| 74 | 47 | 27 | FI_8_1 | FI_10_1 |  |
| 60 | 42 | 18 | FI_8_1 | FI_FS_1,FI_FS_2 |  |
| 953 | 505 | 448 | FI_8_1 | FILate_1,FILate_2 |  |
| 9 | 7 | 2 | FI_9_1 | FI_10_1 |  |
| 159 | 95 | 64 | FI_9_1 | FI_FS_1,FI_FS_2 |  |
| 1167 | 609 | 558 | FI_9_1 | FILate_1,FILate_2 |  |
| 179 | 120 | 59 | FI_10_1 | FI_FS_1,FI_FS_2 |  |
| 980 | 629 | 351 | FI_10_1 | FILate_1,FILate_2 |  |
| 537 | 239 | 298 | FI_FS_1,FI_FS_2 | FILate_1,FILate_2 |  |
| 3808 | 1743 | 2065 | Mated_1,Mated_2 | MI_8_1,MI_8_2,FI_8_1 | Pairwise comparison of MI and FI samples: treat MI and corresponding FI samples as replicates |
| 3578 | 1594 | 1984 | Mated_1,Mated_2 | MI_9_1,MI_9_2,FI_9_1 |  |
| 3632 | 1652 | 1980 | Mated_1,Mated_2 | MI_10_1,MI_10_2,FI_10_1 |  |
| 2274 | 1038 | 1236 | Mated_1,Mated_2 | MI_FS_1,MI_FS_2,MI_FS_3,FI_FS_1,FI_FS_2 |  |
| 3162 | 1535 | 1627 | Mated_1,Mated_2 | MILate_1,MILate_4,MILate_5,FILate_1,FILate_2 |  |
| 207 | 158 | 49 | MI_8_1,MI_8_2,FI_8_1 | MI_9_1,MI_9_2,FI_9_1 |  |
| 626 | 317 | 309 | MI_8_1,MI_8_2,FI_8_1 | MI_10_1,MI_10_2,FI_10_1 |  |
| 254 | 216 | 38 | MI_8_1,MI_8_2,FI_8_1 | MI_FS_1,MI_FS_2,MI_FS_3,FI_FS_1,FI_FS_2 |  |
| 2347 | 1311 | 1036 | MI_8_1,MI_8_2,FI_8_1 | MILate_1,MILate_4,MILate_5,FILate_1,FILate_2 |  |
| 29 | 17 | 12 | MI_9_1,MI_9_2,FI_9_1 | MI_10_1,MI_10_2,FI_10_1 |  |
| 365 | 243 | 122 | MI_9_1,MI_9_2,FI_9_1 | MI_FS_1,MI_FS_2,MI_FS_3,FI_FS_1,FI_FS_2 |  |
| 2021 | 1207 | 814 | MI_9_1,MI_9_2,FI_9_1 | MILate_1,MILate_4,MILate_5,FILate_1,FILate_2 |  |
| 546 | 350 | 196 | MI_10_1,MI_10_2,FI_10_1 | MI_FS_1,MI_FS_2,MI_FS_3,FI_FS_1,FI_FS_2 |  |
| 1928 | 1186 | 742 | MI_10_1,MI_10_2,FI_10_1 | MILate_1,MILate_4,MILate_5,FILate_1,FILate_2 |  |
| 1326 | 685 | 641 | MI_FS_1,MI_FS_2,MI_FS_3,FI_FS_1,FI_FS_2 | MILate_1,MILate_4,MILate_5,FILate_1,FILate_2 |  |
| 14 | 1 | 13 | FI_8_1 | MI_8_1,MI_8_2 | MI versus FI samples |
| 70 | 38 | 32 | FI_9_1 | MI_9_1,MI_9_2 |  |
| 72 | 25 | 47 | FI_10_1 | MI_10_1,MI_10_2 |  |
| 26 | 3 | 23 | FI_FS_1,FI_FS_2 | MI_FS_1,MI_FS_2,MI_FS_3 |  |
| 163 | 53 | 110 | FILate_1,FILate_2 | MILate_1,MILate_4,MILate_5 |  |
| 2014 | 1168 | 846 | Mated_1,Mated_2 | Pmated12_1,Pmated12_2,Pmated12_3,Pmated12_4 | Pairwise comparison of Pmated samples |
| 2343 | 1376 | 967 | Mated_1,Mated_2 | Pmated24_1,Pmated24_2,Pmated24_3 |  |
| 886 | 744 | 142 | Mated_1,Mated_2 | Pmated48_1,Pmated48_2 |  |
| 794 | 436 | 358 | Pmated12_1,Pmated12_2,Pmated12_3,Pmated12_4 | Pmated24_1,Pmated24_2,Pmated24_3 |  |
| 1093 | 615 | 478 | Pmated12_1,Pmated12_2,Pmated12_3,Pmated12_4 | Pmated48_1,Pmated48_2 |  |
| 473 | 269 | 204 | Pmated24_1,Pmated24_2,Pmated24_3 | Pmated48_1,Pmated48_2 |  |
|  |  |  |  |  |  |
|  |  |  |  |  |  |
|  |  |  |  |  |  |
| **1b Table: cutoff: FDR < 1e-5** | |  |  |  |  |
|  |  |  |  |  |  |
| **# Dif_gene** | **#Up** | **#Down** | **ConditionA** | **ConditionB** | **Notes** |
| 2501 | 1114 | 1387 | Mated_1,Mated_2 | MI_8_1,MI_8_2 | Pairwise comparison of MI samples |
| 2311 | 1050 | 1261 | Mated_1,Mated_2 | MI_9_1,MI_9_2 |  |
| 2424 | 1099 | 1325 | Mated_1,Mated_2 | MI_10_1,MI_10_2 |  |
| 1275 | 639 | 636 | Mated_1,Mated_2 | MI_FS_1,MI_FS_2,MI_FS_3 |  |
| 1747 | 943 | 804 | Mated_1,Mated_2 | MILate_1,MILate_4,MILate_5 |  |
| 139 | 104 | 35 | MI_8_1,MI_8_2 | MI_9_1,MI_9_2 |  |
| 364 | 193 | 171 | MI_8_1,MI_8_2 | MI_10_1,MI_10_2 |  |
| 80 | 66 | 14 | MI_8_1,MI_8_2 | MI_FS_1,MI_FS_2,MI_FS_3 |  |
| 1177 | 706 | 471 | MI_8_1,MI_8_2 | MILate_1,MILate_4,MILate_5 |  |
| 6 | 2 | 4 | MI_9_1,MI_9_2 | MI_10_1,MI_10_2 |  |
| 100 | 60 | 40 | MI_9_1,MI_9_2 | MI_FS_1,MI_FS_2,MI_FS_3 |  |
| 838 | 513 | 325 | MI_9_1,MI_9_2 | MILate_1,MILate_4,MILate_5 |  |
| 185 | 121 | 64 | MI_10_1,MI_10_2 | MI_FS_1,MI_FS_2,MI_FS_3 |  |
| 843 | 598 | 245 | MI_10_1,MI_10_2 | MILate_1,MILate_4,MILate_5 |  |
| 456 | 171 | 285 | MI_FS_1,MI_FS_2,MI_FS_3 | MILate_1,MILate_4,MILate_5 |  |
| 1049 | 539 | 510 | Mated_1,Mated_2 | FI_8_1 | Pairwise comparison of FI samples |
| 1405 | 699 | 706 | Mated_1,Mated_2 | FI_9_1 |  |
| 1528 | 748 | 780 | Mated_1,Mated_2 | FI_10_1 |  |
| 476 | 298 | 178 | Mated_1,Mated_2 | FI_FS_1,FI_FS_2 |  |
| 2184 | 1111 | 1073 | Mated_1,Mated_2 | FILate_1,FILate_2 |  |
| 2 | 2 | 0 | FI_8_1 | FI_9_1 |  |
| 17 | 13 | 4 | FI_8_1 | FI_10_1 |  |
| 12 | 6 | 6 | FI_8_1 | FI_FS_1,FI_FS_2 |  |
| 334 | 159 | 175 | FI_8_1 | FILate_1,FILate_2 |  |
| 2 | 1 | 1 | FI_9_1 | FI_10_1 |  |
| 35 | 25 | 10 | FI_9_1 | FI_FS_1,FI_FS_2 |  |
| 432 | 230 | 202 | FI_9_1 | FILate_1,FILate_2 |  |
| 42 | 25 | 17 | FI_10_1 | FI_FS_1,FI_FS_2 |  |
| 350 | 262 | 88 | FI_10_1 | FILate_1,FILate_2 |  |
| 211 | 71 | 140 | FI_FS_1,FI_FS_2 | FILate_1,FILate_2 |  |
| 2446 | 1088 | 1358 | Mated_1,Mated_2 | MI_8_1,MI_8_2,FI_8_1 | Pairwise comparison of MI and FI samples: treat MI and corresponding FI samples as replicates |
| 2189 | 955 | 1234 | Mated_1,Mated_2 | MI_9_1,MI_9_2,FI_9_1 |  |
| 2270 | 994 | 1276 | Mated_1,Mated_2 | MI_10_1,MI_10_2,FI_10_1 |  |
| 1028 | 488 | 540 | Mated_1,Mated_2 | MI_FS_1,MI_FS_2,MI_FS_3,FI_FS_1,FI_FS_2 |  |
| 1800 | 898 | 902 | Mated_1,Mated_2 | MILate_1,MILate_4,MILate_5,FILate_1,FILate_2 |  |
| 90 | 75 | 15 | MI_8_1,MI_8_2,FI_8_1 | MI_9_1,MI_9_2,FI_9_1 |  |
| 224 | 130 | 94 | MI_8_1,MI_8_2,FI_8_1 | MI_10_1,MI_10_2,FI_10_1 |  |
| 94 | 77 | 17 | MI_8_1,MI_8_2,FI_8_1 | MI_FS_1,MI_FS_2,MI_FS_3,FI_FS_1,FI_FS_2 |  |
| 1286 | 764 | 522 | MI_8_1,MI_8_2,FI_8_1 | MILate_1,MILate_4,MILate_5,FILate_1,FILate_2 |  |
| 0 | 0 | 0 | MI_9_1,MI_9_2,FI_9_1 | MI_10_1,MI_10_2,FI_10_1 |  |
| 145 | 98 | 47 | MI_9_1,MI_9_2,FI_9_1 | MI_FS_1,MI_FS_2,MI_FS_3,FI_FS_1,FI_FS_2 |  |
| 1048 | 648 | 400 | MI_9_1,MI_9_2,FI_9_1 | MILate_1,MILate_4,MILate_5,FILate_1,FILate_2 |  |
| 227 | 153 | 74 | MI_10_1,MI_10_2,FI_10_1 | MI_FS_1,MI_FS_2,MI_FS_3,FI_FS_1,FI_FS_2 |  |
| 969 | 652 | 317 | MI_10_1,MI_10_2,FI_10_1 | MILate_1,MILate_4,MILate_5,FILate_1,FILate_2 |  |
| 545 | 218 | 327 | MI_FS_1,MI_FS_2,MI_FS_3,FI_FS_1,FI_FS_2 | MILate_1,MILate_4,MILate_5,FILate_1,FILate_2 |  |
| 2 | 0 | 2 | FI_8_1 | MI_8_1,MI_8_2 | MI versus FI samples |
| 19 | 9 | 10 | FI_9_1 | MI_9_1,MI_9_2 |  |
| 16 | 2 | 14 | FI_10_1 | MI_10_1,MI_10_2 |  |
| 7 | 0 | 7 | FI_FS_1,FI_FS_2 | MI_FS_1,MI_FS_2,MI_FS_3 |  |
| 57 | 4 | 53 | FILate_1,FILate_2 | MILate_1,MILate_4,MILate_5 |  |
| 924 | 605 | 319 | Mated_1,Mated_2 | Pmated12_1,Pmated12_2,Pmated12_3,Pmated12_4 | Pairwise comparison of Pmated samples |
| 1068 | 761 | 307 | Mated_1,Mated_2 | Pmated24_1,Pmated24_2,Pmated24_3 |  |
| 404 | 386 | 18 | Mated_1,Mated_2 | Pmated48_1,Pmated48_2 |  |
| 239 | 156 | 83 | Pmated12_1,Pmated12_2,Pmated12_3,Pmated12_4 | Pmated24_1,Pmated24_2,Pmated24_3 |  |
| 427 | 262 | 165 | Pmated12_1,Pmated12_2,Pmated12_3,Pmated12_4 | Pmated48_1,Pmated48_2 |  |
| 173 | 129 | 44 | Pmated24_1,Pmated24_2,Pmated24_3 | Pmated48_1,Pmated48_2 |  |
