# Supplementary material for: Pas de deux: An Intricate Dance of Anther Smut and Its Host
Source: G3 (Bethesda). 2017 Dec 1;8(2):505–18. doi: 10.1534/g3.117.300318 (PMC5919739; doi:10.1534/g3.117.300318)
Supplement: Supplementary file 17 [file 505TableS3.docx]

**S3 Table: Number of significant DEGs and GO terms for each of the 48 comparisons^a^**

| COMPARISON | SIG DEGs | DEGs UP | DEGs DN | SIG GO |
| --- | --- | --- | --- | --- |
| FI10_vs_FI8 | 1402 | 776 | 626 | 14 |
| FI10_vs_FI9 | 773 | 605 | 168 | 12 |
| FI10_vs_FU10 | 3737 | 1733 | 2004 | 28 |
| FI9_vs_FI8 | 648 | 175 | 473 | 26 |
| FI9_vs_FU9 | 3005 | 1386 | 1619 | 25 |
| FI9andFI10_vs_FU9_10 | 2362 | 1020 | 1342 | 12 |
| FILate_vs_FI_10 | 3341 | 2259 | 1082 | 64 |
| FILate_vs_FI_8 | 6060 | 3444 | 2616 | 103 |
| FILate_vs_FI_9 | 6961 | 4077 | 2884 | 138 |
| FILate_vs_FI_FS | 4384 | 1971 | 2413 | 156 |
| FI_FS_vs_FI10 | 5930 | 3298 | 2632 | 161 |
| FI_FS_vs_FI8 | 7819 | 4200 | 3619 | 186 |
| FI_FS_vs_FI9 | 9032 | 4899 | 4133 | 240 |
| FI_vs_FU | 3819 | 2495 | 1324 | 92 |
| MI10_vs_FI10 | 3994 | 2612 | 1382 | 28 |
| MI10_vs_MI8 | 135 | 121 | 14 | 5 |
| MI8_9_FS_Late_vs_MU8_9_FS | 2584 | 1354 | 1230 | 38 |
| MI8_9_FS_vs_MU8_9_FS | 2163 | 1033 | 1130 | 20 |
| MI8_vs_FI8 | 5725 | 3016 | 2709 | 51 |
| MI8_vs_MU8 | 5019 | 2404 | 2615 | 54 |
| MI9_vs_FI9 | 4779 | 3031 | 1748 | 35 |
| MI9_vs_MI8 | 79 | 74 | 5 | 5 |
| MI9_vs_MU9 | 1850 | 1004 | 846 | 14 |
| MILate_vs_FILate | 3041 | 1840 | 1201 | 31 |
| MILate_vs_MI10 | 1200 | 733 | 467 | 12 |
| MILate_vs_MI8 | 2014 | 1132 | 882 | 34 |
| MILate_vs_MI9 | 1469 | 919 | 550 | 17 |
| MILate_vs_MI_FS | 1314 | 651 | 663 | 15 |
| MI_FS_vs_FI_FS | 2526 | 1797 | 729 | 16 |
| MI_FS_vs_MI10 | 947 | 714 | 233 | 31 |
| MI_FS_vs_MI8 | 1017 | 793 | 224 | 26 |
| MI_FS_vs_MI9 | 1131 | 855 | 276 | 41 |
| MI_FS_vs_MU_FS | 1303 | 710 | 593 | 12 |
| MIandFI10_vs_MIandFI8 | 93 | 75 | 18 | 3 |
| MIandFI8_vs_MU8 | 4800 | 2621 | 2179 | 61 |
| MIandFI9_vs_MIandFI8 | 50 | 42 | 8 | 1 |
| MIandFI9_vs_MUandFU9 | 1697 | 856 | 841 | 6 |
| MIandFI_FS_vs_MIandFI10 | 1851 | 1255 | 596 | 61 |
| MIandFI_FS_vs_MIandFI8 | 1711 | 1134 | 577 | 50 |
| MIandFI_FS_vs_MIandFI9 | 2245 | 1489 | 756 | 75 |
| MIandFI_FS_vs_MU_FS | 1332 | 760 | 572 | 15 |
| MIandFI_Late_vs_MIandFI10 | 1028 | 867 | 161 | 22 |
| MIandFI_Late_vs_MIandFI8 | 1378 | 1062 | 316 | 29 |
| MIandFI_Late_vs_MIandFI9 | 1408 | 1095 | 313 | 32 |
| MIandFI_Late_vs_MIandFI_FS | 1638 | 836 | 802 | 39 |
| MU_vs_FU | 1355 | 1315 | 40 | 1 |
| MU_vs_FU_ALL | 784 | 741 | 43 | 1 |
| MaleAndFemaleInfected10_vs_FU_10 | 1471 | 943 | 528 | 6 |

**^a^MU, uninfected male plants; MI, infected male plants; FU, uninfected female plants; FI, infected female plants; FS, floral stem; all other designations refer to Stage of bud/infection**
