# Supplementary material for: Pas de deux: An Intricate Dance of Anther Smut and Its Host
Source: G3 (Bethesda). 2017 Dec 1;8(2):505–18. doi: 10.1534/g3.117.300318 (PMC5919739; doi:10.1534/g3.117.300318)
Supplement: Supplementary file 18 [file 505TableS4.docx]

**S4 Table. Summary of short read alignment for each sample.**

| **#Aligned** | **notAligned** | **Total** | **Sample** | **Condition** | **Comments** |
| --- | --- | --- | --- | --- | --- |
| 40621982 | 33275742 | 73897724 | Mated_1 | **Mated** | **Control** |
| 41131650 | 36355546 | 77487196 | Mated_2 |  |  |
| 3297450 | 101317336 | 104614786 | FILate_1 | **FILate** | **Group 2** |
| 3195010 | 92134174 | 95329184 | FILate_2 |  |  |
| 1788336 | 163026580 | 164814916 | FI_10_1 | **FI_10** |  |
| 727668 | 133541794 | 134269462 | FI_8_1 | **FI_8** |  |
| 1342140 | 146773300 | 148115440 | FI_9_1 | **FI_9** |  |
| 154798 | 105189786 | 105344584 | FI_FS_1 | **FI_FS** |  |
| 269766 | 220063574 | 220333340 | FI_FS_2 |  |  |
| 3287036 | 17067412 | 20354448 | MILate_1 | **MILate** | **Group 1** |
| 2728 | 40156 | 42884 | MILate_2 |  | **MI_8_2 resequenced; MILate_2 and MILate_3 have too few reads, so are not considered for differential analysis.** |
| 3174 | 4772 | 7946 | MILate_3 |  |  |
| 27096698 | 130232314 | 157329012 | MILate_4 |  |  |
| 14045118 | 129839436 | 143884554 | MILate_5 |  |  |
| 3295936 | 78868662 | 82164598 | MI_10_1 | **MI_10** |  |
| 1454148 | 75929160 | 77383308 | MI_10_2 |  |  |
| 1515280 | 197476518 | 198991798 | MI_8_1 | **MI_8** |  |
| 1547360 | 194306130 | 195853490 | MI_8_2 |  |  |
| 1156310 | 71078722 | 72235032 | MI_9_1 | **MI_9** |  |
| 1191216 | 74413530 | 75604746 | MI_9_2 |  |  |
| 126134 | 69791962 | 69918096 | MI_FS_1 | **MI_FS** |  |
| 1708442 | 168083730 | 169792172 | MI_FS_2 |  |  |
| 960882 | 152314994 | 153275876 | MI_FS_3 |  |  |
| 1458 | 182272890 | 182274348 | FU_10_1 | **FU_10** | **Group 6** |
| 31742 | 151436580 | 151468322 | FU_10_2 |  | **FU_10_1 resequenced** |
| 24292 | 147570490 | 147594782 | FU_9_1 | **FU_9** |  |
| 26778 | 167272886 | 167299664 | MU_8_1 | **MU_8** | **Group 5** |
| 43236 | 163948496 | 163991732 | MU_9_1 | **MU_9** |  |
| 23338 | 145030596 | 145053934 | MU_9_2 |  |  |
| 25880 | 153425382 | 153451262 | MU_FS_1 | **MU_FS** |  |
| 30168 | 150955128 | 150985296 | MU_FS_2 |  |  |
| 84562052 | 158515528 | 243077580 | Pmated12_1 | **Pmated12** | **Group 4** |
| 96416772 | 53390238 | 149807010 | Pmated12_2 |  |  |
| 94263206 | 72474252 | 166737458 | Pmated12_3 |  |  |
| 90325072 | 60837114 | 151162186 | Pmated12_4 |  |  |
| 105389760 | 56089666 | 161479426 | Pmated24_1 | **Pmated24** |  |
| 93571252 | 62652670 | 156223922 | Pmated24_2 |  |  |
| 101636554 | 62992850 | 164629404 | Pmated24_3 |  |  |
| 48205838 | 86564702 | 134770540 | Pmated48_1 | **Pmated48** |  |
| 97584258 | 55742436 | 153326694 | Pmated48_2 |  |  |
| 33033712 | 32475920 | 65509632 | p1A1_Rich_1 | **p1A1_Rich** | **Group 3** |
| 48403986 | 34053456 | 82457442 | p1A1_Rich_2 |  |  |
| 32620978 | 41601730 | 74222708 | p1A1_Water_1 | **p1A1_Water** |  |
| 30398154 | 38395200 | 68793354 | p1A1_Water_2 |  |  |
| 46446076 | 31180474 | 77626550 | p1A2_Rich_1 | **p1A2_Rich** |  |
| 47043664 | 27131526 | 74175190 | p1A2_Rich_2 |  |  |
| 35043900 | 34483412 | 69527312 | p1A2_Water_1 | **p1A2_Water** |  |
| 35620784 | 39034036 | 74654820 | p1A2_Water_2 |  |  |
|  |  | **Total reads: 5871345160** | |  |  |
|  |  | **Reads for trinity: 3651728182 (in grey)** | |  |  |
