# Supplementary material for: Pas de deux: An Intricate Dance of Anther Smut and Its Host
Source: G3 (Bethesda). 2017 Dec 1;8(2):505–18. doi: 10.1534/g3.117.300318 (PMC5919739; doi:10.1534/g3.117.300318)
Supplement: Supplementary file 20 [file 505TableS6.docx]

S6 Table. FPKM normalized counts of three copper oxidases detected to be up-regulated in male but not in female, to illustrate the importance of biological replicates.

| **Sample_ID** | **MVLG_01868** | **MVLG_02184** | **MVLG_03092** |
| --- | --- | --- | --- |
| Mated_1^a^ | 2.2 | 0 | 0.47 |
| Mated_2^a^ | 3.04 | 0 | 0.49 |
| MIFS_1 | 47.81 | 0* | 0* |
| MIFS_2 | 83.33 | 161.01 | 36.54 |
| MIFS_3 | 40.28 | 119.58 | 44.74 |
| MI8_1 | 35.48 | 172.71 | 13.17 |
| MI8_2 | 36.02 | 127.45 | 12.44 |
| MI9_1 | 29.22 | 197.18 | 13.43 |
| MI9_2 | 26.28 | 148.89 | 4.6 |
| MI10_1 | 30.21 | 303.95 | 7.37 |
| MI10_2 | 22.31 | 246.64 | 6.89 |
| MILate_1 | 55.25 | 63.43 | 219.36 |
| MILate_4 | 84.87 | 181.65 | 791.23 |
| MILate_5 | 43.22 | 181.41 | 451.16 |
| FIFS_1 | 70.86 | 45.76 | 11.89 |
| FIFS_2 | 70.69 | 17.13 | 10.05 |
| FI8_1 | 72.21 | 191.48 | 31.89 |
| FI9_1 | 45.89 | 204.57 | 20.2 |
| FI10_1 | 38.95 | 250.5 | 13.22 |
| FILate_1 | 73.17 | 352.89 | 343.05 |
| FILate_2 | 63.49 | 200.59 | 277.05 |

* Probably outliers

^a^ Subdesignation, *e.g.,* _1 or _2, denotes the replicate number
