# Supplementary material for: Pas de deux: An Intricate Dance of Anther Smut and Its Host
Source: G3 (Bethesda). 2017 Dec 1;8(2):505–18. doi: 10.1534/g3.117.300318 (PMC5919739; doi:10.1534/g3.117.300318)
Supplement: Supplementary file 22 [file 505TableS8.docx]

S8 Table. **GO enrichments**

MUvFU

None

FI91vFu91

| GO_ID | GO_DESC | UNIV_COUNT | DEG_COUNT | OBS_to_EXP | PVal |
| --- | --- | --- | --- | --- | --- |
| GO:0016020 | membrane | 1886 | 214 | 1.514441 | 6.14E-10 |
| GO:0005488 | binding | 791 | 100 | 1.687344 | 1.38E-07 |
| GO:0016491 | oxidoreductase activity | 506 | 67 | 1.767276 | 3.48E-06 |
| GO:0016709 | oxidoreductase activity, acting on paired donors, with incorporation or reduction of molecular oxygen, NADH or NADPH as one donor, and incorporation of one atom of oxygen | 93 | 21 | 3.013815 | 3.96E-06 |
| GO:0009987 | cellular process | 299 | 45 | 2.00873 | 5.50E-06 |
| GO:0035251 | UDP-glucosyltransferase activity | 30 | 11 | 4.893861 | 5.78E-06 |
| GO:0006952 | defense response | 111 | 23 | 2.765573 | 6.58E-06 |
| GO:0044550 | secondary metabolite biosynthetic process | 95 | 20 | 2.809872 | 2.02E-05 |
| GO:0000272 | polysaccharide catabolic process | 21 | 8 | 5.084531 | 8.17E-05 |
| GO:0009812 | flavonoid metabolic process | 27 | 9 | 4.448964 | 9.84E-05 |
| GO:0009718 | anthocyanin biosynthetic process | 12 | 6 | 6.673447 | 0.000109 |
| GO:0043170 | macromolecule metabolic process | 62 | 14 | 3.013815 | 0.000157 |
| GO:0005665 | DNA-directed RNA polymerase II, core complex | 23 | 8 | 4.642398 | 0.000172 |
| GO:0016161 | beta-amylase activity | 13 | 6 | 6.160105 | 0.00019 |
| GO:0004497 | monooxygenase activity | 155 | 25 | 2.152725 | 0.000216 |
| GO:0030598 | rRNA N-glycosylase activity | 9 | 5 | 7.414941 | 0.000229 |
| GO:0008152 | metabolic process | 555 | 64 | 1.539101 | 0.000365 |
| GO:0009407 | toxin catabolic process | 20 | 7 | 4.671413 | 0.000424 |
| GO:0008061 | chitin binding | 26 | 8 | 4.106736 | 0.000448 |
| GO:0016301 | kinase activity | 308 | 40 | 1.733363 | 0.000462 |
| GO:0030247 | polysaccharide binding | 94 | 17 | 2.4138 | 0.000561 |
| GO:0004568 | chitinase activity | 28 | 8 | 3.813398 | 0.000778 |
| GO:0006032 | chitin catabolic process | 28 | 8 | 3.813398 | 0.000778 |
| GO:0055114 | oxidation-reduction process | 1464 | 141 | 1.285459 | 0.000998 |

FIFSvFI8

| GO_ID | GO_DESC | UNIV_COUNT | DEG_COUNT | OBS_to_EXP | PVal |
| --- | --- | --- | --- | --- | --- |
| GO:0003824 | catalytic activity | 384 | 62 | 2.446277 | 2.09E-11 |
| GO:0044237 | cellular metabolic process | 75 | 17 | 3.434257 | 5.86E-06 |
| GO:0044710 | single-organism metabolic process | 89 | 18 | 3.064274 | 1.70E-05 |
| GO:0044699 | single-organism process | 123 | 21 | 2.586779 | 5.19E-05 |
| GO:0006796 | phosphate-containing compound metabolic process | 28 | 8 | 4.328895 | 0.000332 |

FIFSvFI9

| GO_ID | GO_DESC | UNIV_COUNT | DEG_COUNT | OBS_to_EXP | PVal |
| --- | --- | --- | --- | --- | --- |
| GO:0003824 | catalytic activity | 384 | 60 | 2.228878 | 3.27E-09 |
| GO:0017070 | U6 snRNA binding | 7 | 5 | 10.18916 | 3.13E-05 |
| GO:0044237 | cellular metabolic process | 75 | 16 | 3.043162 | 5.12E-05 |
| GO:0044710 | single-organism metabolic process | 89 | 17 | 2.724741 | 0.00013 |
| GO:0044699 | single-organism process | 123 | 20 | 2.319483 | 0.000344 |
| GO:0006796 | phosphate-containing compound metabolic process | 28 | 8 | 4.075663 | 0.000499 |
| GO:0005665 | DNA-directed RNA polymerase II, core complex | 23 | 7 | 4.341468 | 0.000739 |

FIFSvFI10

| GO_ID | GO_DESC | UNIV_COUNT | DEG_COUNT | OBS_to_EXP | PVal |
| --- | --- | --- | --- | --- | --- |
| GO:0005665 | DNA-directed RNA polymerase II, core complex | 23 | 6 | 5.454683 | 0.000589 |

FIvFU

None

FI9vFI8

| GO_ID | GO_DESC | UNIV_COUNT | DEG_COUNT | OBS_to_EXP | PVal |
| --- | --- | --- | --- | --- | --- |
| GO:0004601 | peroxidase activity | 137 | 24 | 5.422243 | 2.11E-10 |
| GO:0042744 | hydrogen peroxide catabolic process | 93 | 22 | 7.321972 | 2.29E-10 |
| GO:0098869 | | 147 | 25 | 5.263941 | 6.01E-10 |
| GO:0005576 | extracellular region | 248 | 31 | 3.868997 | 7.10E-10 |
| GO:0006979 | response to oxidative stress | 130 | 25 | 5.952302 | 7.22E-10 |
| GO:0020037 | heme binding | 342 | 42 | 3.801119 | 9.17E-10 |
| GO:0055114 | oxidation-reduction process | 1464 | 90 | 1.902785 | 2.72E-09 |
| GO:0009607 | response to biotic stimulus | 27 | 7 | 8.024585 | 1.79E-05 |
| GO:0052886 | 9,9Prime-dicis-carotene:quinone oxidoreductase activity | 13 | 5 | 11.9046 | 3.59E-05 |
| GO:0052887 | 7,9,9Prime-tricis-neurosporene:quinone oxidoreductase activity | 13 | 5 | 11.9046 | 3.59E-05 |
| GO:0052889 | 9,9Prime-di-cis-zeta-carotene desaturation to 7,9,7Prime,9Prime-tetra-cis-lycopene | 13 | 5 | 11.9046 | 3.59E-05 |
| GO:0006952 | defense response | 111 | 13 | 3.625006 | 6.05E-05 |
| GO:0006833 | water transport | 16 | 5 | 9.672491 | 0.000112 |
| GO:0015250 | water channel activity | 17 | 5 | 9.103521 | 0.000155 |
| GO:0009813 | flavonoid biosynthetic process | 79 | 10 | 3.917971 | 0.000223 |
| GO:0009664 | plant-type cell wall organization | 54 | 8 | 4.585477 | 0.000317 |
| GO:0052696 | flavonoid glucuronidation | 68 | 9 | 4.096585 | 0.000325 |
| GO:0048046 | apoplast | 104 | 11 | 3.273766 | 0.000537 |
| GO:0003700 | sequence-specific DNA binding transcription factor activity | 273 | 20 | 2.267544 | 0.000593 |
| GO:0080043 | quercetin 3-O-glucosyltransferase activity | 60 | 8 | 4.12693 | 0.000659 |
| GO:0080044 | quercetin 7-O-glucosyltransferase activity | 60 | 8 | 4.12693 | 0.000659 |
| GO:0043565 | sequence-specific DNA binding | 159 | 14 | 2.725331 | 0.000661 |
| GO:0016702 | oxidoreductase activity, acting on single donors with incorporation of molecular oxygen, incorporation of two atoms of oxygen | 23 | 5 | 6.72869 | 0.000718 |
| GO:0005215 | transporter activity | 183 | 15 | 2.537047 | 0.000907 |
| GO:0016788 | hydrolase activity, acting on ester bonds | 94 | 10 | 3.292763 | 0.000908 |

FI9vFU9

| GO_ID | GO_DESC | UNIV_COUNT | DEG_COUNT | OBS_to_EXP | PVal |
| --- | --- | --- | --- | --- | --- |
| GO:0016020 | membrane | 1886 | 214 | 1.514441 | 6.14E-10 |
| GO:0005488 | binding | 791 | 100 | 1.687344 | 1.38E-07 |
| GO:0016491 | oxidoreductase activity | 506 | 67 | 1.767276 | 3.48E-06 |
| GO:0016709 | oxidoreductase activity, acting on paired donors, with incorporation or reduction of molecular oxygen, NADH or NADPH as one donor, and incorporation of one atom of oxygen | 93 | 21 | 3.013815 | 3.96E-06 |
| GO:0009987 | cellular process | 299 | 45 | 2.00873 | 5.50E-06 |
| GO:0035251 | UDP-glucosyltransferase activity | 30 | 11 | 4.893861 | 5.78E-06 |
| GO:0006952 | defense response | 111 | 23 | 2.765573 | 6.58E-06 |
| GO:0044550 | secondary metabolite biosynthetic process | 95 | 20 | 2.809872 | 2.02E-05 |
| GO:0000272 | polysaccharide catabolic process | 21 | 8 | 5.084531 | 8.17E-05 |
| GO:0009812 | flavonoid metabolic process | 27 | 9 | 4.448964 | 9.84E-05 |
| GO:0009718 | anthocyanin biosynthetic process | 12 | 6 | 6.673447 | 0.000109 |
| GO:0043170 | macromolecule metabolic process | 62 | 14 | 3.013815 | 0.000157 |
| GO:0005665 | DNA-directed RNA polymerase II, core complex | 23 | 8 | 4.642398 | 0.000172 |
| GO:0016161 | beta-amylase activity | 13 | 6 | 6.160105 | 0.00019 |
| GO:0004497 | monooxygenase activity | 155 | 25 | 2.152725 | 0.000216 |
| GO:0030598 | rRNA N-glycosylase activity | 9 | 5 | 7.414941 | 0.000229 |
| GO:0008152 | metabolic process | 555 | 64 | 1.539101 | 0.000365 |
| GO:0009407 | toxin catabolic process | 20 | 7 | 4.671413 | 0.000424 |
| GO:0008061 | chitin binding | 26 | 8 | 4.106736 | 0.000448 |
| GO:0016301 | kinase activity | 308 | 40 | 1.733363 | 0.000462 |
| GO:0030247 | polysaccharide binding | 94 | 17 | 2.4138 | 0.000561 |
| GO:0004568 | chitinase activity | 28 | 8 | 3.813398 | 0.000778 |
| GO:0006032 | chitin catabolic process | 28 | 8 | 3.813398 | 0.000778 |
| GO:0055114 | oxidation-reduction process | 1464 | 141 | 1.285459 | 0.000998 |

FI910vFU910

None

FI10vFI8

| GO_ID | GO_DESC | UNIV_COUNT | DEG_COUNT | OBS_to_EXP | PVal |
| --- | --- | --- | --- | --- | --- |
| GO:0044444 | cytoplasmic part | 117 | 24 | 3.069269 | 6.80E-07 |
| GO:0006810 | transport | 306 | 44 | 2.151497 | 1.22E-06 |
| GO:0044765 | single-organism transport | 25 | 9 | 5.386567 | 1.98E-05 |
| GO:0003824 | catalytic activity | 384 | 48 | 1.870336 | 2.06E-05 |
| GO:0043231 | intracellular membrane-bounded organelle | 208 | 31 | 2.230016 | 2.17E-05 |
| GO:0004803 | transposase activity | 7 | 5 | 10.68763 | 2.48E-05 |
| GO:0006313 | transposition, DNA-mediated | 7 | 5 | 10.68763 | 2.48E-05 |
| GO:0044237 | cellular metabolic process | 75 | 16 | 3.19204 | 2.87E-05 |
| GO:0043167 | ion binding | 85 | 16 | 2.816506 | 0.000141 |
| GO:0044699 | single-organism process | 123 | 20 | 2.432957 | 0.000184 |
| GO:0006833 | water transport | 16 | 6 | 5.611007 | 0.000393 |
| GO:0042744 | hydrogen peroxide catabolic process | 93 | 16 | 2.574226 | 0.000411 |
| GO:0015250 | water channel activity | 17 | 6 | 5.280948 | 0.000574 |

FI10vFI9

| GO_ID | GO_DESC | UNIV_COUNT | DEG_COUNT | OBS_to_EXP | PVal |
| --- | --- | --- | --- | --- | --- |
| GO:0044444 | cytoplasmic part | 117 | 22 | 3.940856 | 3.06E-08 |
| GO:0044237 | cellular metabolic process | 75 | 16 | 4.47108 | 3.81E-07 |
| GO:0044699 | single-organism process | 123 | 20 | 3.407835 | 1.44E-06 |
| GO:0003824 | catalytic activity | 384 | 40 | 2.183145 | 3.12E-06 |
| GO:0004803 | transposase activity | 7 | 5 | 14.97013 | 4.74E-06 |
| GO:0006313 | transposition, DNA-mediated | 7 | 5 | 14.97013 | 4.74E-06 |
| GO:0044765 | single-organism transport | 25 | 8 | 6.70662 | 1.36E-05 |
| GO:0006810 | transport | 306 | 30 | 2.054724 | 0.000156 |
| GO:0008652 | cellular amino acid biosynthetic process | 14 | 5 | 7.485067 | 0.000341 |
| GO:0019752 | carboxylic acid metabolic process | 14 | 5 | 7.485067 | 0.000341 |
| GO:0043167 | ion binding | 85 | 12 | 2.958803 | 0.000685 |

FI10vFU10

| GO_ID | GO_DESC | UNIV_COUNT | DEG_COUNT | OBS_to_EXP | PVal |
| --- | --- | --- | --- | --- | --- |
| GO:0005488 | binding | 791 | 67 | 1.77316 | 3.66E-06 |
| GO:0022627 | cytosolic small ribosomal subunit | 99 | 14 | 2.960346 | 0.000251 |
| GO:0006259 | DNA metabolic process | 144 | 17 | 2.47136 | 0.000521 |

FILatevFI8

| GO_ID | GO_DESC | UNIV_COUNT | DEG_COUNT | OBS_to_EXP | PVal |
| --- | --- | --- | --- | --- | --- |
| GO:0003824 | catalytic activity | 384 | 54 | 2.524953 | 1.09E-10 |
| GO:0044699 | single-organism process | 123 | 22 | 3.211503 | 1.07E-06 |
| GO:0044237 | cellular metabolic process | 75 | 16 | 3.830448 | 2.91E-06 |
| GO:0006810 | transport | 306 | 35 | 2.053702 | 4.36E-05 |
| GO:0044444 | cytoplasmic part | 117 | 17 | 2.608879 | 0.000257 |
| GO:0044710 | single-organism metabolic process | 89 | 14 | 2.824417 | 0.000386 |
| GO:0006796 | phosphate-containing compound metabolic process | 28 | 7 | 4.488806 | 0.000686 |

FILatevFI9

| GO_ID | GO_DESC | UNIV_COUNT | DEG_COUNT | OBS_to_EXP | PVal |
| --- | --- | --- | --- | --- | --- |
| GO:0003824 | catalytic activity | 384 | 59 | 2.42992 | 1.54E-10 |
| GO:0006810 | transport | 306 | 38 | 1.963963 | 5.27E-05 |
| GO:0044237 | cellular metabolic process | 75 | 15 | 3.163015 | 5.93E-05 |
| GO:0044444 | cytoplasmic part | 117 | 19 | 2.56826 | 0.000132 |
| GO:0006796 | phosphate-containing compound metabolic process | 28 | 8 | 4.518593 | 0.000248 |
| GO:0044699 | single-organism process | 123 | 19 | 2.442979 | 0.000257 |

FILatevFI10

None

FILatevFIFS

| GO_ID | GO_DESC | UNIV_COUNT | DEG_COUNT | OBS_to_EXP | PVal |
| --- | --- | --- | --- | --- | --- |
| GO:0003824 | catalytic activity | 384 | 42 | 2.047377 | 8.58E-06 |
| GO:0044710 | single-organism metabolic process | 89 | 14 | 2.944543 | 0.000253 |
| GO:0044237 | cellular metabolic process | 75 | 12 | 2.995021 | 0.000585 |
| GO:0005488 | binding | 791 | 64 | 1.514549 | 0.000598 |

MaleFemaleInfected10vFU10

None

MIFSvFIFS

| GO_ID | GO_DESC | UNIV_COUNT | DEG_COUNT | OBS_to_EXP | PVal |
| --- | --- | --- | --- | --- | --- |
| GO:0003824 | catalytic activity | 384 | 59 | 3.652884 | -1.49E-10 |
| GO:0044237 | cellular metabolic process | 75 | 25 | 7.924901 | 7.63E-10 |
| GO:0044699 | single-organism process | 123 | 27 | 5.218837 | 8.43E-10 |
| GO:0004803 | transposase activity | 7 | 6 | 20.37832 | 3.70E-08 |
| GO:0006313 | transposition, DNA-mediated | 7 | 6 | 20.37832 | 3.70E-08 |
| GO:0043167 | ion binding | 85 | 16 | 4.475238 | 4.28E-07 |
| GO:0044444 | cytoplasmic part | 117 | 18 | 3.657647 | 1.85E-06 |
| GO:0044710 | single-organism metabolic process | 89 | 15 | 4.006973 | 4.17E-06 |
| GO:0071704 | organic substance metabolic process | 59 | 12 | 4.835533 | 5.10E-06 |
| GO:1901363 | heterocyclic compound binding | 151 | 20 | 3.148967 | 5.46E-06 |
| GO:0006810 | transport | 306 | 31 | 2.408548 | 5.89E-06 |
| GO:0097159 | organic cyclic compound binding | 153 | 20 | 3.107804 | 6.68E-06 |
| GO:0008652 | cellular amino acid biosynthetic process | 14 | 6 | 10.18916 | 1.22E-05 |
| GO:0008152 | metabolic process | 555 | 45 | 1.927679 | 2.06E-05 |
| GO:0044238 | primary metabolic process | 144 | 18 | 2.971838 | 3.52E-05 |
| GO:0044765 | single-organism transport | 25 | 7 | 6.656917 | 5.60E-05 |
| GO:0006796 | phosphate-containing compound metabolic process | 28 | 7 | 5.943676 | 0.000124 |
| GO:0016620 | oxidoreductase activity, acting on the aldehyde or oxo group of donors, NAD or NADP as acceptor | 21 | 6 | 6.792772 | 0.000171 |
| GO:0019752 | carboxylic acid metabolic process | 14 | 5 | 8.490966 | 0.000189 |
| GO:0009536 | plastid | 171 | 18 | 2.5026 | 0.000323 |

MIFSvMI8

| GO_ID | GO_DESC | UNIV_COUNT | DEG_COUNT | OBS_to_EXP | PVal |
| --- | --- | --- | --- | --- | --- |
| GO:0003824 | catalytic activity | 384 | 58 | 3.916016 | 3.84E-10 |
| GO:0044699 | single-organism process | 123 | 26 | 5.480446 | 1.23E-09 |
| GO:0044237 | cellular metabolic process | 75 | 23 | 7.950862 | 1.40E-09 |
| GO:0004803 | transposase activity | 7 | 6 | 22.22291 | 2.26E-08 |
| GO:0006313 | transposition, DNA-mediated | 7 | 6 | 22.22291 | 2.26E-08 |
| GO:0071704 | organic substance metabolic process | 59 | 14 | 6.152104 | 3.80E-08 |
| GO:0044444 | cytoplasmic part | 117 | 19 | 4.210323 | 1.09E-07 |
| GO:0006810 | transport | 306 | 31 | 2.626564 | 9.91E-07 |
| GO:0044765 | single-organism transport | 25 | 8 | 8.296552 | 2.84E-06 |
| GO:1901363 | heterocyclic compound binding | 151 | 19 | 3.262303 | 5.79E-06 |
| GO:0097159 | organic cyclic compound binding | 153 | 19 | 3.219659 | 7.03E-06 |
| GO:0044710 | single-organism metabolic process | 89 | 14 | 4.078361 | 7.42E-06 |
| GO:0008652 | cellular amino acid biosynthetic process | 14 | 6 | 11.11145 | 7.42E-06 |
| GO:0043167 | ion binding | 85 | 13 | 3.965264 | 2.14E-05 |
| GO:0008152 | metabolic process | 555 | 42 | 1.962022 | 2.59E-05 |
| GO:0006796 | phosphate-containing compound metabolic process | 28 | 7 | 6.481681 | 7.18E-05 |
| GO:0016620 | oxidoreductase activity, acting on the aldehyde or oxo group of donors, NAD or NADP as acceptor | 21 | 6 | 7.407635 | 0.000106 |
| GO:0019752 | carboxylic acid metabolic process | 14 | 5 | 9.259544 | 0.000126 |
| GO:0044238 | primary metabolic process | 144 | 16 | 2.880747 | 0.000141 |
| GO:0016887 | ATPase activity | 154 | 16 | 2.693686 | 0.000306 |
| GO:0006820 | anion transport | 18 | 5 | 7.201868 | 0.000474 |
| GO:0009536 | plastid | 171 | 16 | 2.425892 | 0.000972 |
| GO:0044464 | cell part | 80 | 10 | 3.240841 | 0.000984 |

MIFSvMI9

| GO_ID | GO_DESC | UNIV_COUNT | DEG_COUNT | OBS_to_EXP | PVal |
| --- | --- | --- | --- | --- | --- |
| GO:0003824 | catalytic activity | 384 | 42 | 3.188484 | 2.49E-11 |
| GO:0044237 | cellular metabolic process | 75 | 18 | 6.996446 | 1.04E-09 |
| GO:0044699 | single-organism process | 123 | 20 | 4.740139 | 7.75E-09 |
| GO:0044444 | cytoplasmic part | 117 | 18 | 4.484901 | 9.60E-08 |
| GO:0004803 | transposase activity | 7 | 5 | 20.82276 | 9.28E-07 |
| GO:0006313 | transposition, DNA-mediated | 7 | 5 | 20.82276 | 9.28E-07 |
| GO:0006412 | translation | 404 | 34 | 2.453374 | 1.48E-06 |
| GO:0006810 | transport | 306 | 28 | 2.66749 | 2.53E-06 |
| GO:0016620 | oxidoreductase activity, acting on the aldehyde or oxo group of donors, NAD or NADP as acceptor | 21 | 7 | 9.717286 | 4.13E-06 |
| GO:0022627 | cytosolic small ribosomal subunit | 99 | 14 | 4.122485 | 6.97E-06 |
| GO:0071704 | organic substance metabolic process | 59 | 10 | 4.940993 | 2.89E-05 |
| GO:0043167 | ion binding | 85 | 12 | 4.115556 | 3.19E-05 |
| GO:1901363 | heterocyclic compound binding | 151 | 16 | 3.088939 | 6.37E-05 |
| GO:0008652 | cellular amino acid biosynthetic process | 14 | 5 | 10.41138 | 7.23E-05 |
| GO:0097159 | organic cyclic compound binding | 153 | 16 | 3.04856 | 7.46E-05 |
| GO:0044765 | single-organism transport | 25 | 6 | 6.996446 | 0.000161 |
| GO:0003735 | structural constituent of ribosome | 433 | 30 | 2.019759 | 0.000224 |
| GO:0044710 | single-organism metabolic process | 89 | 11 | 3.603039 | 0.000228 |
| GO:0009536 | plastid | 171 | 15 | 2.557181 | 0.000828 |

MIFSvMI10

| GO_ID | GO_DESC | UNIV_COUNT | DEG_COUNT | OBS_to_EXP | PVal |
| --- | --- | --- | --- | --- | --- |
| GO:0003824 | catalytic activity | 384 | 44 | 3.185911 | -4.23E-11 |
| GO:0044237 | cellular metabolic process | 75 | 18 | 6.673035 | 7.97E-10 |
| GO:0044699 | single-organism process | 123 | 21 | 4.747078 | 3.60E-09 |
| GO:0044444 | cytoplasmic part | 117 | 18 | 4.277587 | 1.93E-07 |
| GO:0006412 | translation | 404 | 36 | 2.477612 | 5.69E-07 |
| GO:0004803 | transposase activity | 7 | 5 | 19.86022 | 1.17E-06 |
| GO:0006313 | transposition, DNA-mediated | 7 | 5 | 19.86022 | 1.17E-06 |
| GO:0008652 | cellular amino acid biosynthetic process | 14 | 6 | 11.91613 | 4.96E-06 |
| GO:0016620 | oxidoreductase activity, acting on the aldehyde or oxo group of donors, NAD or NADP as acceptor | 21 | 7 | 9.268105 | 5.64E-06 |
| GO:0022627 | cytosolic small ribosomal subunit | 99 | 14 | 3.931923 | 1.19E-05 |
| GO:0044765 | single-organism transport | 25 | 7 | 7.785208 | 2.06E-05 |
| GO:0071704 | organic substance metabolic process | 59 | 10 | 4.712596 | 4.32E-05 |
| GO:0006810 | transport | 306 | 26 | 2.362458 | 4.74E-05 |
| GO:0009536 | plastid | 171 | 17 | 2.764172 | 0.00015 |
| GO:0043167 | ion binding | 85 | 11 | 3.598205 | 0.000228 |
| GO:0044710 | single-organism metabolic process | 89 | 11 | 3.436488 | 0.000342 |
| GO:1901363 | heterocyclic compound binding | 151 | 15 | 2.762018 | 0.000364 |
| GO:0097159 | organic cyclic compound binding | 153 | 15 | 2.725913 | 0.000419 |
| GO:0003735 | structural constituent of ribosome | 433 | 30 | 1.926396 | 0.000494 |

MIFSvMUFS

| GO_ID | GO_DESC | UNIV_COUNT | DEG_COUNT | OBS_to_EXP | PVal |
| --- | --- | --- | --- | --- | --- |
| GO:0003824 | catalytic activity | 384 | 56 | 3.565803 | 1.42E-10 |
| GO:0044237 | cellular metabolic process | 75 | 22 | 7.172358 | 6.98E-10 |
| GO:0044699 | single-organism process | 123 | 27 | 5.367341 | 8.95E-10 |
| GO:0004803 | transposase activity | 7 | 6 | 20.95819 | 3.13E-08 |
| GO:0006313 | transposition, DNA-mediated | 7 | 6 | 20.95819 | 3.13E-08 |
| GO:0044444 | cytoplasmic part | 117 | 20 | 4.179696 | 5.48E-08 |
| GO:0044765 | single-organism transport | 25 | 9 | 8.802439 | 3.46E-07 |
| GO:0006810 | transport | 306 | 31 | 2.477084 | 3.34E-06 |
| GO:1901363 | heterocyclic compound binding | 151 | 20 | 3.238572 | 3.59E-06 |
| GO:0097159 | organic cyclic compound binding | 153 | 20 | 3.196238 | 4.40E-06 |
| GO:0043167 | ion binding | 85 | 14 | 4.02726 | 8.32E-06 |
| GO:0008652 | cellular amino acid biosynthetic process | 14 | 6 | 10.47909 | 1.04E-05 |
| GO:0044710 | single-organism metabolic process | 89 | 13 | 3.571526 | 6.43E-05 |
| GO:0008152 | metabolic process | 555 | 42 | 1.850363 | 9.77E-05 |
| GO:0006796 | phosphate-containing compound metabolic process | 28 | 7 | 6.112805 | 0.000104 |
| GO:0071704 | organic substance metabolic process | 59 | 10 | 4.144274 | 0.000126 |
| GO:0016620 | oxidoreductase activity, acting on the aldehyde or oxo group of donors, NAD or NADP as acceptor | 21 | 6 | 6.986063 | 0.000147 |
| GO:0019752 | carboxylic acid metabolic process | 14 | 5 | 8.732578 | 0.000166 |
| GO:0044763 | single-organism cellular process | 59 | 9 | 3.729847 | 0.000612 |
| GO:0006820 | anion transport | 18 | 5 | 6.792005 | 0.00062 |

MI89FSvMU89FS

None

MI89FSLatevMU89FS

None

MI8vFI8

| GO_ID | GO_DESC | UNIV_COUNT | DEG_COUNT | OBS_to_EXP | PVal |
| --- | --- | --- | --- | --- | --- |
| GO:0003824 | catalytic activity | 384 | 59 | 2.67104 | 2.00E-10 |
| GO:0015074 | DNA integration | 499 | 68 | 2.369015 | 3.97E-10 |
| GO:0005488 | binding | 791 | 105 | 2.307663 | 5.10E-10 |
| GO:0008152 | metabolic process | 555 | 68 | 2.12998 | 3.71E-09 |
| GO:0044710 | single-organism metabolic process | 89 | 20 | 3.906605 | 1.18E-07 |
| GO:0044237 | cellular metabolic process | 75 | 18 | 4.172254 | 1.74E-07 |
| GO:0003676 | nucleic acid binding | 1046 | 94 | 1.562269 | 9.61E-06 |
| GO:0006537 | glutamate biosynthetic process | 11 | 6 | 9.482396 | 1.28E-05 |
| GO:0009987 | cellular process | 299 | 35 | 2.034962 | 5.20E-05 |
| GO:0044763 | single-organism cellular process | 59 | 12 | 3.535809 | 0.000112 |
| GO:0016040 | glutamate synthase (NADH) activity | 10 | 5 | 8.692197 | 0.000123 |
| GO:0016020 | membrane | 1886 | 143 | 1.318117 | 0.000302 |
| GO:0044238 | primary metabolic process | 144 | 19 | 2.293774 | 0.000604 |
| GO:0044699 | single-organism process | 123 | 17 | 2.402721 | 0.000675 |
| GO:0043170 | macromolecule metabolic process | 62 | 11 | 3.084328 | 0.000736 |
| GO:0006796 | phosphate-containing compound metabolic process | 28 | 7 | 4.346098 | 0.000832 |

MI8vMU8

| GO_ID | GO_DESC | UNIV_COUNT | DEG_COUNT | OBS_to_EXP | PVal |
| --- | --- | --- | --- | --- | --- |
| GO:0003824 | catalytic activity | 384 | 56 | 2.4756 | 6.67E-10 |
| GO:0044237 | cellular metabolic process | 75 | 19 | 4.30047 | 4.61E-08 |
| GO:0005488 | binding | 791 | 85 | 1.824173 | 4.85E-08 |
| GO:0044710 | single-organism metabolic process | 89 | 20 | 3.814728 | 1.73E-07 |
| GO:0071704 | organic substance metabolic process | 59 | 16 | 4.603537 | 1.87E-07 |
| GO:0015074 | DNA integration | 499 | 59 | 2.007128 | 2.51E-07 |
| GO:0008152 | metabolic process | 555 | 63 | 1.926953 | 4.22E-07 |
| GO:0016709 | oxidoreductase activity, acting on paired donors, with incorporation or reduction of molecular oxygen, NADH or NADPH as one donor, and incorporation of one atom of oxygen | 93 | 19 | 3.468121 | 1.64E-06 |
| GO:0016491 | oxidoreductase activity | 506 | 55 | 1.845167 | 8.39E-06 |
| GO:0044550 | secondary metabolite biosynthetic process | 95 | 17 | 3.037728 | 3.57E-05 |
| GO:0009987 | cellular process | 299 | 36 | 2.043878 | 3.68E-05 |
| GO:0016020 | membrane | 1886 | 149 | 1.341122 | 9.99E-05 |
| GO:0051213 | dioxygenase activity | 77 | 13 | 2.866 | 0.00052 |
| GO:0044238 | primary metabolic process | 144 | 19 | 2.239828 | 0.000804 |
| GO:0006796 | phosphate-containing compound metabolic process | 28 | 7 | 4.243885 | 0.000958 |

MI9vFI9

| GO_ID | GO_DESC | UNIV_COUNT | DEG_COUNT | OBS_to_EXP | PVal |
| --- | --- | --- | --- | --- | --- |
| GO:0003824 | catalytic activity | 384 | 50 | 2.654926 | 7.74E-10 |
| GO:0005488 | binding | 791 | 78 | 2.010628 | 3.51E-09 |
| GO:0044237 | cellular metabolic process | 75 | 14 | 3.806102 | 1.44E-05 |
| GO:0022627 | cytosolic small ribosomal subunit | 99 | 15 | 3.089368 | 9.24E-05 |
| GO:0008152 | metabolic process | 555 | 47 | 1.726706 | 0.000194 |
| GO:0006259 | DNA metabolic process | 144 | 18 | 2.548729 | 0.000244 |
| GO:0009987 | cellular process | 299 | 29 | 1.977609 | 0.000377 |
| GO:0044710 | single-organism metabolic process | 89 | 13 | 2.97829 | 0.000385 |
| GO:1901363 | heterocyclic compound binding | 151 | 18 | 2.430576 | 0.000439 |
| GO:0097159 | organic cyclic compound binding | 153 | 18 | 2.398804 | 0.000515 |
| GO:0003735 | structural constituent of ribosome | 433 | 37 | 1.742318 | 0.000762 |
| GO:0006412 | translation | 404 | 35 | 1.766446 | 0.000821 |

MI9vMI8

| GO_ID | GO_DESC | UNIV_COUNT | DEG_COUNT | OBS_to_EXP | PVal |
| --- | --- | --- | --- | --- | --- |
| GO:0005488 | binding | 791 | 76 | 1.967968 | 1.19E-08 |
| GO:0009987 | cellular process | 299 | 36 | 2.466109 | 5.59E-07 |
| GO:0003824 | catalytic activity | 384 | 40 | 2.133584 | 5.42E-06 |
| GO:0043170 | macromolecule metabolic process | 62 | 11 | 3.633975 | 0.000182 |
| GO:0005215 | transporter activity | 183 | 21 | 2.35044 | 0.000244 |
| GO:0006796 | phosphate-containing compound metabolic process | 28 | 7 | 5.120602 | 0.00031 |
| GO:0008152 | metabolic process | 555 | 46 | 1.697641 | 0.00033 |
| GO:0015074 | DNA integration | 499 | 42 | 1.72397 | 0.000431 |
| GO:0044238 | primary metabolic process | 144 | 17 | 2.418062 | 0.000666 |
| GO:0044237 | cellular metabolic process | 75 | 11 | 3.004086 | 0.000988 |

MI9vMU9

| GO_ID | GO_DESC | UNIV_COUNT | DEG_COUNT | OBS_to_EXP | PVal |
| --- | --- | --- | --- | --- | --- |
| GO:0051213 | dioxygenase activity | 77 | 16 | 4.170868 | 9.86E-07 |
| GO:0005488 | binding | 791 | 67 | 1.700182 | 1.46E-05 |
| GO:0004674 | protein serine/threonine kinase activity | 690 | 57 | 1.658147 | 0.000125 |
| GO:0005506 | iron ion binding | 290 | 30 | 2.076445 | 0.000128 |
| GO:0015074 | DNA integration | 499 | 44 | 1.769902 | 0.000178 |
| GO:0006468 | protein phosphorylation | 945 | 71 | 1.508078 | 0.000344 |
| GO:0046872 | metal ion binding | 1129 | 81 | 1.440085 | 0.00056 |
| GO:0016709 | oxidoreductase activity, acting on paired donors, with incorporation or reduction of molecular oxygen, NADH or NADPH as one donor, and incorporation of one atom of oxygen | 93 | 13 | 2.805806 | 0.000688 |
| GO:0006857 | oligopeptide transport | 41 | 8 | 3.916547 | 0.000811 |
| GO:0005215 | transporter activity | 183 | 20 | 2.193694 | 0.00082 |
| GO:0044550 | secondary metabolite biosynthetic process | 95 | 13 | 2.746736 | 0.000844 |
| GO:0016491 | oxidoreductase activity | 506 | 42 | 1.66608 | 0.000855 |

MI10vFI10

| GO_ID | GO_DESC | UNIV_COUNT | DEG_COUNT | OBS_to_EXP | PVal |
| --- | --- | --- | --- | --- | --- |
| GO:0003735 | structural constituent of ribosome | 433 | 40 | 2.179052 | 3.40E-06 |
| GO:0022627 | cytosolic small ribosomal subunit | 99 | 15 | 3.573975 | 1.75E-05 |
| GO:0022625 | cytosolic large ribosomal subunit | 143 | 18 | 2.969148 | 3.55E-05 |
| GO:0006412 | translation | 404 | 34 | 1.985149 | 0.000118 |
| GO:0005488 | binding | 791 | 55 | 1.640143 | 0.000217 |

MI10vMI8

| GO_ID | GO_DESC | UNIV_COUNT | DEG_COUNT | OBS_to_EXP | PVal |
| --- | --- | --- | --- | --- | --- |
| GO:0009987 | cellular process | 299 | 37 | 2.475602 | 3.49E-07 |
| GO:0003824 | catalytic activity | 384 | 42 | 2.188106 | 1.64E-06 |
| GO:0005488 | binding | 791 | 70 | 1.770402 | 2.32E-06 |
| GO:0015074 | DNA integration | 499 | 45 | 1.804107 | 9.73E-05 |
| GO:0009607 | response to biotic stimulus | 27 | 7 | 5.186622 | 0.00028 |
| GO:0006796 | phosphate-containing compound metabolic process | 28 | 7 | 5.001386 | 0.000358 |
| GO:0000166 | nucleotide binding | 700 | 55 | 1.571864 | 0.000607 |
| GO:0005215 | transporter activity | 183 | 20 | 2.186398 | 0.000855 |
| GO:0043170 | macromolecule metabolic process | 62 | 10 | 3.226701 | 0.000935 |
| GO:0008152 | metabolic process | 555 | 45 | 1.622071 | 0.000987 |

MIFIFSvMIFI8

| GO_ID | GO_DESC | UNIV_COUNT | DEG_COUNT | OBS_to_EXP | PVal |
| --- | --- | --- | --- | --- | --- |
| GO:0003824 | catalytic activity | 384 | 61 | 4.389776 | 2.46E-10 |
| GO:0044237 | cellular metabolic process | 75 | 25 | 9.211332 | 7.44E-10 |
| GO:0044699 | single-organism process | 123 | 26 | 5.841333 | 8.65E-10 |
| GO:0004803 | transposase activity | 7 | 6 | 23.68628 | 1.56E-08 |
| GO:0006313 | transposition, DNA-mediated | 7 | 6 | 23.68628 | 1.56E-08 |
| GO:0071704 | organic substance metabolic process | 59 | 14 | 6.55722 | 1.71E-08 |
| GO:0006810 | transport | 306 | 33 | 2.980137 | 2.34E-08 |
| GO:1901363 | heterocyclic compound binding | 151 | 22 | 4.026145 | 2.67E-08 |
| GO:0097159 | organic cyclic compound binding | 153 | 22 | 3.973516 | 3.39E-08 |
| GO:0044444 | cytoplasmic part | 117 | 19 | 4.487572 | 4.07E-08 |
| GO:0043167 | ion binding | 85 | 15 | 4.876588 | 3.50E-07 |
| GO:0044765 | single-organism transport | 25 | 8 | 8.842879 | 1.76E-06 |
| GO:0044238 | primary metabolic process | 144 | 18 | 3.45425 | 4.65E-06 |
| GO:0008652 | cellular amino acid biosynthetic process | 14 | 6 | 11.84314 | 5.14E-06 |
| GO:0044710 | single-organism metabolic process | 89 | 13 | 4.036427 | 1.81E-05 |
| GO:0008152 | metabolic process | 555 | 40 | 1.991639 | 2.89E-05 |
| GO:0006796 | phosphate-containing compound metabolic process | 28 | 7 | 6.908499 | 4.80E-05 |
| GO:0016620 | oxidoreductase activity, acting on the aldehyde or oxo group of donors, NAD or NADP as acceptor | 21 | 6 | 7.895428 | 7.48E-05 |
| GO:0019752 | carboxylic acid metabolic process | 14 | 5 | 9.869285 | 9.32E-05 |
| GO:0016887 | ATPase activity | 154 | 16 | 2.871065 | 0.000149 |
| GO:0009536 | plastid | 171 | 17 | 2.747239 | 0.000161 |
| GO:0006820 | anion transport | 18 | 5 | 7.67611 | 0.000354 |

MIFIFSvMIFI9

| GO_ID | GO_DESC | UNIV_COUNT | DEG_COUNT | OBS_to_EXP | PVal |
| --- | --- | --- | --- | --- | --- |
| GO:0003824 | catalytic activity | 384 | 54 | 4.112768 | -5.63E-11 |
| GO:0044699 | single-organism process | 123 | 25 | 5.944381 | 7.74E-10 |
| GO:0044237 | cellular metabolic process | 75 | 23 | 8.968882 | 1.05E-09 |
| GO:0006810 | transport | 306 | 33 | 3.154018 | 6.16E-09 |
| GO:0004803 | transposase activity | 7 | 6 | 25.0683 | 1.13E-08 |
| GO:0006313 | transposition, DNA-mediated | 7 | 6 | 25.0683 | 1.13E-08 |
| GO:0044444 | cytoplasmic part | 117 | 18 | 4.499439 | 9.14E-08 |
| GO:0043167 | ion binding | 85 | 14 | 4.817046 | 1.04E-06 |
| GO:0008652 | cellular amino acid biosynthetic process | 14 | 6 | 12.53415 | 3.70E-06 |
| GO:0016620 | oxidoreductase activity, acting on the aldehyde or oxo group of donors, NAD or NADP as acceptor | 21 | 7 | 9.748784 | 4.04E-06 |
| GO:1901363 | heterocyclic compound binding | 151 | 18 | 3.48632 | 4.19E-06 |
| GO:0071704 | organic substance metabolic process | 59 | 11 | 5.45271 | 4.30E-06 |
| GO:0097159 | organic cyclic compound binding | 153 | 18 | 3.440747 | 5.05E-06 |
| GO:0044765 | single-organism transport | 25 | 7 | 8.188979 | 1.48E-05 |
| GO:0008152 | metabolic process | 555 | 39 | 2.055149 | 1.81E-05 |
| GO:0044710 | single-organism metabolic process | 89 | 12 | 3.943329 | 4.92E-05 |
| GO:0009536 | plastid | 171 | 17 | 2.907532 | 8.16E-05 |
| GO:0006796 | phosphate-containing compound metabolic process | 28 | 6 | 6.267076 | 0.000309 |
| GO:0044238 | primary metabolic process | 144 | 14 | 2.843395 | 0.000426 |

MIFIFSvMIFI10

| GO_ID | GO_DESC | UNIV_COUNT | DEG_COUNT | OBS_to_EXP | PVal |
| --- | --- | --- | --- | --- | --- |
| GO:0003824 | catalytic activity | 384 | 54 | 3.922068 | -5.11E-10 |
| GO:0044237 | cellular metabolic process | 75 | 23 | 8.553014 | 1.55E-10 |
| GO:0044699 | single-organism process | 123 | 26 | 5.895503 | 3.09E-10 |
| GO:0004803 | transposase activity | 7 | 6 | 23.90594 | 1.44E-08 |
| GO:0006313 | transposition, DNA-mediated | 7 | 6 | 23.90594 | 1.44E-08 |
| GO:0006810 | transport | 306 | 33 | 3.007773 | 1.82E-08 |
| GO:0044444 | cytoplasmic part | 117 | 19 | 4.529188 | 3.48E-08 |
| GO:0043167 | ion binding | 85 | 15 | 4.921811 | 3.11E-07 |
| GO:0044765 | single-organism transport | 25 | 8 | 8.924884 | 1.65E-06 |
| GO:1901363 | heterocyclic compound binding | 151 | 19 | 3.509371 | 2.01E-06 |
| GO:0097159 | organic cyclic compound binding | 153 | 19 | 3.463497 | 2.45E-06 |
| GO:0008652 | cellular amino acid biosynthetic process | 14 | 6 | 11.95297 | 4.87E-06 |
| GO:0016620 | oxidoreductase activity, acting on the aldehyde or oxo group of donors, NAD or NADP as acceptor | 21 | 7 | 9.296754 | 5.53E-06 |
| GO:0071704 | organic substance metabolic process | 59 | 11 | 5.199879 | 6.75E-06 |
| GO:0044710 | single-organism metabolic process | 89 | 13 | 4.073859 | 1.65E-05 |
| GO:0008152 | metabolic process | 555 | 40 | 2.010109 | 2.35E-05 |
| GO:0006796 | phosphate-containing compound metabolic process | 28 | 7 | 6.972566 | 4.52E-05 |
| GO:0019752 | carboxylic acid metabolic process | 14 | 5 | 9.960808 | 8.92E-05 |
| GO:0009536 | plastid | 171 | 17 | 2.772716 | 0.000144 |
| GO:0044238 | primary metabolic process | 144 | 15 | 2.905236 | 0.00021 |
| GO:0006820 | anion transport | 18 | 5 | 7.747295 | 0.000339 |

MIFIFSvMUFS

| GO_ID | GO_DESC | UNIV_COUNT | DEG_COUNT | OBS_to_EXP | PVal |
| --- | --- | --- | --- | --- | --- |
| GO:0005488 | binding | 791 | 17 | 2.585461 | 0.000315 |
| GO:0015074 | DNA integration | 499 | 12 | 2.892986 | 0.000959 |

MIFILatevMIFIFS

| GO_ID | GO_DESC | UNIV_COUNT | DEG_COUNT | OBS_to_EXP | PVal |
| --- | --- | --- | --- | --- | --- |
| GO:0015074 | DNA integration | 499 | 22 | 3.35684 | 7.66E-07 |
| GO:0005488 | binding | 791 | 27 | 2.598937 | 5.68E-06 |
| GO:0003676 | nucleic acid binding | 1046 | 30 | 2.183726 | 4.77E-05 |

MIFILatevMIFI8

| GO_ID | GO_DESC | UNIV_COUNT | DEG_COUNT | OBS_to_EXP | PVal |
| --- | --- | --- | --- | --- | --- |
| GO:0003824 | catalytic activity | 384 | 56 | 4.073626 | -2.05E-10 |
| GO:0044237 | cellular metabolic process | 75 | 23 | 8.566254 | 4.90E-10 |
| GO:0044699 | single-organism process | 123 | 26 | 5.904629 | 5.53E-10 |
| GO:0044444 | cytoplasmic part | 117 | 20 | 4.774946 | 6.77E-09 |
| GO:0004803 | transposase activity | 7 | 6 | 23.94295 | 1.44E-08 |
| GO:0006313 | transposition, DNA-mediated | 7 | 6 | 23.94295 | 1.44E-08 |
| GO:0006810 | transport | 306 | 32 | 2.921144 | 6.07E-08 |
| GO:1901363 | heterocyclic compound binding | 151 | 21 | 3.884783 | 1.03E-07 |
| GO:0044765 | single-organism transport | 25 | 9 | 10.05604 | 1.12E-07 |
| GO:0097159 | organic cyclic compound binding | 153 | 21 | 3.834001 | 1.30E-07 |
| GO:0043167 | ion binding | 85 | 15 | 4.92943 | 3.05E-07 |
| GO:0044710 | single-organism metabolic process | 89 | 15 | 4.707883 | 5.67E-07 |
| GO:0008652 | cellular amino acid biosynthetic process | 14 | 6 | 11.97147 | 4.83E-06 |
| GO:0071704 | organic substance metabolic process | 59 | 11 | 5.207929 | 6.65E-06 |
| GO:0008152 | metabolic process | 555 | 41 | 2.063551 | 1.00E-05 |
| GO:0006796 | phosphate-containing compound metabolic process | 28 | 7 | 6.983359 | 4.48E-05 |
| GO:0016620 | oxidoreductase activity, acting on the aldehyde or oxo group of donors, NAD or NADP as acceptor | 21 | 6 | 7.980982 | 7.04E-05 |
| GO:0016887 | ATPase activity | 154 | 16 | 2.902175 | 0.000132 |
| GO:0009536 | plastid | 171 | 17 | 2.777008 | 0.000142 |
| GO:0044238 | primary metabolic process | 144 | 15 | 2.909733 | 0.000206 |
| GO:0006820 | anion transport | 18 | 5 | 7.759288 | 0.000336 |

MIFILatevMIFI9

| GO_ID | GO_DESC | UNIV_COUNT | DEG_COUNT | OBS_to_EXP | PVal |
| --- | --- | --- | --- | --- | --- |
| GO:0003824 | catalytic activity | 384 | 44 | 3.428949 | -1.96E-10 |
| GO:0044237 | cellular metabolic process | 75 | 18 | 7.18209 | 4.35E-10 |
| GO:0044699 | single-organism process | 123 | 20 | 4.865914 | 5.13E-09 |
| GO:0006810 | transport | 306 | 30 | 2.93386 | 1.47E-07 |
| GO:0044444 | cytoplasmic part | 117 | 17 | 4.348131 | 3.47E-07 |
| GO:0043167 | ion binding | 85 | 14 | 4.928885 | 7.95E-07 |
| GO:0004803 | transposase activity | 7 | 5 | 21.37527 | 8.14E-07 |
| GO:0006313 | transposition, DNA-mediated | 7 | 5 | 21.37527 | 8.14E-07 |
| GO:1901363 | heterocyclic compound binding | 151 | 18 | 3.567263 | 3.04E-06 |
| GO:0008652 | cellular amino acid biosynthetic process | 14 | 6 | 12.82516 | 3.24E-06 |
| GO:0097159 | organic cyclic compound binding | 153 | 18 | 3.520632 | 3.68E-06 |
| GO:0044765 | single-organism transport | 25 | 7 | 8.379104 | 1.28E-05 |
| GO:0044710 | single-organism metabolic process | 89 | 12 | 4.034882 | 3.94E-05 |
| GO:0009536 | plastid | 171 | 17 | 2.975037 | 6.16E-05 |
| GO:0071704 | organic substance metabolic process | 59 | 9 | 4.564887 | 0.000138 |
| GO:0016620 | oxidoreductase activity, acting on the aldehyde or oxo group of donors, NAD or NADP as acceptor | 21 | 5 | 7.125089 | 0.000534 |

MIFILatevMIFI10

| GO_ID | GO_DESC | UNIV_COUNT | DEG_COUNT | OBS_to_EXP | PVal |
| --- | --- | --- | --- | --- | --- |
| GO:0003824 | catalytic activity | 384 | 47 | 3.737111 | -5.34E-10 |
| GO:0044237 | cellular metabolic process | 75 | 20 | 8.142132 | 2.86E-10 |
| GO:0044699 | single-organism process | 123 | 22 | 5.461186 | 4.87E-10 |
| GO:0044444 | cytoplasmic part | 117 | 20 | 5.219315 | 1.79E-09 |
| GO:0004803 | transposase activity | 7 | 6 | 26.17114 | 7.86E-09 |
| GO:0006313 | transposition, DNA-mediated | 7 | 6 | 26.17114 | 7.86E-09 |
| GO:0006810 | transport | 306 | 31 | 3.093212 | 2.70E-08 |
| GO:0044765 | single-organism transport | 25 | 9 | 10.99188 | 5.21E-08 |
| GO:0043167 | ion binding | 85 | 14 | 5.028964 | 6.26E-07 |
| GO:1901363 | heterocyclic compound binding | 151 | 18 | 3.639695 | 2.30E-06 |
| GO:0097159 | organic cyclic compound binding | 153 | 18 | 3.592117 | 2.78E-06 |
| GO:0008652 | cellular amino acid biosynthetic process | 14 | 6 | 13.08557 | 2.89E-06 |
| GO:0071704 | organic substance metabolic process | 59 | 10 | 5.175084 | 1.95E-05 |
| GO:0044710 | single-organism metabolic process | 89 | 12 | 4.116808 | 3.24E-05 |
| GO:0016620 | oxidoreductase activity, acting on the aldehyde or oxo group of donors, NAD or NADP as acceptor | 21 | 6 | 8.723713 | 4.29E-05 |
| GO:0009536 | plastid | 171 | 17 | 3.035444 | 4.81E-05 |
| GO:0008152 | metabolic process | 555 | 36 | 1.980519 | 8.12E-05 |
| GO:0006820 | anion transport | 18 | 5 | 8.481387 | 0.000223 |
| GO:0006796 | phosphate-containing compound metabolic process | 28 | 6 | 6.542785 | 0.000245 |
| GO:0044238 | primary metabolic process | 144 | 13 | 2.756451 | 0.000913 |

MIFI8vMU8

| GO_ID | GO_DESC | UNIV_COUNT | DEG_COUNT | OBS_to_EXP | PVal |
| --- | --- | --- | --- | --- | --- |
| GO:0016020 | membrane | 1886 | 163 | 1.67335 | 2.17E-10 |
| GO:0005488 | binding | 791 | 86 | 2.105053 | 2.31E-10 |
| GO:0015074 | DNA integration | 499 | 58 | 2.250445 | 6.38E-09 |
| GO:0016709 | oxidoreductase activity, acting on paired donors, with incorporation or reduction of molecular oxygen, NADH or NADPH as one donor, and incorporation of one atom of oxygen | 93 | 19 | 3.955593 | 2.25E-07 |
| GO:0004672 | protein kinase activity | 544 | 56 | 1.993105 | 6.75E-07 |
| GO:0020037 | heme binding | 342 | 40 | 2.264513 | 1.20E-06 |
| GO:0044550 | secondary metabolite biosynthetic process | 95 | 18 | 3.668511 | 1.49E-06 |
| GO:0005506 | iron ion binding | 290 | 35 | 2.336743 | 2.71E-06 |
| GO:0006468 | protein phosphorylation | 945 | 81 | 1.659565 | 4.57E-06 |
| GO:0046872 | metal ion binding | 1129 | 93 | 1.594887 | 4.62E-06 |
| GO:0050664 | oxidoreductase activity, acting on NADH or NADPH, oxygen as acceptor | 17 | 7 | 7.972419 | 1.18E-05 |
| GO:0004674 | protein serine/threonine kinase activity | 690 | 62 | 1.739737 | 1.52E-05 |
| GO:0016705 | oxidoreductase activity, acting on paired donors, with incorporation or reduction of molecular oxygen | 162 | 21 | 2.509835 | 9.39E-05 |
| GO:0019538 | protein metabolic process | 33 | 8 | 4.693718 | 0.000215 |
| GO:0016310 | phosphorylation | 436 | 39 | 1.731885 | 0.000618 |
| GO:0016301 | kinase activity | 308 | 30 | 1.885869 | 0.000658 |
| GO:0006310 | DNA recombination | 113 | 15 | 2.570122 | 0.000703 |
| GO:0004601 | peroxidase activity | 137 | 17 | 2.402533 | 0.000703 |

MIFI9vMIFI8

| GO_ID | GO_DESC | UNIV_COUNT | DEG_COUNT | OBS_to_EXP | PVal |
| --- | --- | --- | --- | --- | --- |
| GO:0015074 | DNA integration | 499 | 9 | 3.828952 | 0.000562 |
|  |  |  |  |  |  |

MIFI9vMUFU9

| GO_ID | GO_DESC | UNIV_COUNT | DEG_COUNT | OBS_to_EXP | PVal |
| --- | --- | --- | --- | --- | --- |
| GO:0009987 | cellular process | 299 | 12 | 4.068618 | 4.30E-05 |
| GO:0015074 | DNA integration | 499 | 15 | 3.047387 | 0.000131 |
| GO:0005488 | binding | 791 | 19 | 2.435084 | 0.000313 |
| GO:0003676 | nucleic acid binding | 1046 | 22 | 2.1322 | 0.000647 |

MIFI10vMIFI8

None

MILatevFILate

| GO_ID | GO_DESC | UNIV_COUNT | DEG_COUNT | OBS_to_EXP | PVal |
| --- | --- | --- | --- | --- | --- |
| GO:0003824 | catalytic activity | 384 | 41 | 3.122658 | 5.31E-11 |
| GO:0044237 | cellular metabolic process | 75 | 18 | 7.019125 | 1.10E-09 |
| GO:0044699 | single-organism process | 123 | 18 | 4.279954 | 1.98E-07 |
| GO:0004803 | transposase activity | 7 | 5 | 20.89025 | 9.13E-07 |
| GO:0006313 | transposition, DNA-mediated | 7 | 5 | 20.89025 | 9.13E-07 |
| GO:1901363 | heterocyclic compound binding | 151 | 19 | 3.680005 | 9.97E-07 |
| GO:0043167 | ion binding | 85 | 14 | 4.817046 | 1.04E-06 |
| GO:0097159 | organic cyclic compound binding | 153 | 19 | 3.6319 | 1.22E-06 |
| GO:0044444 | cytoplasmic part | 117 | 16 | 3.999501 | 2.35E-06 |
| GO:0006810 | transport | 306 | 27 | 2.580561 | 7.08E-06 |
| GO:0044765 | single-organism transport | 25 | 7 | 8.188979 | 1.48E-05 |
| GO:0044710 | single-organism metabolic process | 89 | 12 | 3.943329 | 4.92E-05 |
| GO:0008652 | cellular amino acid biosynthetic process | 14 | 5 | 10.44513 | 7.12E-05 |
| GO:0009536 | plastid | 171 | 17 | 2.907532 | 8.16E-05 |
| GO:0071704 | organic substance metabolic process | 59 | 9 | 4.461308 | 0.000164 |
| GO:0044238 | primary metabolic process | 144 | 14 | 2.843395 | 0.000426 |
| GO:0016620 | oxidoreductase activity, acting on the aldehyde or oxo group of donors, NAD or NADP as acceptor | 21 | 5 | 6.963417 | 0.000593 |
| GO:0008152 | metabolic process | 555 | 34 | 1.791668 | 0.000787 |

MILatevMIFS

| GO_ID | GO_DESC | UNIV_COUNT | DEG_COUNT | OBS_to_EXP | PVal |
| --- | --- | --- | --- | --- | --- |
| GO:0043167 | ion binding | 85 | 9 | 5.234649 | 5.46E-05 |
| GO:0044237 | cellular metabolic process | 75 | 8 | 5.273425 | 0.000134 |
| GO:0044699 | single-organism process | 123 | 10 | 4.019379 | 0.000201 |

MILatevMI8

| GO_ID | GO_DESC | UNIV_COUNT | DEG_COUNT | OBS_to_EXP | PVal |
| --- | --- | --- | --- | --- | --- |
| GO:0003824 | catalytic activity | 384 | 55 | 3.692243 | -3.95E-10 |
| GO:0044237 | cellular metabolic process | 75 | 23 | 7.905429 | 2.75E-10 |
| GO:0044699 | single-organism process | 123 | 27 | 5.658711 | 4.66E-10 |
| GO:0044444 | cytoplasmic part | 117 | 21 | 4.626923 | 4.57E-09 |
| GO:0004803 | transposase activity | 7 | 6 | 22.09592 | 2.30E-08 |
| GO:0006313 | transposition, DNA-mediated | 7 | 6 | 22.09592 | 2.30E-08 |
| GO:0044765 | single-organism transport | 25 | 9 | 9.280286 | 2.21E-07 |
| GO:0071704 | organic substance metabolic process | 59 | 13 | 5.680024 | 3.14E-07 |
| GO:0043167 | ion binding | 85 | 15 | 4.54916 | 8.41E-07 |
| GO:1901363 | heterocyclic compound binding | 151 | 20 | 3.41438 | 1.61E-06 |
| GO:0097159 | organic cyclic compound binding | 153 | 20 | 3.369748 | 1.98E-06 |
| GO:0006810 | transport | 306 | 30 | 2.527311 | 3.26E-06 |
| GO:0008652 | cellular amino acid biosynthetic process | 14 | 6 | 11.04796 | 7.67E-06 |
| GO:0044763 | single-organism cellular process | 59 | 11 | 4.806174 | 1.42E-05 |
| GO:0044710 | single-organism metabolic process | 89 | 13 | 3.765409 | 3.74E-05 |
| GO:0008152 | metabolic process | 555 | 41 | 1.904363 | 6.31E-05 |
| GO:0006796 | phosphate-containing compound metabolic process | 28 | 7 | 6.444643 | 7.44E-05 |
| GO:0016620 | oxidoreductase activity, acting on the aldehyde or oxo group of donors, NAD or NADP as acceptor | 21 | 6 | 7.365306 | 0.00011 |
| GO:0044238 | primary metabolic process | 144 | 16 | 2.864286 | 0.00015 |
| GO:0006820 | anion transport | 18 | 5 | 7.160714 | 0.000487 |
| GO:0016887 | ATPase activity | 154 | 15 | 2.5109 | 0.000974 |

MILatevMI9

| GO_ID | GO_DESC | UNIV_COUNT | DEG_COUNT | | OBS_to_EXP | PVal |
| --- | --- | --- | --- | --- | --- | --- |
| GO:0044237 | cellular metabolic process | 75 | | 15 | 6.848197 | 4.14E-09 |
| GO:0044699 | single-organism process | 123 | | 16 | 4.454112 | 5.93E-07 |
| GO:0003824 | catalytic activity | 384 | | 29 | 2.585908 | 3.22E-06 |
| GO:0016620 | oxidoreductase activity, acting on the aldehyde or oxo group of donors, NAD or NADP as acceptor | 21 | | 6 | 9.783139 | 2.25E-05 |
| GO:0044444 | cytoplasmic part | 117 | | 13 | 3.804554 | 3.76E-05 |
| GO:0044765 | single-organism transport | 25 | | 6 | 8.217837 | 6.65E-05 |
| GO:0006810 | transport | 306 | | 22 | 2.46177 | 0.000102 |
| GO:0043167 | ion binding | 85 | | 10 | 4.028351 | 0.000182 |
| GO:0071704 | organic substance metabolic process | 59 | | 8 | 4.642846 | 0.0003 |
| GO:0009536 | plastid | 171 | | 14 | 2.803356 | 0.000508 |

MILatevMI10

| GO_ID | GO_DESC | UNIV_COUNT | DEG_COUNT | OBS_to_EXP | PVal |
| --- | --- | --- | --- | --- | --- |
| GO:0044237 | cellular metabolic process | 75 | 14 | 5.807586 | 1.00E-07 |
| GO:0044699 | single-organism process | 123 | 17 | 4.300042 | 4.19E-07 |
| GO:0043167 | ion binding | 85 | 12 | 4.392292 | 1.68E-05 |
| GO:0003824 | catalytic activity | 384 | 29 | 2.349609 | 1.99E-05 |
| GO:0044444 | cytoplasmic part | 117 | 13 | 3.456897 | 9.93E-05 |
| GO:0044765 | single-organism transport | 25 | 6 | 7.466897 | 0.000113 |
| GO:0071704 | organic substance metabolic process | 59 | 8 | 4.218586 | 0.000568 |
| GO:0006810 | transport | 306 | 21 | 2.135142 | 0.000951 |
